# PNA-ligand conjugates enable site-selective and directional targeting of adjacent G-quadruplexes

**DOI:** 10.1039/d6sc04697e

**Published:** 2026-10-05

**Authors:** Raffaele Graziano, Enrico Cadoni, Simona Marzano, Valentina Arciuolo, Naura Fakhira Antariksa, Marco Di Antonio, Jussara Amato, Annemieke Madder, Bruno Pagano

**Affiliations:** a Department of Pharmacy, University of Naples Federico II Via D. Montesano 49 80131 Naples Italy bruno.pagano@unina.it; b Organic and Biomimetic Chemistry Research Group, Department of Organic and Macromolecular Chemistry, Ghent University Krijgslaan 281-S4 9000 Ghent Belgium annemieke.madder@ugent.be; c Department of Chemistry, Molecular Sciences Research Hub, Imperial College London 82 Wood Lane London W12 0BZ UK

## Abstract

G-quadruplexes (G4s) are non-canonical DNA structures enriched in gene promoters and involved in transcriptional regulation, making them attractive drug targets, albeit difficult to target individually with high specificity due to their structural similarity. In the human *c-KIT* promoter, three adjacent G4 motifs (*c-KIT1*, c-KIT*, and *c-KIT2*) modulate oncogene expression, yet their individual contributions remain poorly understood, posing a significant challenge for selective targeting and underscoring the need for tools capable of discriminating between closely spaced G4s. Here, we report a dual-recognition strategy that combines the sequence specificity of peptide nucleic acids (PNAs) with the stabilizing properties of a G4 ligand to achieve site-selective and programmable targeting of neighboring G4s. Sequence-specific base pairing enables the PNA probe to recognize the flanking region between the *c-KIT1* and c-KIT* motifs, positioning the ligand in proximity to a defined G4 structure and enabling directional and selective stabilization. Ligand conjugation at the N- or C-terminus of the PNA directs selective targeting of individual G4 units, while dual conjugation enables their simultaneous stabilization. This approach provides positional control not accessible with conventional small-molecule ligands and establishes a modular platform for targeting G4s within regions containing closely spaced motifs. A dual luciferase assay revealed that targeted G4 stabilization within the *c-KIT* promoter leads to distinct transcriptional outcomes in cells, indicating distinct regulatory roles of the *c-KIT1* and c-KIT* motifs. This PNA-ligand platform enables site-specific recognition and discrimination between adjacent G4s and provides a general framework for selective chemical intervention in G4-regulated oncogenes.

## Abstract 2

Sequence-specific PNA hybridization directs a conjugated ligand toward selected adjacent *c-KIT* G-quadruplexes, enabling directional stabilization and differential modulation of promoter activity.

## Graphical abstract

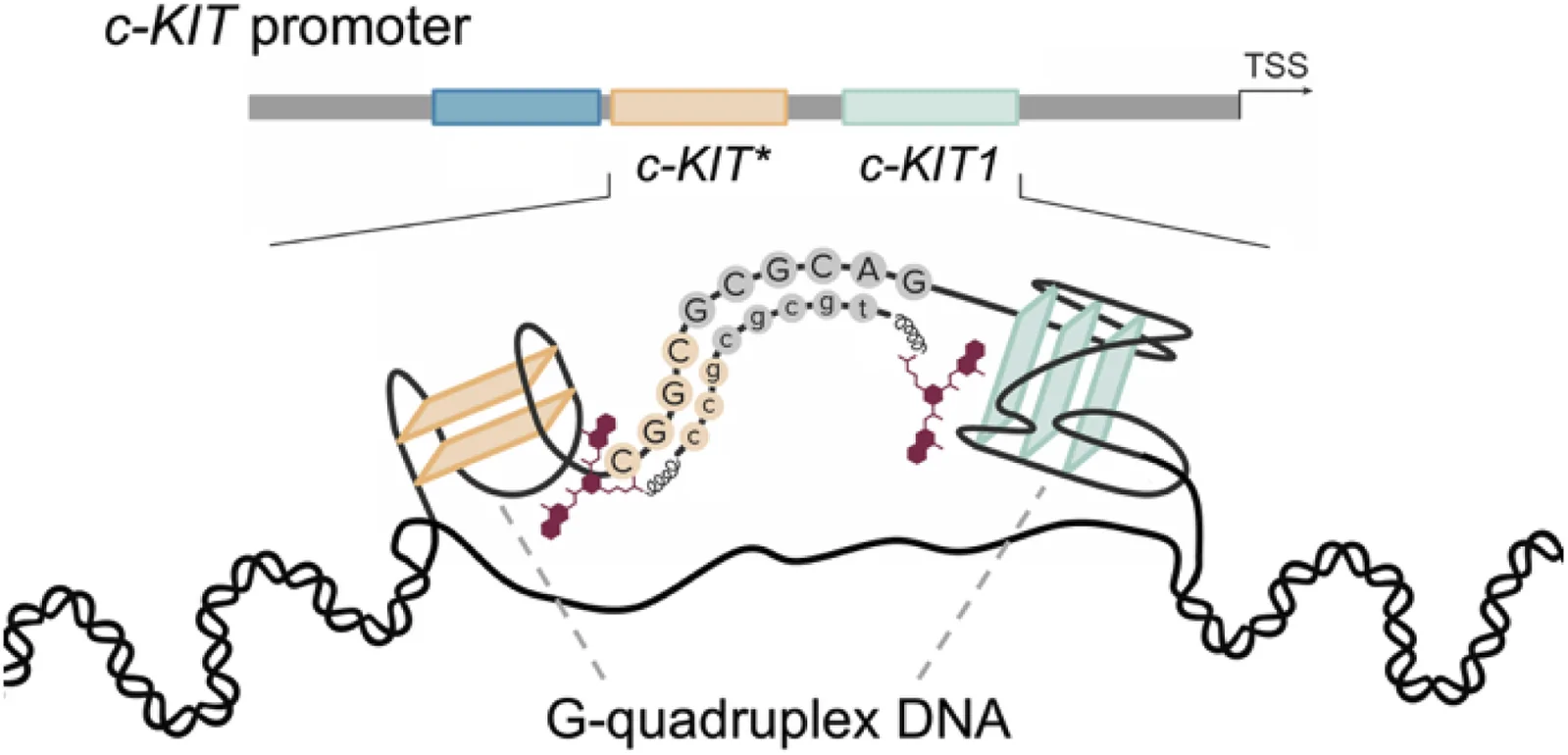

## 1 Introduction

G-quadruplexes (G4s) are nucleic acid secondary structures that arise from DNA or RNA sequences containing runs of guanines (G-tracts) separated by other bases.^1^ Unlike the canonical double helix, which features A–T and G–C base pairs, G4s are stabilized by G-tetrads, planar arrangements of four guanines held together by Hoogsteen hydrogen bonds. These structures are further stabilized by physiologically relevant monovalent cations, such as potassium or sodium, which are accommodated in the central cavity formed by the stacked G-tetrads. Intramolecular G4 folding requires the presence of four (or more) G-tracts in a single strand, giving rise to parallel, antiparallel, or hybrid conformations depending on the orientation of the strands in the structure.

In the human genome, experimentally detected G4-forming sequences are copious, with estimates exceeding 700 000 potential sites.^2^ These sequences frequently occur in key regulatory regions, including telomeres and oncogene promoters, highlighting their involvement in critical biological processes such as genome stability maintenance, cell proliferation, and transcription.^3–6^

The functional role of DNA G4s in cancer-related cellular processes has made them attractive targets for anticancer drug development.^7^ Ligands that bind and stabilize specific oncogene promoter G4s can, for example, selectively regulate their transcription. Knowledge of the 3D structure of a G4 is essential for designing small-molecule ligands and studying the effects of G4 stabilization on gene transcription. Although numerous small molecules have been developed to stabilize G4 structures, achieving true selectivity remains a major challenge. Indeed, many ligands also bind canonical duplex DNA, which is largely prevalent in the genome, and their frequently cationic nature promotes nonspecific electrostatic interactions, severely limiting discrimination among genomic targets.

In addition, the abundance of G4-forming sequences complicates the selective targeting of individual G4 structures. Achieving such selectivity is crucial, especially in therapeutic contexts like cancer treatment, where stabilizing or destabilizing particular G4s can modulate the expression of specific genes. The main challenges lie in the structural similarities among G4s and the polymorphic behavior of the G4-forming DNA, which allows the same sequence to adopt multiple interconverting conformations. Even G4-selective ligands still lack the ability to selectively target specific topologies and sequences, highlighting the need for more precise targeting strategies to translate G4-targeting approaches into clinical use. Identifying selective small-molecule ligands requires careful design, significant synthetic efforts,^8^ and recognition elements that enable precise sequence-specific targeting.^9^ Although in principle discrimination among G4s should be possible, as they are generally inserted into different sequence contexts and exhibit distinct connecting loops and flanking regions, exploiting these differences using small molecules alone remains difficult. Small energy barriers between conformations also allow for easy interconversion, further complicating selective ligand design. Furthermore, most current approaches focus on isolated G4 structures and do not address the additional level of complexity arising from the presence of multiple neighboring G4 motifs within the same regulatory region. Multivalent small-molecule ligands have also been developed to recognize multimeric G4 architectures. For example, dimeric derivatives of PDC (360A) and related bisquinolinium ligands have been developed to target multimeric telomeric G4s.^10,11^ These studies demonstrate that multivalent ligand design can exploit the spatial organization of multiple G4 units. However, they do not provide sequence-specific positional control to direct a ligand preferentially toward a predetermined G4 motif. Recently, chemical modification of Cas9 proteins with G4-ligands has been successfully used to probe individual G4s in the genome and assess their transcriptional response,^12^ although this approach cannot be leveraged for therapeutic applications.

The use of oligonucleotides or their analogs offers an alternative to address the lack of selectivity encountered in small molecule-based strategies, whilst offering a therapeutically viable strategy.^13–15^ Beyond the G-tetrads, which are the core components of all G4 structures, other distinguishing features, such as loops and flanking regions, vary between G4s. These elements potentially make each G4 unique within the genome and provide targets for oligonucleotide-based strategies that can exploit the high specificity of Watson–Crick base-pairing. For example, sufficiently long G4 loops and 5′–/3′-flanking regions may be utilized to target a specific sequence within or adjacent to the desired G4 structure.^16^

However, natural oligonucleotides face significant drawbacks that limit their therapeutic applications, including susceptibility to enzymatic degradation, delivery challenges, and bioavailability issues due to premature degradation in biological systems and rapid renal excretion. Chemical modifications of nucleic acids can help overcome these limitations.^17–21^ Among the synthetic nucleic acid derivatives, peptide nucleic acids (PNA) have unique physicochemical properties that provide them with several advantageous biological characteristics over other modified nucleic acids, due to their backbone modification.^22^ Indeed, PNAs feature a neutral peptide-like backbone instead of the negatively charged phosphodiester backbone found in natural nucleic acids and other analogs, which enhances their stability, resistance to nucleases, and strong binding affinity to complementary DNA or RNA sequences.^23–25^ This higher binding affinity allows the use of shorter probes, which is particularly advantageous in constrained genomic contexts where minimal perturbation of secondary structure elements is required. PNAs also have the ability to invade duplex DNA by recognizing complementary sequences to form stable PNA–DNA hybrids while displacing the opposing strand. This property, widely reported as a hallmark of PNA recognition of double-stranded DNA, is significantly less accessible to most other nucleic acid analogues.

Successful strategies for targeting G4-forming sequences with PNAs have been reported.^26–28^ Richter and co-workers developed naphthalene diimide ligand-PNA conjugates for selectively targeting a G4 in the HIV-1 long terminal repeat region.^14^ Cadoni *et al.* developed a red light-triggered chemical tool for the alkylation of G4 DNA structures, using a pro-reactive ligand incorporated into a PNA probe to specifically target the flanking region of the targeted secondary structure.^15^ In addition to oligonucleotide analogue-based approaches, alternative sequence-guided strategies have recently been explored. Notably, a G4 ligand has been conjugated to a duplex DNA-binding polyamide to recognize a hairpin stem formed within a long loop of a G4 in the hTERT promoter region, thereby enhancing G4 targeting.^29^ While conceptually related, as both approaches exploit recognition of sequences adjacent to G4s, this strategy relies on the presence of an additional specific structural feature. Moreover, polyamides interact with the minor groove of duplex DNA without relying on Watson–Crick base pairing. In contrast, PNA-based systems enable direct and programmable base-pairing with the target sequence and do not require the presence of pre-organized duplex elements, providing a more generally applicable strategy for positioning ligands relative to diverse G4 architectures.

Despite these advances, applying sequence-guided recognition to discriminate among closely spaced G4 units within the same genomic region remains largely unexplored. In particular, achieving directional control over ligand positioning to preferentially target a predetermined G4 among adjacent G4 motifs remains a distinct challenge, where spatial proximity imposes strict constraints on probe design and ligand positioning.

Among human genes that contain G4-forming sequences in the promoter near the transcription starting site (TSS), the proto-oncogene *c-KIT* is of great interest for drug design. This gene encodes a tyrosine kinase receptor, whose aberrant expression is associated with diseases like gastrointestinal stromal tumors, mast cell disease, and leukemia. The *c-KIT* promoter contains three adjacent G4-forming sequences – *c-KIT1*, c-KIT*, and *c-KIT2* – which are recognized transcriptional regulators and represent promising targets for anticancer drugs (Fig. 1A). The *c-KIT1* and *c-KIT2* sequences form parallel G4s, while c-KIT* folds in an antiparallel G4 with two G-tetrads (Fig. 1B).^30–32^ The three *c-KIT* G4s are separated by only a few nucleotides and may influence each other's stability and function. They have also been proposed to play distinct roles in gene regulation, either reducing or enhancing transcriptional output.^33^ Despite the enormous drug design efforts, no compounds have appeared to be highly selective for *c-KIT* promoter G4s compared to those occurring at other sites, nor do they discriminate among the three G4s. Furthermore, the possibility of selectively targeting individual or multiple adjacent G4 units within this clustered architecture has not been explored and remains an unmet challenge.

**Fig. 1:**
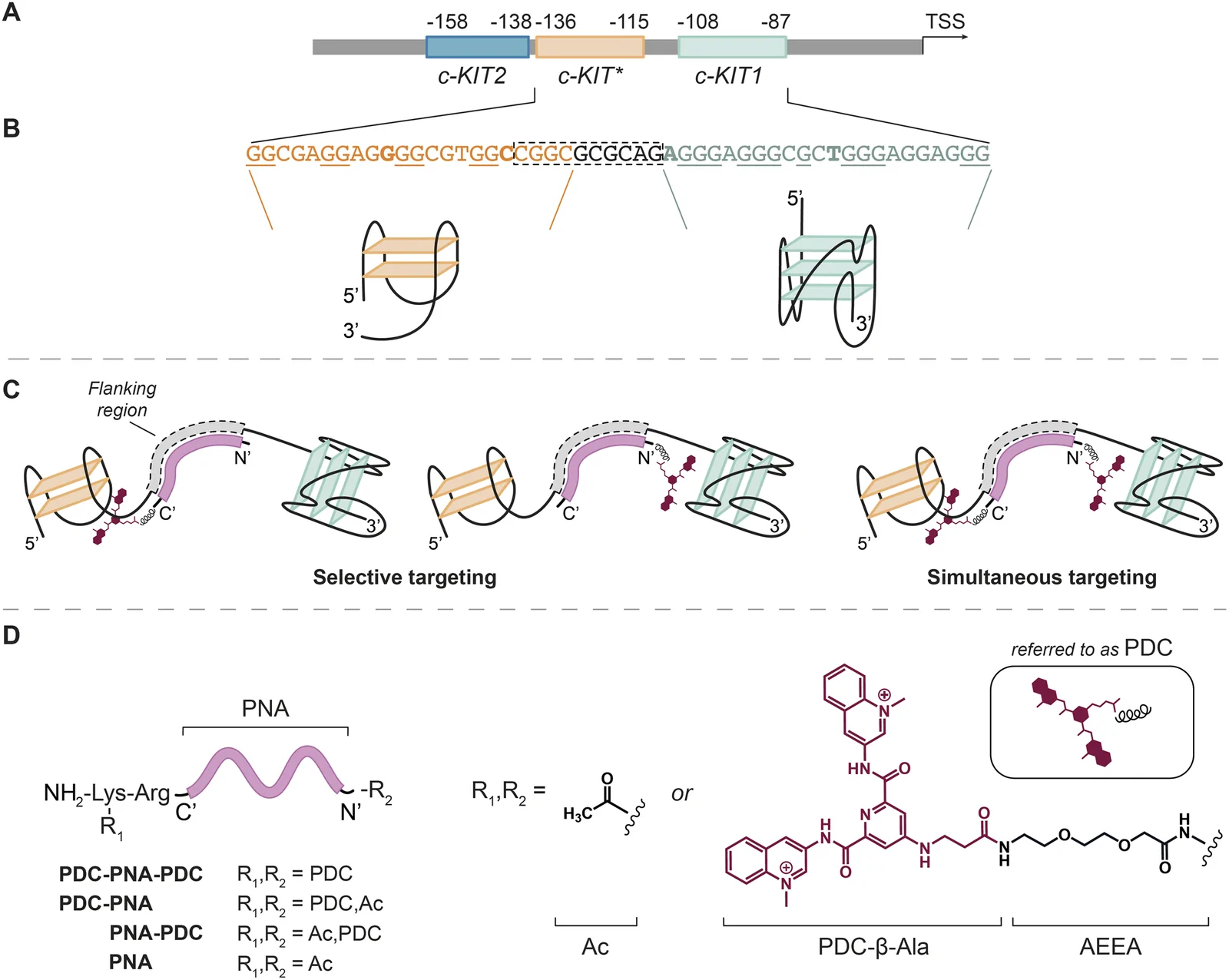
(A) Schematic representation of the G4-forming sequences (*c-KIT2*, c-KIT*, and *c-KIT1*) in the promoter of the *c-KIT* gene. The arrow indicates the transcription start site (TSS). (B) Primary sequence of the G-rich region upstream of the TSS between bases −87 and −136, along with schematic representations of the G4 structures adopted by c-KIT* and *c-KIT1* sequences. G4-forming sequences previously investigated in the literature are highlighted in orange and green. Underlined guanines are those involved in G-tetrad formation, while bold residues form Watson–Crick base pairs in the 3D structures of c-KIT* and *c-KIT1* G4s. The flanking region between the c-KIT* and *c-KIT1* G4s is enclosed within a dashed rectangle. (C) Schematic illustration of the strategy for specific targeting of *c-KIT* G4s through hybridization of PNA (depicted in pink) to the flanking region and (D) the design of G4 ligand-conjugated PNA probes.

In this study, we present a tool for the highly specific and spatially controlled targeting of *c-KIT* G4s based on the combined use of a PNA probe conjugated to a G4 ligand (Fig. 1C). To achieve selective targeting, the PNA moiety was designed to hybridize to the flanking region between the targeted G4 structures (*c-KIT1* and c-KIT*) without affecting their folding. The PDC ligand was conjugated to either the C- or N-terminus of the PNA to direct ligand binding toward a specific G4, enabling selective targeting (Fig. 1C and D). Importantly, this directional control allows discrimination between two closely spaced G4s within the same promoter region. In addition, conjugation of ligands to both ends of the PNA enables the simultaneous targeting of two adjacent *c-KIT* G4s. Overall, our results establish a successful strategy for the programmable and spatially defined targeting of one or two adjacent G4 structures within the same promoter, addressing a key limitation of current G4-targeting approaches.

## 2 Results and discussion

### 2.1 Target DNA and rational design of PNAs

The *c-KIT* promoter contains three distinct sites upstream of the TSS that are capable of folding into G4 structures: the region between bases −87 and −108, referred to as *c-KIT1*; the sequence delimited by bases −115 and −136, named c-KIT*; and the one comprised between bases −138 and −158, named *c-KIT2* (Fig. 1A). High-resolution NMR studies have provided detailed 3D structures for each isolated G4-forming sequence (PDB IDs: 2O3M for *c-KIT1*, 6GH0 for c-KIT*, and 2KYP for *c-KIT2*).^30–32^ Examining the positioning of these G4 units within the *c-KIT* promoter sequence, we note that *c-KIT2* and c-KIT* are separated by only one nucleotide, excluding the design of a PNA capable of targeting the region between these two G4s. On the other hand, c-KIT* and *c-KIT1* are separated by ten nucleotides, not counting residues engaged in additional base pairs within the individual G4 structures (highlighted in bold in Fig. 1B and Table 1). This provides the opportunity to design PNAs that can potentially hybridize to that region. Based on this, we focused our study on the *c-KIT* promoter region containing c-KIT* and *c-KIT1* G4s. Therefore, we synthesized and targeted a 50-nucleotide long sequence encompassing both G4 motifs, hereafter referred to as *c-KIT*_*50*_ (Table 1). For comparison, we also synthesized individual c-KIT* and *c-KIT1* sequences with 10-nucleotide flanking regions at the 3′-end and 5′-end, respectively (named 
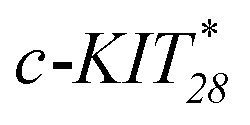
 and *c-KIT1*_*32*_). Additionally, the isolated c-KIT* and *c-KIT1* G4-forming sequences were used as controls.

**Table 1:** *c-KIT*-derived DNA sequences used in this study

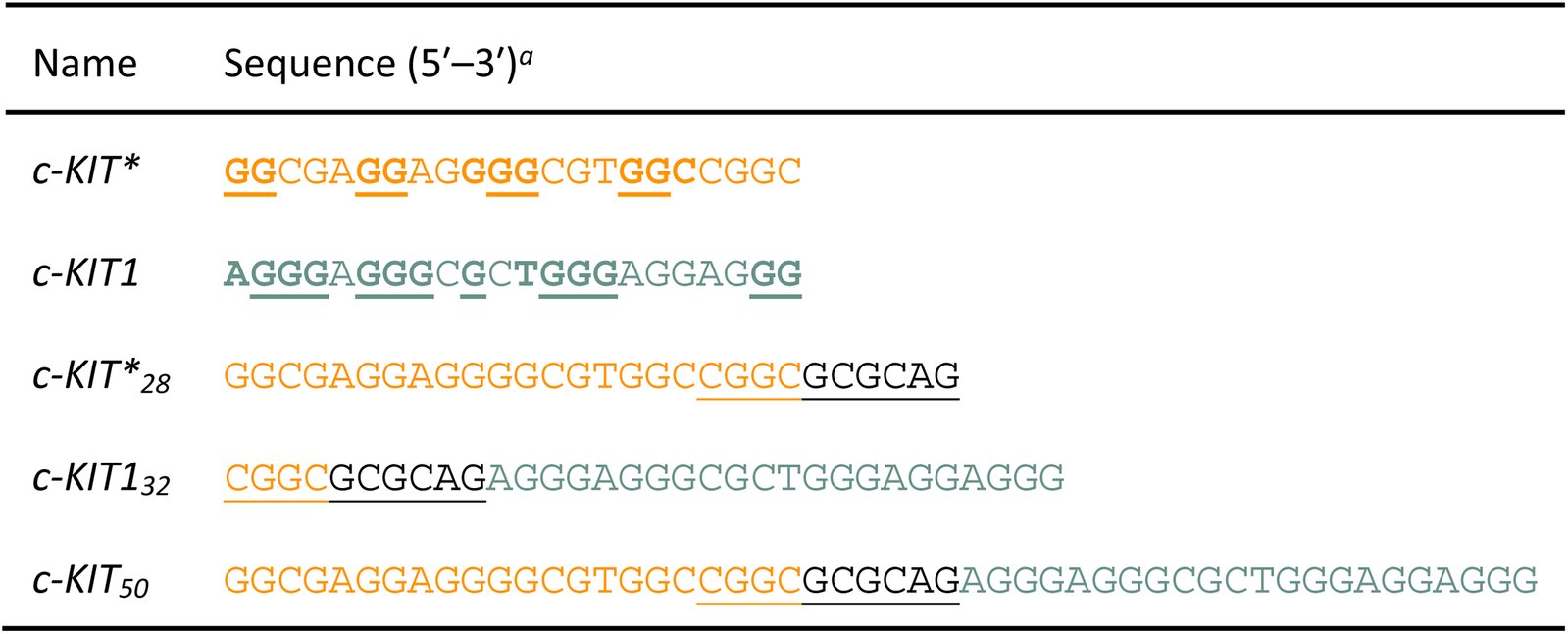

a Bold and underlined guanines are those belonging to the G-tetrads, while bold residues form Watson–Crick base pairs in the 3D structure of c-KIT* and *c-KIT1* G4s. The residues underlined are those used as targets of PNAs.

Considering that PNA length is a key factor for specificity and PNA : DNA duplex stability, and that the length and sequence of a PNA can be varied for optimization, we designed and synthesized a set of PNAs (PNA_10_, PNA_9_, PNA_8_, and PNA_6_, Table S1) complementary to the region between the c-KIT* and *c-KIT1* G4 motifs.

To explore the impact of PNA length, we first designed a PNA potentially able to fully hybridize with all ten complementary nucleotides in the flanking sequence (PNA_10_). To reduce constraints arising from contextual hybridization and stacking interactions, we also developed shorter variants (PNA_9_ and PNA_8_) that exclude one or two nucleotides in the flanking region closest to the G4s from hybridization. Additionally, we designed a shorter PNA (PNA_6_) that should hybridize exclusively with the flanking tract not belonging to either of the two isolated c-KIT* and *c-KIT1* G4-forming sequences. All PNAs incorporated an arginine to increase water solubility and a lysine residue (with an orthogonal Dde-protecting group) to allow further conjugation and ligand incorporation at the C-terminus *via* an amide bond.

### 2.2 G4 folding behavior and thermal stability of *c-KIT*_*50*_

Previous work has shown that neighboring G4-forming domains within the *c-KIT* promoter are structurally interdependent when embedded in longer sequences.^33^ Here, we specifically characterized *c-KIT*_*50*_, the construct selected for PNA targeting, by circular dichroism (CD), thermal difference spectrum (TDS), and 1D ^1^H NMR experiments to assess its folding behavior under the experimental conditions used in this study. To identify suitable experimental conditions for the comparative biophysical analyses, *c-KIT*_*50*_ was initially examined in 10 mM phosphate buffer containing 100 mM KCl and in 5 mM phosphate buffer containing 20 mM KCl (pH 7.0). CD spectra recorded under both conditions showed similar profiles, with a major positive band at about 265 nm, a shoulder at 290 nm, and a negative band at 240 nm (Fig. S1A), while the overall CD intensity was significantly higher under low ionic strength. This CD spectral profile suggested the formation of a predominant parallel G4 structure and one or more additional conformations. The corresponding TDS spectra further supported G4 formation in the buffer containing 20 mM KCl, where the typical G4 pattern was observed, with positive maxima at 273 and 255 nm, a shoulder at 243 nm, and a negative minimum around 295 nm, whereas a slightly altered profile was observed under high ionic strength conditions (Fig. S1B).^34^ Consistently, ^1^H NMR spectra of *c-KIT*_*50*_ showed that, under both conditions, in addition to Hoogsteen imino signals diagnostic of G4s (10.5–12.5 ppm), broad Watson–Crick imino signals were present between 12.5 and 13.6 ppm (Fig. S1C). Notably, the NMR spectrum recorded at 20 mM KCl displayed sharper proton resonances than that obtained at 100 mM KCl, indicating lower spectral heterogeneity under the former condition. The 20 mM KCl condition was therefore selected for the detailed comparative analyses because it retained diagnostic G4 features and provided better spectral resolution.

Detailed characterization of *c-KIT*_*50*_ in comparison with the shorter c-KIT* and *c-KIT1* G4-forming sequences was therefore carried out using CD and ^1^H NMR experiments. Consistent with previous reports, the CD spectra of c-KIT* and c-*KIT1* in the presence of K^+^ revealed distinct topologies: c-KIT* showed a minimum at 263 nm and a maximum at ∼290 nm, characteristic of an antiparallel G4,^31^ whereas *c-KIT1* displayed a positive band at ∼264 nm and a negative band near 240 nm, typical of a parallel G4 (Fig. 2A). Notably, the experimental CD spectrum of *c-KIT*_*50*_ closely resembled the spectrum resulting from the arithmetic sum of the individual spectra of c-KIT* and *c-KIT1* acquired under the same experimental conditions (Fig. 2A). However, the *c-KIT*_*50*_ spectrum exhibited a lower intensity at 290 nm compared to the sum spectrum, as well as a slight shift of the main maximum from 264 nm to 266 nm. These differences may reflect a reduced formation of the c-KIT* G4 within the longer sequence and/or some differences in the structures formed by *c-KIT*_*50*_ compared with those formed by the shorter sequences.

**Fig. 2:**
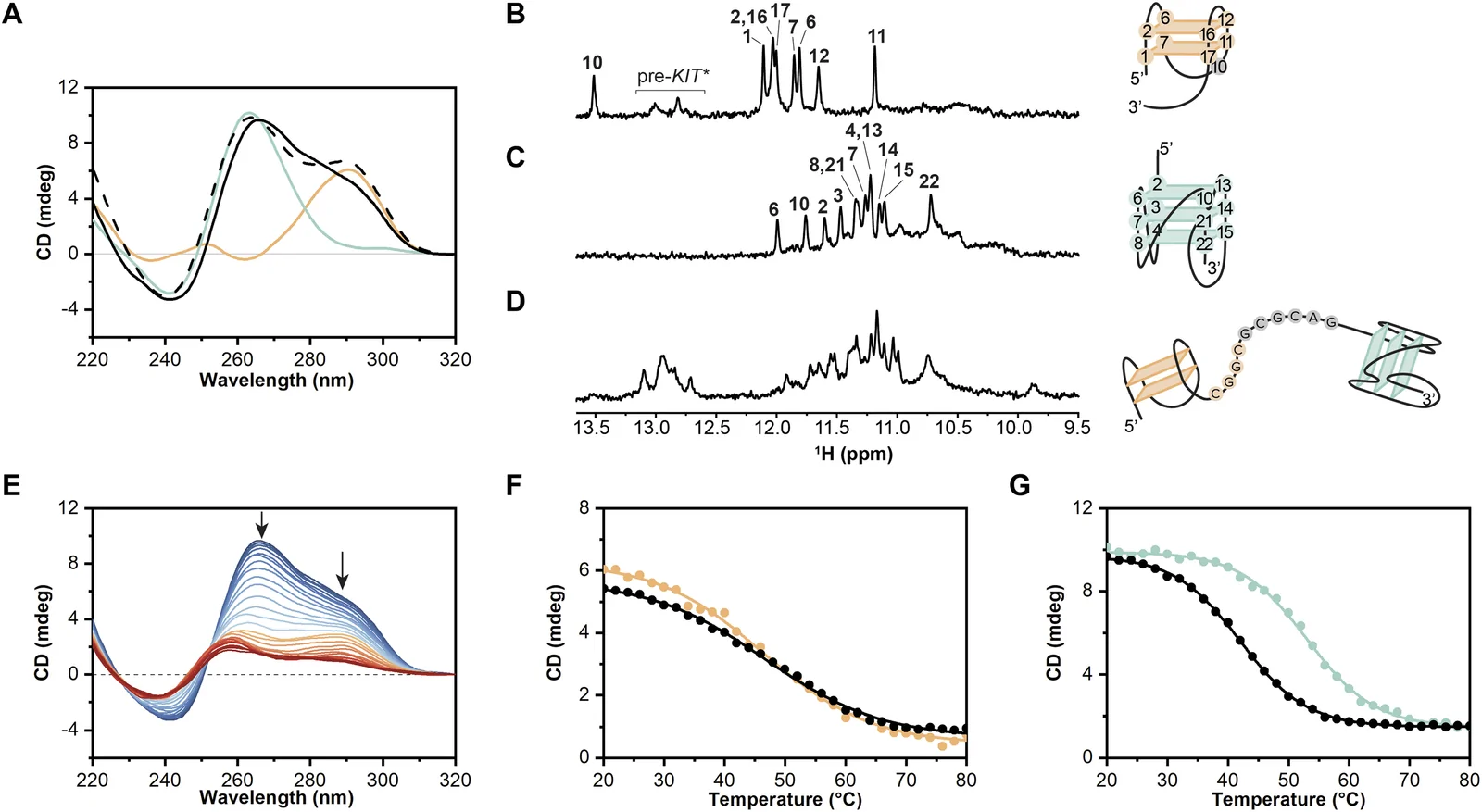
(A) CD spectra of c-KIT*, *c-KIT1*, and *c-KIT*_*50*_ (orange, green, and black solid lines, respectively), along with the arithmetic sum of c-KIT* and *c-KIT1* spectra (dashed black line) at 20 °C. (B–D) Imino region of ^1^H NMR spectra of (B) c-KIT*, (C) *c-KIT1*, and (D) *c-KIT*_*50*_ (0.05 mM) at 25 °C. Schematic structures to the right of the spectra are for illustrative purposes only. (E) Temperature-dependent CD spectra of *c-KIT*_*50*_ from 20 °C to 80 °C (color-coded from blue to red). (F and G) CD melting curves of (F) *c-KIT*_*50*_ and c-KIT* monitored at 290 nm (black and orange circles, respectively) and (G) of *c-KIT*_*50*_ and *c-KIT1* monitored at 264 nm (black and green circles, respectively). Fitting of the melting profiles were reported as solid lines. All spectra were recorded in 5 mM phosphate buffer with 20 mM KCl (pH 7.0). NMR samples required H_2_O/D_2_O (90 : 10).

The ^1^H NMR spectra of c-KIT* and *c-KIT1* confirmed their folding into single intramolecular G4 structures in the buffer containing 20 mM KCl. c-KIT* displayed narrow and well-resolved imino peaks between 11.0 and 12.2 ppm, consistent with two G-quartets, along with a peak at 13.5 ppm indicative of the presence of an additional Watson–Crick GC base pair (Fig. 2B).^31^ Two broad signals of lower intensity were also observed between 12.6 and 13.3 ppm, indicative of the presence of a small amount of a pre-folded species, known as pre-KIT*.^31^ In contrast, *c-KIT1* exhibited well-resolved resonances consistent with the formation of a G4 with three G-quartets (Fig. 2C).^30^ The ^1^H NMR spectrum of *c-KIT*_*50*_ showed broader peaks in the imino proton region (Fig. 2D). Two groups of signals were clearly observed: the Hoogsteen signals (10.5–12.2 ppm) do not exclude the coexistence of c-KIT* and *c-KIT1* G4s within the longer sequence, while the Watson–Crick base pair signals (12.5–13.6 ppm) suggest also the presence of alternative intramolecular interactions and/or intermolecular partially paired homo-dimeric structure(s). Collectively, these data support G4 formation within *c-KIT*_*50*_ and are consistent with contributions from both the c-KIT* and *c-KIT1* G4-forming regions, although partially unfolded and/or minor alternative conformations may also be present, particularly in the region corresponding to c-KIT*.

The thermal stability of the G4-forming sequences was then evaluated by monitoring CD and ^1^H NMR spectra as a function of temperature (Fig. 2E, S2 and S3). The CD melting curves of the individual G4s, determined by monitoring CD signal changes at 290 nm for c-KIT* and 264 nm for *c-KIT1* (Fig. 2F and G), revealed apparent melting temperatures (*T*_½_) of 46.4 (±0.3) and 53.1 (±0.2) °C, respectively, indicating the lower stability of c-KIT* (Table S2). These results were corroborated by the corresponding temperature-dependent ^1^H NMR spectra (Fig. S3). Monitoring the CD signal of *c-KIT*_*50*_ at 290 and 264 nm, which can be reasonably attributed primarily to c-KIT* and *c-KIT1* G4s, respectively, revealed two distinct melting profiles (Fig. 2F and G), with *T*_½_ values of 46.7 (±0.4) and 42.1 (±0.1) °C for the first and the second wavelength, respectively. These results suggest that the G4 formed within the *c-KIT1* region of *c-KIT*_*50*_ was less stable than the corresponding G4 formed by the shorter sequence. The temperature-dependent ^1^H NMR spectra of *c-KIT*_*50*_ supported the CD melting data, showing the complete disappearance of imino resonances above 50 °C (Fig. S3). Together, these data provided insight into the structural and thermal behavior of *c-KIT*_*50*_ needed to evaluate the effects of PNA hybridization and PNA-directed ligand stabilization.

### 2.3 Evaluation of PNA binding to *c-KIT*_*50*_ identified PNA_8_ as the most promising candidate

To explore whether the designed PNAs were able to hybridize to the *c-KIT*_*50*_ sequence in the region between the two G-rich motifs without interfering with the possible G4 folding, a series of experiments were carried out in the presence of each PNA to evaluate their effect on *c-KIT*_*50*_. CD spectra revealed that the presence of one molar equivalent of the shorter PNAs, *i.e.* PNA_6_ and PNA_8_, did not change the overall spectral profile of *c-KIT*_*50*_, indicating only minimal effects on DNA conformation (Fig. 3A). In contrast, PNA_9_ and PNA_10_ significantly altered the CD spectrum upon their addition. In particular, they induced the disappearance of the band at 290 nm, suggesting that their binding interferes with the formation of a G4 structure by the c-KIT* region of *c-KIT*_*50*_. All PNAs, although to different extents, increased the intensity of the band at 264 nm. This intensity variation is consistent with the formation of a PNA–DNA heteroduplex whose signal overlaps with that of the parallel G4 contribution (Fig. S4).^22^ In addition, ^1^H NMR spectra showed only a slight broadening of the imino proton signals upon binding of PNA_8_ and PNA_9_, whereas PNA_6_ and PNA_10_ caused a more pronounced broadening of such signals (Fig. 3B). The appearance of additional Watson–Crick base-pair imino signals (12.4–13.3 ppm) further supports the formation of PNA–DNA complexes.

**Fig. 3:**
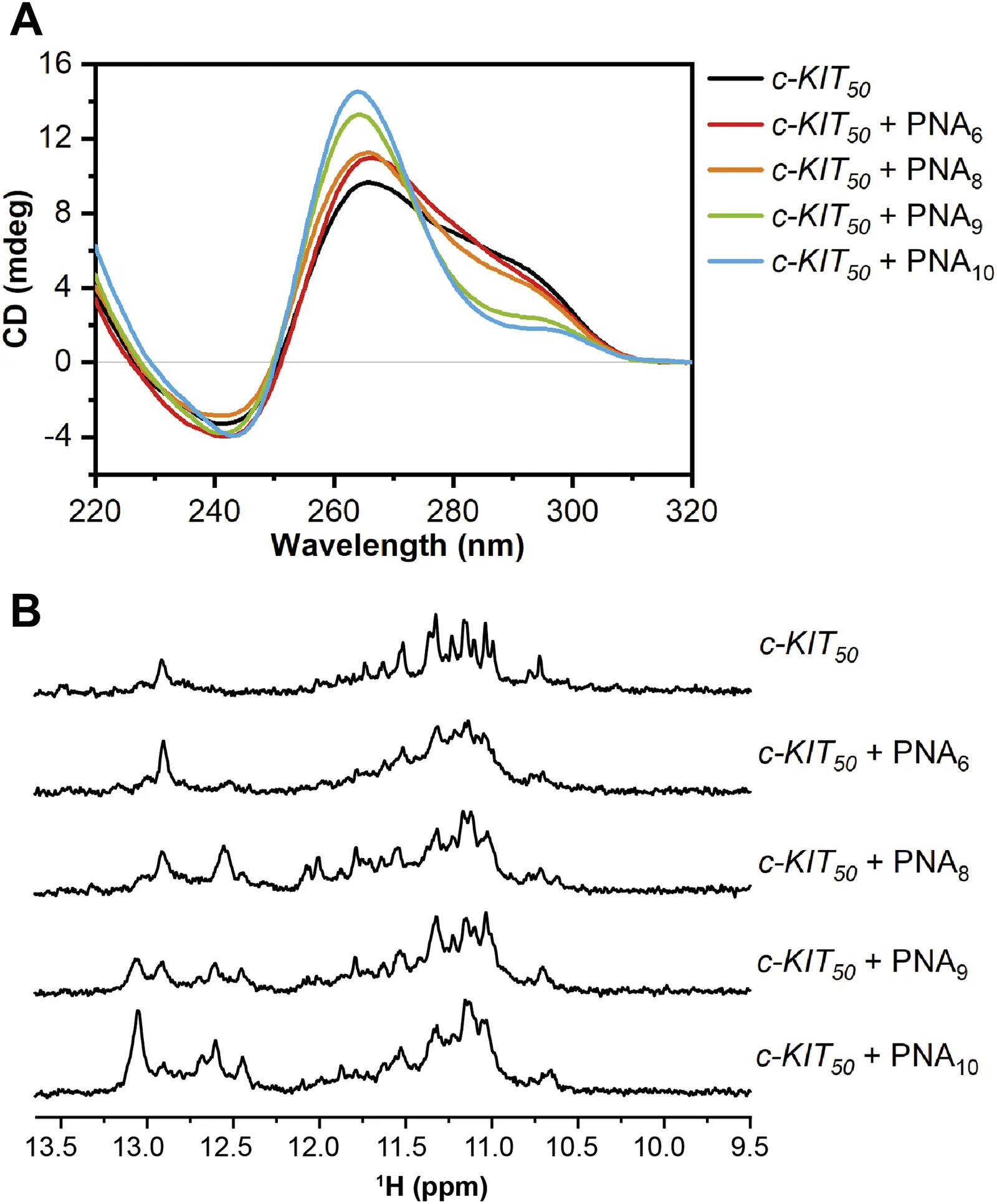
(A) CD spectra of *c-KIT*_*50*_ (1.5 µM) in the absence and presence of one equivalent of PNA_6_, PNA_8_, PNA_9_, and PNA_10_ at 20 °C. (B) Imino region of ^1^H NMR spectra of *c-KIT*_*50*_ (0.05 mM) in the absence and presence of one equivalent of each PNA at 37 °C. Experiments were performed in 5 mM phosphate buffer with 20 mM KCl (pH 7.0). NMR samples required H_2_O/D_2_O (90 : 10).

Temperature-dependent CD spectra were also acquired for each complex (Fig. S5). Analysis of the CD spectra and melting profiles at 290 nm revealed that, while PNA_9_ and PNA_10_ abolished this band, PNA_6_ and PNA_8_ showed only minimal effects. On the other hand, CD melting analysis at 264 nm indicated that none of the PNAs negatively affected the parallel G4 within *c-KIT*_*50*_; rather, PNA_8_, PNA_9_, and PNA_10_ appeared to enhance its stability (Table S2). Consistently, temperature-dependent ^1^H NMR spectra of *c-KIT*_*50*_/PNA complexes showed an overall stabilization of the Hoogsteen-type imino proton signals (Fig. S6).

Competition between PNA–DNA heteroduplex and PNA–PNA homoduplex formation was also assessed. All PNAs exhibited self-complementary base pairing, as indicated by Watson–Crick imino signals in NMR spectra (Fig. S7). At 25 °C, a prominent sharp peak around 12.65 ppm was consistently observed, likely resulting from intermolecular hydrogen bonding within the “CGCG” tract.^35^ With increasing PNA length, additional peaks appeared, indicating the involvement of more interactions, along with a corresponding increase in PNA–PNA homoduplex stability. Upon incubation of *c-KIT*_*50*_ with PNAs, DNA–PNA heteroduplex formation altered the signals in the Watson–Crick region. For interactions with PNA_6_ and PNA_8_, a peak around 12.55 ppm appeared, consistent with the formation of heteroduplexes whose stability was comparable to that of homoduplexes. Conversely, PNA_9_ and PNA_10_ resulted in the appearance of two signals between 12.5 and 12.6 ppm, suggesting coexistence of homo- and heteroduplexes. Notably, the *c-KIT*_*50*_/PNA_9_ heteroduplex appeared to be less stable than the PNA_9_ homoduplex, whereas the *c-KIT*_*50*_/PNA_10_ complex exhibited stability similar to its homoduplex counterpart.

Collectively, these results identified PNA_8_ as the most promising candidate, as it hybridizes to the target, forming a sufficiently stable complex without altering the conformation and stability of the structures formed within *c-KIT*_*50*_. This feature is particularly critical in the context of adjacent G4-forming motifs, where even minor perturbations could influence neighboring structures. The ability of the PNA probe to hybridize without disrupting G4 folding is therefore a key prerequisite for the selective targeting of individual G4 units within multi-G4 architectures.

### 2.4 PDC-β-Ala nonspecifically stabilizes the c-KIT* and *c-KIT1* G4 motifs

With the aim of achieving highly specific targeting of *c-KIT* G4s through the combined use of a PNA probe conjugated to a validated G4 ligand, we selected PDC, a well-established bisquinolinium 2,6-pyridinedicarboxamide ligand extensively used as a reference G4 binder because of its strong affinity for G4 structures and selectivity over duplex DNA, and previously employed in both non-covalent and covalent G4-targeting strategies.^36–39^ At the same time, PDC recognizes a broad range of G4 structures with limited discrimination among individual G4 targets, making this scaffold particularly suitable for testing whether sequence-specific PNA hybridization can confer positional control over ligand binding. A β-alanine-modified derivative (PDC-β-Ala) was synthesized to enable PNA coupling (Scheme S1).^15^ CD and ^1^H NMR experiments were first used to assess PDC-β-Ala binding to isolated c-KIT* and *c-KIT1* G4s. CD melting analysis is widely used to evaluate G4 stabilization, as ligand binding typically shifts the unfolding transition to higher temperatures, providing a direct measure of how ligand binding affects structural stability. For c-KIT*, the ligand increased CD signal intensity at 20 °C (Fig. S8A and S8B) and significantly enhanced its thermal stability (Δ*T*_½_ = 15.9 ± 0.7 °C) (Fig. 4A and Table S2). The ^1^H NMR spectra showed two distinct families of signals in the imino proton region, consistent with the coexistence of bound and free G4 and thus indicating moderate binding affinity but clear ligand-induced stabilization of the DNA structure (Fig. S8C). Similarly, *c-KIT1* G4 showed enhanced thermal stability in the presence of PDC-β-Ala (Δ*T*_½_ = 11.4 ± 0.6 °C) (Fig. 4A and Table S2), with broadening of the imino proton signals indicative of ligand interaction (Fig. S9).

**Fig. 4:**
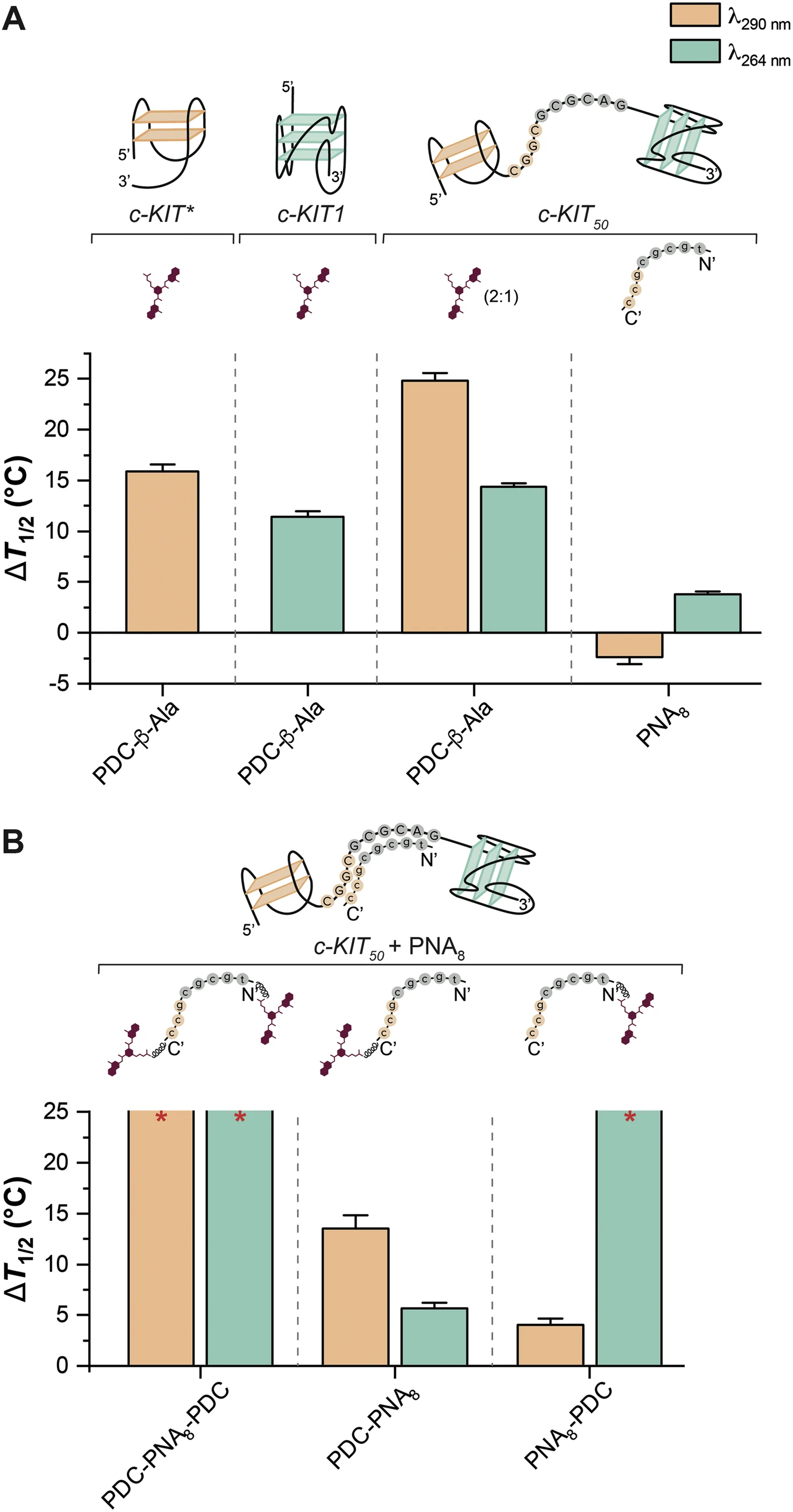
Bar graphs depicting (A) ligand-induced changes in melting temperature (Δ*T*_½_) for the indicated G4-forming sequences and (B) Δ*T*_½_ values for *c-KIT*_*50*_ in the presence of PNA-ligand conjugates relative to PNA_8_ alone. Data were obtained from CD melting experiments. Red asterisks denote cases in which Δ*T*_½_ values could not be determined due to stabilization beyond the measurable temperature range. Schematic structures are shown for illustrative purposes only.

For the longer *c-KIT*_*50*_ sequence, CD spectra of the DNA/ligand mixture (1 : 2 molar ratio) were recorded as a function of temperature, revealing different temperature-dependent changes in the intensities of the 264 and 290 nm bands, indicative of differential stabilization of the two G4-forming domains (Fig. S10A–C). CD melting analysis confirmed the ligand-induced stabilization of the G4s formed within *c-KIT*_*50*_, with Δ*T*_½_ values of 14.4 (±0.3) and 24.8 (±0.8) °C at 264 and 290 nm, respectively (Fig. 4A and Table S2). Notably, while the Δ*T*_½_ at 264 nm was comparable to that of the isolated *c-KIT1* G4, the antiparallel G4 within *c-KIT*_*50*_ displayed markedly higher sensitivity to ligand binding than c-KIT*. NMR spectra further revealed signal broadening consistent with DNA polymorphism, yet temperature-dependent analysis confirmed the significant ligand-induced stabilization (Fig. S10D).

### 2.5 Dual recognition strategy reveals PNA-directed G4 targeting within *c-KIT*_*50*_

To combine hybridization to the flanking region between c-KIT* and *c-KIT1* motifs with direct targeting of the G4 structures, PNA_8_ was conjugated to PDC-β-Ala. A flexible AEEA spacer was also incorporated to minimize steric constraints between the PNA–DNA heteroduplex and the ligand, allowing the PDC moiety to effectively engage the G4 structure (Fig. 1C and D). Three derivatives were synthesized: (i) PNA_8_–PDC (with PDC at the N-terminus), (ii) PDC–PNA_8_ (bearing PDC at the C-terminus), and (iii) PDC–PNA_8_–PDC (featuring PDC conjugation at both termini), designed to specifically target *c-KIT1*, c-KIT*, or both G4s simultaneously.

To evaluate the interaction of these PDC-conjugated PNAs with c-KIT* and *c-KIT1* G4s within *c-KIT*_*50*_, temperature-dependent CD spectra were recorded (Fig. S11). While unmodified PNA_8_ had negligible effects (Fig. 4A and S5B), PDC–PNA_8_–PDC produced a dramatic stabilization, with the CD signals at both 264 and 290 nm remaining clearly observable even at 80 °C (Fig. S11A). To dissect G4 motif-specific effects, *c-KIT*_*50*_ was incubated separately with PDC–PNA_8_ and PNA_8_–PDC, and the resulting *T*_½_ values were evaluated relative to PNA_8_ alone. In the first case, a Δ*T*_½_ value of 13.5 ± 1.2 °C was determined at 290 nm (Fig. 4B, S11B and Table S2), while a smaller but detectable stabilization was also observed at 264 nm (Δ*T*_½_ = 5.4 ± 0.7 °C), suggesting that PDC–PNA_8_ might exert a secondary effect on the *c-KIT1* G4-forming region. By contrast, incubation of *c-KIT*_*50*_ with PNA_8_–PDC (targeting *c-KIT1* region) led to an undeterminable Δ*T*_½_ value at 264 nm, consistent with very strong stabilization of the *c-KIT1* G4 and comparable to that observed for PDC–PNA_8_–PDC, while only slight effects on c-KIT* G4 within *c-KIT*_*50*_ were detected, as indicated by the small Δ*T*_½_ at 290 nm (4.0 ± 0.5 °C) (Fig. 4B, S11C and Table S2).

Temperature-dependent ^1^H NMR spectra confirmed these trends, with all conjugates retaining DNA imino proton peaks above 70 °C, indicating strong stabilization. While the PDC–PNA_8_–PDC/*c-KIT*_*50*_ complex exhibited broad imino proton peaks (Fig. S12), incubation of *c-KIT*_*50*_ with either PDC–PNA_8_ or PNA_8_–PDC led to sharper imino proton peaks in the downfield or upfield regions, respectively (Fig. 5A and B). Notably, the PDC–PNA_8_/*c-KIT*_*50*_ complex showed imino signals between 11.5 and 12.2 ppm closely resembling those of the PDC-β-Ala/c-KIT* G4 complex (Fig. 5C), suggesting that PNA hybridization effectively directs PDC binding to c-KIT* within *c-KIT*_*50*_. Similarly, incubation of *c-KIT*_*50*_ with PNA_8_–PDC generated a distinct set of signals between 10.5 and 11.6 ppm, closely matching those observed in the PDC-β-Ala/*c-KIT1* G4 complex (Fig. 5C), indicating that in this case the PNA successfully directs PDC interaction toward the *c-KIT1* G4 within *c-KIT*_*50*_.

**Fig. 5:**
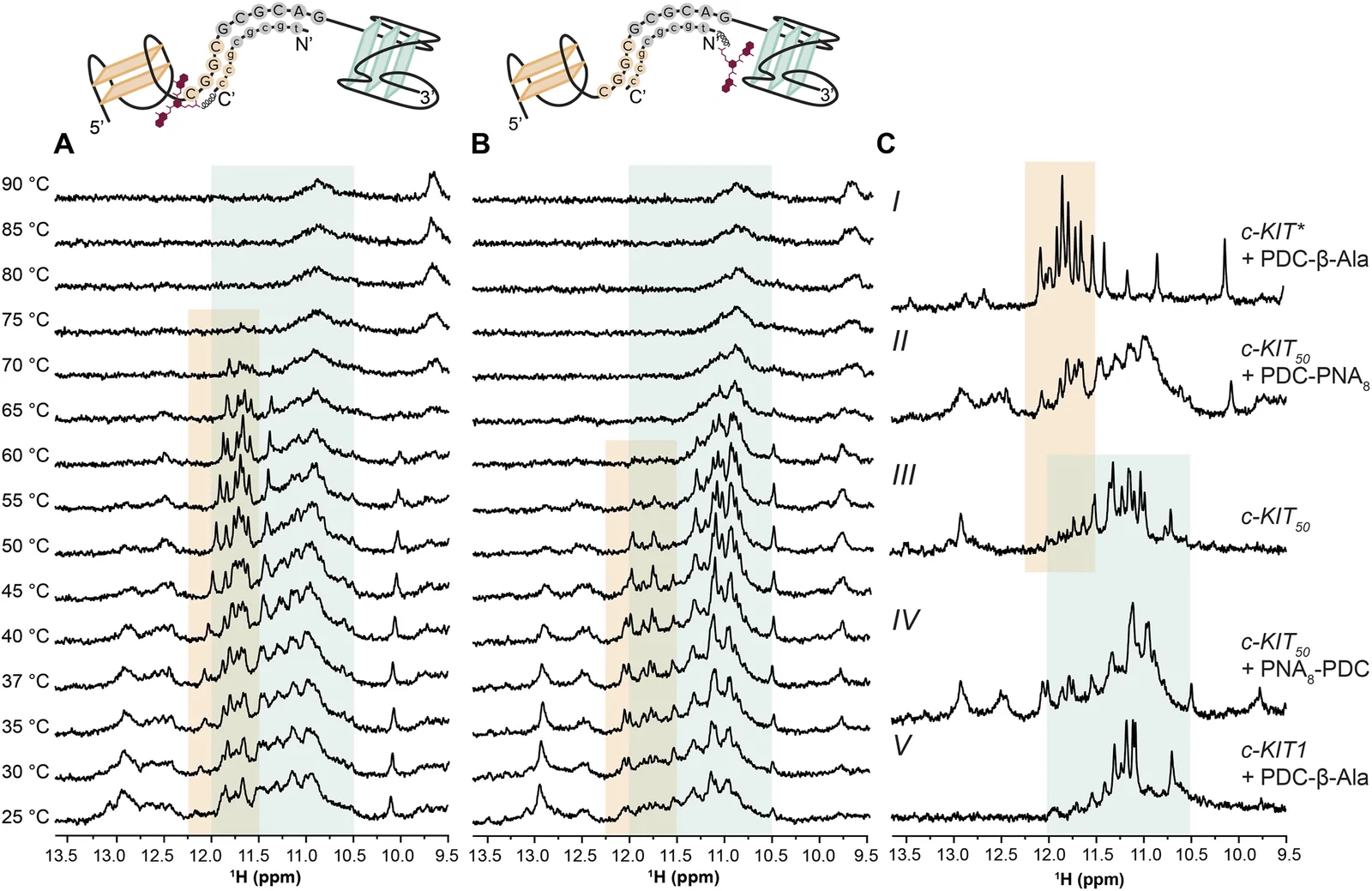
(A and B) Imino region of 1D ^1^H NMR spectra acquired at various temperatures for *c-KIT*_*50*_ (0.05 mM) in the presence of one equivalent of (A) PDC–PNA_8_ and (B) PNA_8_–PDC. Schematic structures above the spectra are for illustrative purposes only. (C) Imino region of ^1^H NMR spectra recorded at 37 °C for (I) c-KIT* in the presence of PDC-β-Ala (1 : 1), (II) *c-KIT*_*50*_ in the presence of PDC–PNA_8_ (1 : 1), (III) *c-KIT*_*50*_ alone, (IV) *c-KIT*_*50*_ in the presence of PNA_8_–PDC (1 : 1), and (V) *c-KIT1* in the presence of PDC-β-Ala (1 : 1). DNA concentration: 0.05 mM. All spectra were acquired in 5 mM phosphate buffer with 20 mM KCl, pH 7.0 (H_2_O/D_2_O, 90 : 10).

To complement the CD and NMR evidence with a quantitative measure of binding affinity, fluorescence-quenching titrations were performed using 5′-Cy5.5-labelled *c-KIT*_*50*_ and PDC–PNA_8_.^40,41^ Increasing concentrations of PDC–PNA_8_ produced a concentration-dependent quenching of Cy5.5 fluorescence emission, yielding a dissociation constant (*K*_d_) of 57 ± 12 nM (Fig. S13). This value is lower than the *K*_d_ values reported for the unconjugated PDC ligand toward individual G4 structures, which typically fall in the submicromolar-to-micromolar range, depending on the G4 target and experimental conditions.^11,42,43^ Although these literature values are not directly comparable, the nanomolar *K*_d_ is consistent with a favorable contribution of PNA-mediated anchoring to target engagement by positioning the PDC moiety in proximity to the intended G4.

To support the key role of PNA in directing ligand binding to a specific G4, we synthesized and investigated three additional derivatives in which PDC was conjugated to a control PNA (CTRL) lacking complementarity to the target sequence. In analogy to what was done with PNA_8_, CTRL PNA was conjugated to PDC-β-Ala at the N-terminus (CTRL–PDC), C-terminus (PDC–CTRL), or at both termini (PDC–CTRL–PDC) (Table S1). The binding of these conjugates to *c-KIT*_*50*_ G4s was examined by CD and NMR experiments performed under the same conditions (Fig. S14 and S15). Temperature-dependent CD and ^1^H NMR spectra of *c-KIT*_*50*_ in the presence of these molecules revealed that, although some stabilization of the G4s occurred (particularly the parallel G4), this effect was essentially ligand-driven, as all three derivatives produced essentially the same effect regardless of the conjugation site (Table S2). In contrast, in the case of PNA_8_ the effect clearly depended on the position of the conjugation, highlighting the directing role of the PNA probe.

Together, these results show that while PDC-β-Ala alone behaves as a largely unspecific G4 stabilizer, its conjugation with a sequence-complementary PNA converts it into a more discriminating probe. In particular, the position of ligand conjugation dictates the preferential stabilization of a specific G4 motif within *c-KIT*_*50*_, demonstrating the key role of PNA hybridization in directing ligand binding. Notably, such directional control cannot be achieved with conventional, non-conjugated G4 ligands, which lack positional constraints and typically interact with multiple G4 structures without discrimination. Even within a scaffold containing two adjacent G4 motifs, PNA hybridization defines the spatial positioning of the ligand, enabling motif-specific stabilization. Moreover, the observed simultaneous stabilization of both G4 motifs by PDC–PNA_8_–PDC underscores the modularity of the PNA-ligand system, indicating that it can be extended from the selective targeting of individual G4s to the concurrent stabilization of adjacent structures within the same DNA region. The ability to bias ligand interaction toward a specific G4 within a closely spaced pair represents a key conceptual advance over previously reported sequence-guided systems, which have primarily focused on enhancing affinity rather than controlling positional selectivity. Overall, these findings establish PNA-guided conjugation as a powerful strategy to impose selectivity on otherwise promiscuous G4 ligands.

### 2.6 PNA-guided ligand positioning drives more effective G4 binding

To strengthen the evidence that suitable PNA_8_ conjugation directs PDC to selectively engage c-KIT* and *c-KIT1* G4s within the *c-KIT*_*50*_ sequence, two additional potential G4-forming sequences were analyzed: 
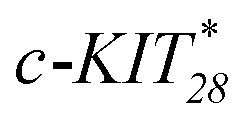
 and *c-KIT1*_*32*_ (Table 1). The former corresponds to the c-KIT* 22-mer extended by six nucleotides at the 3′-end, while the latter to the *c-KIT1* 22-mer extended by ten nucleotides at the 5′-end, additions that recreate the shared flanking region present in *c-KIT*_*50*_ while isolating each G4 motif.

A non-denaturing PAGE analysis was first performed to assess their folding behavior, with c-KIT*, *c-KIT1*, and *c-KIT*_*50*_ used as controls (Fig. S16). As expected, the additional flanking nucleotides generally reduced electrophoretic mobility. Interestingly, 
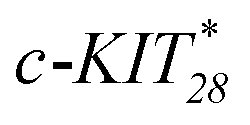
 migrated similarly to *c-KIT1*, suggesting the formation of a secondary structure that compensates for the presence of additional nucleotides, whereas *c-KIT1*_*32*_ showed slower migration. CD analysis further showed that 
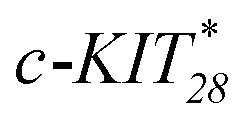
 retained an antiparallel G4 signature (Fig. S17A). Its melting temperature (*T*_½_ = 52.8 ± 0.5 °C) was slightly higher than that of the isolated c-KIT* G4 and the corresponding transition of *c-KIT*_*50*_ at 290 nm (*T*_½_ of 46.4 and 46.7 °C, respectively) (Table S2). This suggests that the flanking nucleotides in 
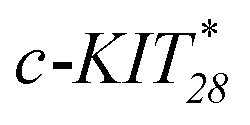
 may contribute to stabilizing the structure. By contrast, *c-KIT1*_*32*_ did not display a CD profile or a measurable melting transition consistent with a well-defined G4 (Fig. S17B), suggesting that the 5′-flanking nucleotides favor a heterogeneous ensemble of alternative secondary structures and/or unfolded species rather than the G4 structure adopted by the *c-KIT1* sequence.

We next examined how PNA_8_ binding and PDC conjugation affected the thermal stability of these two constructs. For 
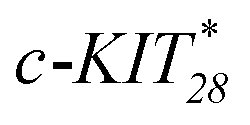
, addition of PNA_8_ only slightly affected G4 stability, with a *T*_½_ of 50.2 ± 0.4 °C (Fig. 6A and Table S2). In contrast, directing PDC to the same target through PDC–PNA_8_ produced substantial stabilization without significant changes in the CD spectral profile, increasing the *T*_½_ to 67.0 ± 0.2 °C (Δ*T*_½_ = 14.2 ± 0.7 °C relative to 
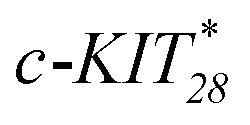
 alone; Fig. 6B and Table S2). The magnitude of this stabilization was comparable to that induced by PDC-β-Ala on the isolated c-KIT* G4 (Δ*T*_½_ = 15.9 ± 0.7 °C).

**Fig. 6:**
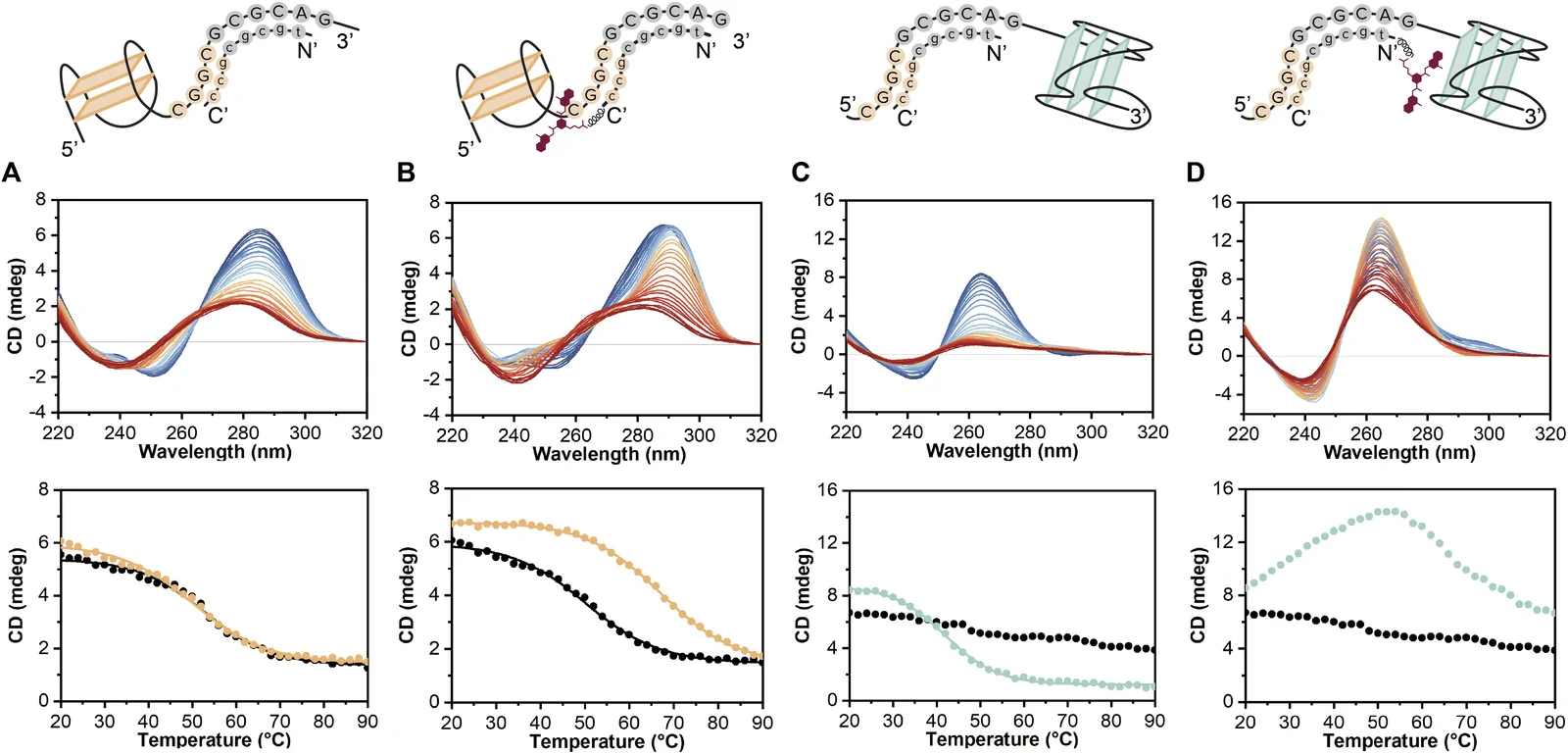
CD experiments for (A and B) 
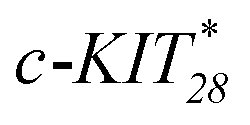
 and (C and D) *c-KIT1*_*32*_ in the presence of one equivalent of (A) PNA_8_, (B) PDC–PNA_8_, (C) PNA_8_, and (D) PNA_8_–PDC. The top panels show CD spectra from 20 to 90 °C, color-coded from blue to red. The bottom panels show CD melting curves at (A and B) 290 nm and (C and D) 264 nm. For comparison, the melting curve of the corresponding DNA is shown in black, alongside those recorded in the presence of PNAs (colored dots). Fitted curves are shown as solid lines. Spectra were recorded in 5 mM phosphate buffer with 20 mM KCl (pH 7.0). Schematic structures above the spectra are for illustrative purposes only.

In the case of *c-KIT1*_*32*_, PNA_8_ induced the characteristic CD profile of a parallel G4 (Fig. 6C) and a measurable *T*_½_ of 42.0 ± 0.2 °C, closely matching the transition observed for *c-KIT*_*50*_ at 264 nm (Table S2). This suggests that PNA hybridization to the flanking region promotes folding of the *c-KIT1* region into G4. Conjugation of PDC to PNA_8_ markedly increased the thermal stability of this G4, with no complete thermal denaturation observed over the experimental temperature range (Δ*T*_½_ > 25 °C relative to PNA_8_/*c-KIT1*_*32*_) (Fig. 6D). Thus, in both model constructs, conjugation of PDC to PNA_8_ resulted in markedly enhanced stabilization of the targeted G4.

Temperature-dependent ^1^H NMR experiments corroborated the CD results. Both 
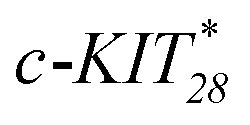
 and *c-KIT1*_*32*_ showed weak or nearly undetectable signals in the imino proton region, apart from Watson–Crick signals at 12.8 and 12.9 ppm, consistent with base-pairing interactions mediated by the flanking regions, which appeared more thermally persistent in *c-KIT1*_*32*_ (Fig. S18A and S19A). Upon addition of PNA_8_, new Watson–Crick imino resonances appeared around 12.6 ppm, consistent with DNA–PNA hybrid formation (Fig. S18B and S19B).

When the oligonucleotides were incubated with the corresponding PNA-ligand conjugates, Hoogsteen-type imino signals diagnostic of G4 formation became markedly more intense and thermally persistent (Fig. 7), in full agreement with CD data. To evaluate whether this behavior reflects synergistic effects arising from the combination of PNA hybridization with ligand binding, additional NMR experiments were carried out to compare the interactions of 
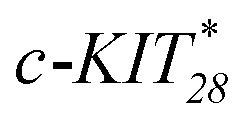
 and *c-KIT1*_*32*_ with the PNA-ligand conjugates to those with (i) PDC-β-Ala alone and (ii) a mixture of unconjugated PNA_8_ and PDC-β-Ala (Fig. S18C, D, S19C and D). Interestingly, for both systems, the effects observed with the ligand-conjugated PNAs are clearly not simply the sum of the individual contributions of unconjugated PNA_8_ and PDC-β-Ala. Indeed, a markedly enhanced induction of G4 formation and consequent stabilization were evident. Notably, in the presence of PDC–PNA_8_, the Hoogsteen imino proton signals of 
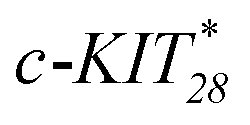
 exhibited a sharper and more distinct fingerprint characteristic of the c-KIT*/PDC complex, while the resulting G4 structure was significantly more stable than that formed in the presence of PDC-β-Ala alone, demonstrating enhanced stabilization through PNA conjugation (Fig. 7A). Similarly, PNA_8_–PDC produced sharper and better-resolved *c-KIT1*_*32*_ imino signals, particularly between 37 and 55 °C (Fig. 7B), confirming the exceptional stabilization of the G4 structure.

**Fig. 7:**
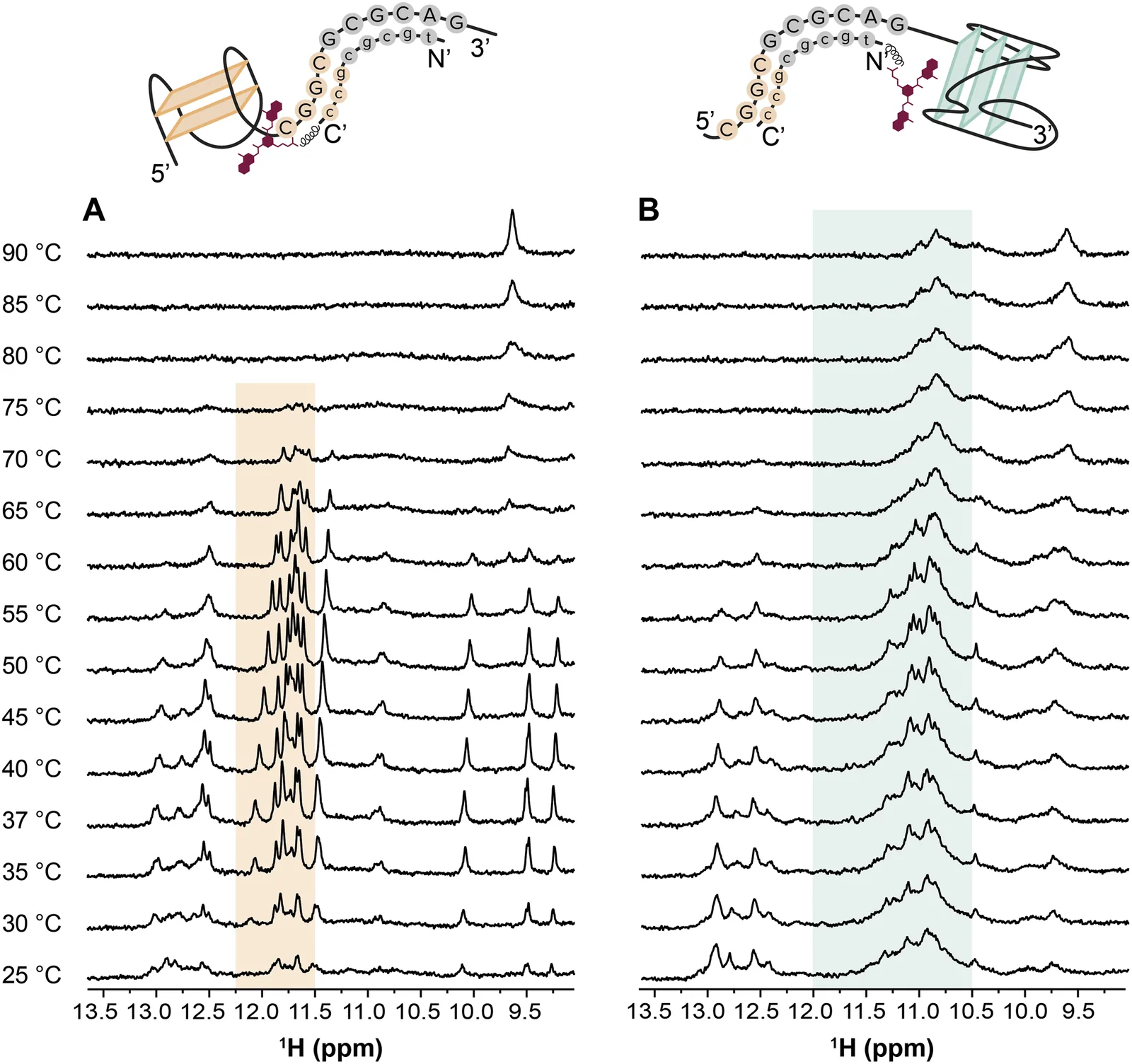
Imino region of 1D ^1^H NMR spectra acquired at various temperatures for (A) 
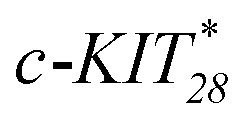
 and (B) *c-KIT1*_*32*_ (0.05 mM) in the presence of one equivalent of PDC–PNA_8_ and PNA_8_–PDC, respectively. All spectra were acquired in 5 mM phosphate buffer with 20 mM KCl, pH 7.0 (H_2_O/D_2_O, 90 : 10). Schematic structures above the spectra are for illustrative purposes only.

A mechanistic explanation for this synergistic behavior is that PNA hybridization anchors the ligand in close proximity to the G4, effectively increasing its local concentration. This tethering effect accelerates ligand rebinding events and enhances the overall probability of productive interactions, ultimately generating effects that exceed those expected from the individual components. Overall, this direct comparison strengthened the proof-of-concept that PNA probes can successfully guide ligands not only to a more selective, but also to a more effective G4 binding.

Given the limited discrimination of the PDC scaffold among different G4 targets, we also examined whether an unrelated G4 could interfere with the binding of a PDC-conjugated PNA_8_ probe to its *c-KIT* target and thereby attenuate target stabilization. For this purpose, the 
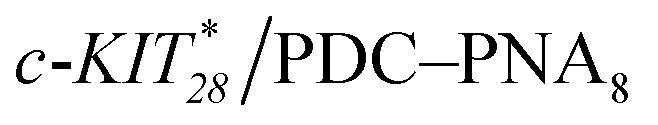
 system was selected, and the *c-MYC* promoter G4 was used as a competitor. The *c-MYC* G4 was chosen as it forms a highly stable parallel structure (*T*_½_ > 78 °C) under these buffer conditions and therefore remains predominantly folded over the temperature range encompassing the 
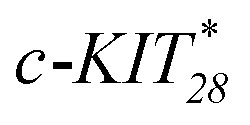
 transition.^44^ Moreover, it contributes minimally to the CD signal at 290 nm,^44^ allowing the thermal unfolding of 
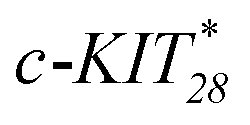
 to be selectively monitored at this wavelength. In an equimolar mixture of 
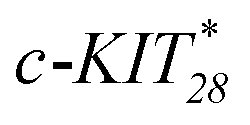
, *c-MYC* G4, and PDC–PNA_8_, the apparent *T*_½_ monitored at 290 nm was 66.7 ± 0.5 °C (Fig. S20), essentially identical to that measured for 
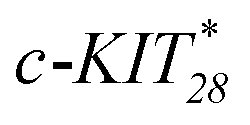
 in the presence of PDC–PNA_8_ without competitor (*T*_½_ = 67.0 ± 0.2 °C), and markedly higher than that of 
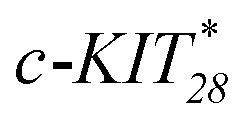
 alone (*T*_½_ = 52.8 ± 0.5 °C). Thus, the *c-MYC* G4 did not appreciably reduce PDC–PNA_8_-mediated stabilization of 
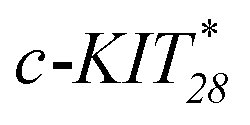
, supporting the robustness of PNA-guided target stabilization in the presence of an off-target G4.

To complement the CD melting results with an orthogonal assay, we performed a fluorescence-quenching competition experiment adapted from the proximal ligand-quenching approach.^40^ Cy5 was positioned adjacent to the targeted G4 domain in 
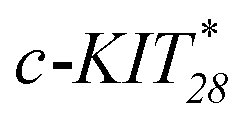
 and *c-KIT1*_*32*_, and the labelled oligonucleotides were combined with PDC–PNA_8_ and PNA_8_–PDC, respectively. In both systems, addition of the corresponding PNA-ligand conjugate markedly decreased Cy5 emission relative to the labelled DNA alone (Fig. S21). Efficient competition for the PDC moiety would be expected to reduce quenching and produce a progressive recovery of Cy5 fluorescence. However, increasing amounts of *c-MYC* G4, up to 10 equivalents, did not result in a progressive fluorescence recovery. For the *c-KIT1*_*32*_/PNA_8_–PDC system, addition of *c-MYC* G4 caused only minor changes in fluorescence intensity. For 
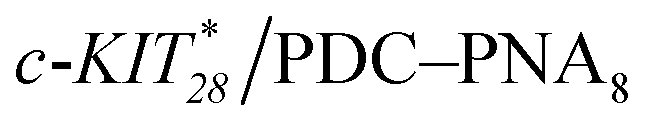
, a limited increase was observed at intermediate competitor ratios; however, the response was non-monotonic, and substantial quenching was retained at 10 equivalents of *c-MYC* G4. Thus, neither system showed the progressive, competitor-dependent fluorescence recovery expected if the competing G4 efficiently engaged the PDC moiety. Overall, these findings support the retention of PNA-guided target engagement in the presence of a competing G4.

### 2.7 PNA-mediated G4 recognition reveals distinct transcriptional outcomes

To assess the suitability of PNA probes in recognizing individual G4s in cells, we performed a cell-based dual luciferase assay (Fig. 8A). To this end, we employed a previously reported plasmid containing the *c-KIT* promoter sequence (including the *c-KIT2*, c-KIT*, and *c-KIT1* motifs) positioned upstream of the Renilla luciferase gene.^33^ Crucially, this plasmid also contains a Firefly luciferase gene that is under the control of a non G4-forming promoter, which can be used to reliably quantify changes in transcription modulated by a G4. The plasmid was incubated with PNA_8_ probes to ensure efficient strand invasion of the flanking region between c-KIT* and *c-KIT1,* before being transfected into HEK293T cells and incubated for 24 h. Untreated plasmid and plasmid treated with unconjugated PNA_8_ and PDC alone were included as controls.

**Fig. 8:**
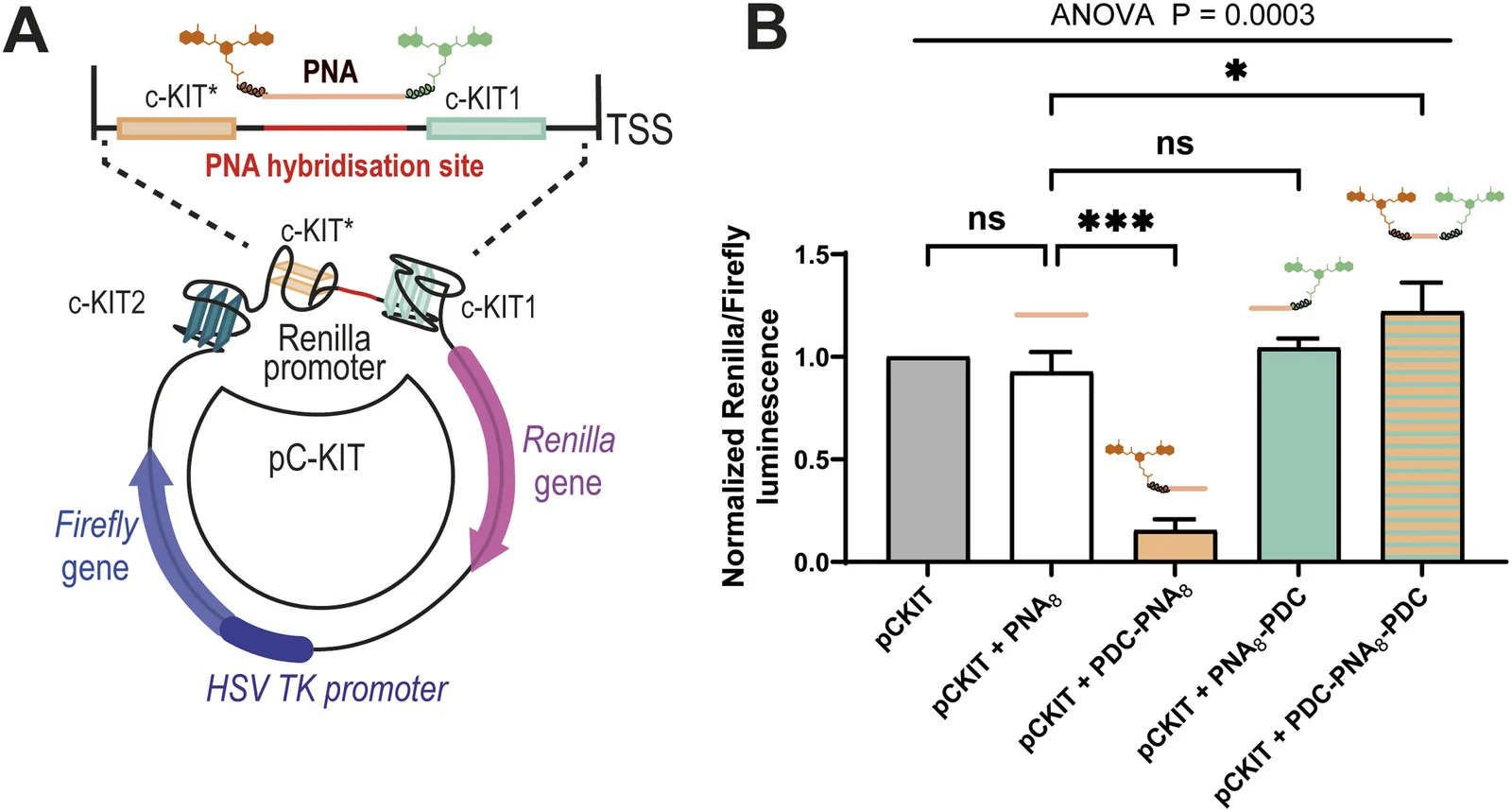
Selective targeting of *c-KIT* G4s leads to differential transcriptional outcomes. (A) Schematic representation of the plasmid construct used in the cell-based dual luciferase assay, with the *c-KIT* promoter region controlling Renilla expression. (B) Differential expression levels of Renilla luciferase in HEK293T cells upon selective targeting of *c-KIT1*, c-KIT*, or simultaneous targeting of both G4 motifs.

An initial screening was carried out to identify the optimal plasmid : PNA molar ratio required for effective strand invasion, and thus G4 stabilization. Using high excesses of PNA (200- and 1600-fold) to perform strand invasion proved detrimental to the plasmid's integrity, resulting in a luminescence readout comparable to that of non-transfected cells (data not shown). Considering the relatively short length of PNA_8_ (8-mer), this effect could be due to increased partial binding to unspecific sites on the plasmid, causing plasmid instability that inhibits proper gene expression. We therefore reduced the PNA excess to 10- and 50-fold. A 10-fold excess of PNA did not result in any statistically significant changes in gene expression, indicating an insufficient amount of PNA for optimal hybridization, whereas a 50-fold excess produced reproducible and interpretable effects. Consequently, all subsequent experiments were conducted using a 50-fold PNA excess.

Plasmid modified with unconjugated PNA_8_ exhibited Renilla luciferase expression levels comparable to the untreated plasmid (Fig. 8B), indicating that PNA invasion caused minimal perturbation to the construct, in line with the biophysical data.

Consistent with its promiscuous G4-binding properties, PDC alone induced a modest, statistically significant decrease (∼15%) in Renilla luciferase expression only at the highest tested concentration of 5 µM (the limit imposed by emerging cytotoxicity), while lower concentrations showed no significant effects (Fig. S22). This limited response can be attributed to the non-selective stabilization of the three G4 motifs present in the *c-KIT* promoter region.

Notably, ligand-decorated PNAs produced distinct transcriptional outcomes, indicating that the G4 motifs do not contribute equally to transcriptional regulation. In particular, selective stabilization of *c-KIT1* by PNA_8_–PDC did not significantly affect Renilla expression, consistent with the notion that this G4 adopts an intrinsically stable fold (see Fig. 2 and S3) and suggesting that it is not the limiting regulatory element within the *c-KIT* promoter.^45^ Conversely, targeting c-KIT* with PDC–PNA_8_ led to a marked decrease in reporter activity, indicating that enhanced ligand-mediated stabilization of this G4 is functionally repressive in this promoter. Importantly, these distinct transcriptional outcomes cannot be explained by global G4 stabilization, but instead reflect motif-specific regulatory effects arising from selective ligand positioning within the promoter.

Interestingly, simultaneous targeting of both *c-KIT1* and c-KIT* with PDC–PNA_8_–PDC resulted in a slight increase in Renilla expression. This may suggest that, upon dual stabilization of both G4s, the overall phenotype observed reflects what was observed when targeting the more stable species, in this case *c-KIT1*. The relatively modest increase in transcription observed under these conditions may reflect the increased accessibility provided by the presence of two folded G4s within the promoter. This provides functional evidence that selective targeting of adjacent G4s within a native promoter context can translate into differentiated transcriptional responses, supporting the biological relevance of the proposed targeting strategy. Although providing an unequivocal mechanistic explanation poses challenges that can only be overcome through extensive mutational studies,^46^ these results demonstrate that PNA conjugates can effectively target individual functional G4s within complex promoters and modulate their associated gene expression.

## 3 Conclusions

This study addressed the challenge of achieving site-specific targeting of individual G4s amongst adjacent G4 structures within a gene promoter using PNA-ligand conjugates. Through the development of PNA sequences complementary to the flanking region between two G4-forming motifs in the *c-KIT* promoter (c-KIT* and *c-KIT1*), we demonstrated that properly designed PNAs can hybridize to this region without significantly affecting G4 formation. Importantly, this hybridization strategy provides a means to specifically direct ligand binding, thereby offering a viable approach to mitigate the off-target effects that typically limit conventional small-molecule G4 ligands.

Conjugation of the most promising PNA probe, PNA_8_, with the well-known G4 ligand PDC, generated derivatives capable of recognizing the DNA target through base pairing while selectively stabilizing specific G4 structures through ligand binding. This dual-recognition design enabled precise discrimination between adjacent G4 motifs within the same promoter. Compared to alternative approaches such as polyamide-based recognition, which rely on specific structural features, PNA-based targeting enables direct base pairing with the DNA strand, thus providing precise spatial control over ligand positioning, a key factor in achieving selectivity within clustered G4 regions, and representing a more broadly applicable strategy.

Beyond biophysical validation, we provided proof-of-concept that such selective G4 stabilization in a cellular context translates into functional consequences at the transcriptional level. Notably, targeted stabilization of the individual *c-KIT1* and c-KIT* motifs resulted in distinct transcriptional outcomes, underscoring their non-equivalent regulatory roles within the *c-KIT* promoter. Although a comprehensive biological interpretation will require further investigation, these findings demonstrate that directing the ligand toward different G4 structures within a single promoter can differentially modulate the overall transcriptional outcome. The level of directional control and precision over adjacent G4 motifs afforded by PNA-mediated strand invasion makes these conjugates effective tools for both the precise targeting of individual G4s within regulatory elements containing multiple G4 structures and the systematic interrogation of their roles in gene regulation. It should be noted that cellular uptake of the PNAs was achieved through simultaneous transfection with the plasmid, as the main focus of this work was to interrogate the transcriptional consequences of specifically targeting G4s in this model system. Further functionalization of these PNAs with suitable cell-penetrating moieties, or their formulation in lipid-based or other delivery systems, would be required if these PNA-ligand conjugates were to be developed for therapeutic applications. In addition to delivery, extending this strategy to endogenous genomic loci will entail challenges related to chromatin accessibility and competition from nuclear proteins, which may influence target engagement and the resulting transcriptional outcome. Beyond the specific case of the *c-KIT* promoter, this work establishes a general framework for the selective targeting of individual or multiple G4 elements within complex genomic contexts. The ability to discriminate between closely spaced G4s represents an essential step toward the development of next-generation G4-targeting tools with improved precision and biological specificity.

Future studies will focus on evaluating the biological effects and binding specificity of PNA derivatives toward G4s in more complex cellular systems, to gain a deeper understanding of G4-dependent gene regulation and to further assess their therapeutic potential.

## Author contributions

R. G.: formal analysis, investigation, validation, writing – original draft; E. C.: formal analysis, investigation, validation, writing – original draft, writing – review & editing; S. M.: formal analysis, investigation, visualization, writing – original draft; V. A.: formal analysis, investigation, validation; N. F. A.: formal analysis, investigation, validation, visualization; M. D. A.: resources, supervision, writing – original draft, writing – review & editing; J. A.: resources, supervision, writing – original draft, writing – review & editing; A. M.: project administration, resources, supervision, writing – review & editing; B. P.: conceptualization, funding acquisition, project administration, resources, supervision, writing – original draft, writing – review & editing.

## Conflicts of interest

There are no conflicts to declare.

## Supplementary Material

SC-OLF-D6SC04697E-s001

## Data Availability

All data supporting the findings of this study are available within the article. Ref. 47–50 are cited in the supplementary information (SI). Supplementary information: experimental details, Tables S1–S3 and Fig. S1–S33. See DOI: https://doi.org/10.1039/d6sc04697e.
