# Supplementary material for: PNA-ligand conjugates enable site-selective and directional targeting of adjacent G-quadruplexes
Source: Chem Sci. 2026 Oct 5. Online ahead of print. doi: 10.1039/d6sc04697e (PMC13648328; doi:10.1039/d6sc04697e)
Supplement: Supplementary file 1 [file SC-OLF-D6SC04697E-s001.pdf]

Supplementary Information (SI) for Chemical Science.
This journal is © The Royal Society of Chemistry

Supplementary Information

PNA-ligand conjugates enable site-selective and directional targeting of adjacent G-quadruplexes

Raffaele Graziano,†[a] Enrico Cadoni,†[bc] Simona Marzano,[a] Valentina Arciuolo,[a] Naura Fakhira Antariksa,[c] Marco Di Antonio,[c] Jussara Amato,[a] Annemieke Madder *[b] and Bruno Pagano *[a]

[a]*Department of Pharmacy, University of Naples Federico II, Via D. Montesano 49, 80131 Naples, Italy. E-mail: bruno.pagano@unina.it*
[b]*Organic and Biomimetic Chemistry Research Group, Department of Organic and Macromolecular Chemistry, Ghent University, Krijgslaan 281-S4, 9000 Ghent, Belgium. E-mail: annemieke.madder@ugent.be*
[c]*Department of Chemistry, Molecular Sciences Research Hub, Imperial College London, 82 Wood Lane, London W12 0BZ, U.K.*

† These authors contributed equally.

*Corresponding authors.

TABLE OF CONTENTS PAGE

Table S1	S2
Fig. S1	S3
Fig. S2	S4
Fig. S3	S4
Table S2	S5
Fig. S4	S6
Fig. S5	S7
Fig. S6	S8
Fig. S7	S9
Fig. S8	S10
Fig. S9	S11
Fig. S10	S12
Fig. S11	S13
Fig. S12	S14
Fig. S13	S14
Fig. S14	S15
Fig. S15	S16
Fig. S16	S17
Fig. S17	S17
Fig. S18	S18
Fig. S19	S19
Fig. S20	S20
Fig. S21	S20
Fig. S22	S21
**Experimental Section**	S22
Materials	S22
Oligonucleotide synthesis and sample preparation	S22
Synthesis and characterization of PNA probes and PNA-PDC conjugates	S23
Fig. S23	S26
Fig. S24	S26
Fig. S25	S27
Fig. S26	S27
Fig. S27	S28
Fig. S28	S28
Fig. S29	S29
Fig. S30	S29
Fig. S31	S30
Fig. S32	S30
Synthesis and characterization of PDC-β-Ala	S31
Scheme S1	S32
Fig. S33	S33
Thermal difference spectra (TDS) analysis	S34
Polyacrylamide gel electrophoresis (PAGE) experiments	S34
Circular dichroism (CD) spectroscopy experiments	S34
Nuclear magnetic resonance (NMR) experiments	S35
Fluorescence titration experiments	S35
Fluorescence quenching competition experiments	S35
HEK293T cells maintenance	S36
PNA invasion of pC-KIT plasmid	S36
Plasmid transfection and Luciferase assay	S36
Table S3	S37
Luciferase assay for PDC treatment	S37
**References**	S37

Table S1. PNA sequences designed and used in this study.

Name	Sequence (C'→N')[a]
PNA_6	NH_2-[Ac]KR-**cgcgtc**-Ac
PNA_8	NH_2-[Ac]KR-**ccgcgcgt**-Ac
PNA_9	NH_2-[Ac]KR-**gccgcgcgt**-Ac
PNA_{10}	NH_2-[Ac]KR-**gccgcgcgtc**-Ac
PNA_8-PDC	NH_2-[Ac]KR-**ccgcgcgt**-O-PDC
PDC-PNA_8	NH_2-[PDC-O]KR-**ccgcgcgt**-Ac
PDC-PNA_8-PDC	NH_2-[PDC-O]KR-**ccgcgcgt**-O-PDC
CTRL-PDC	NH_2-[Ac]KR-**cgtcttcg**-O-PDC
PDC-CTRL	NH_2-[PDC-O]KR-**cgtcttcg**-Ac
PDC-CTRL-PDC	NH_2-[PDC-O]KR-**cgtcttcg**-O-PDC

[a] PNA sequences (lowercase letters in bold) are reported from C' to N' to facilitate base pair comparison with DNA sequences in Table 1. "PDC" refers to PDC-β-Ala derivative; "O" refers to AEEA spacer; "Ac" refers to acetyl group.

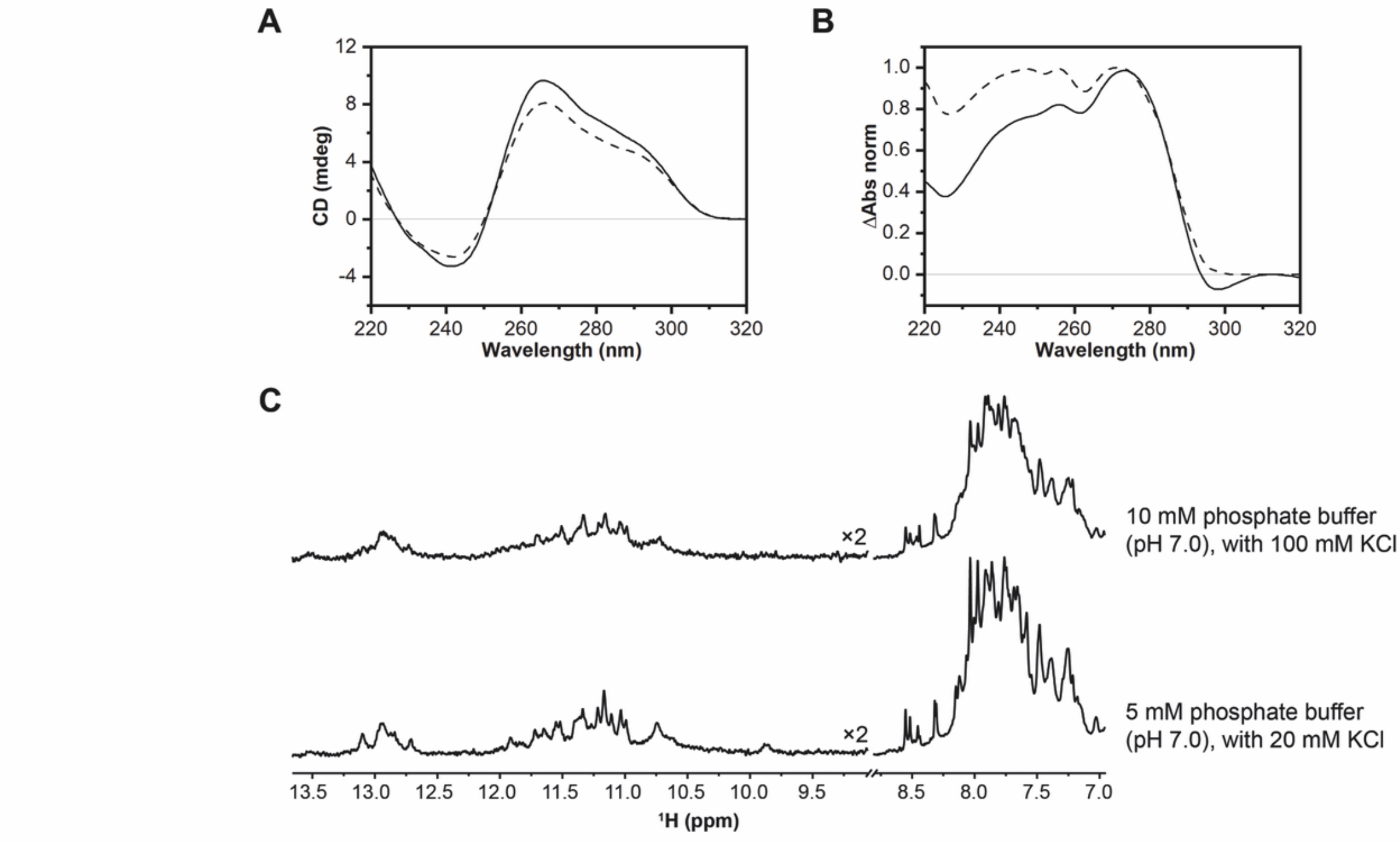

Fig. S1. (**A**) CD spectra at 20 °C and (**B**) normalized thermal difference absorbance spectra (TDS) of *c-KIT*$_{50}$ in 5 mM phosphate buffer with 20 mM KCl (solid line) or in 10 mM phosphate buffer with 100 mM KCl (dashed line) (pH 7.0). (**C**) Imino and aromatic regions of the ^{1}H NMR spectra of *c-KIT*$_{50}$ under high or low ionic strength conditions (H_2O/D_2O, 90:10) at 25 °C.

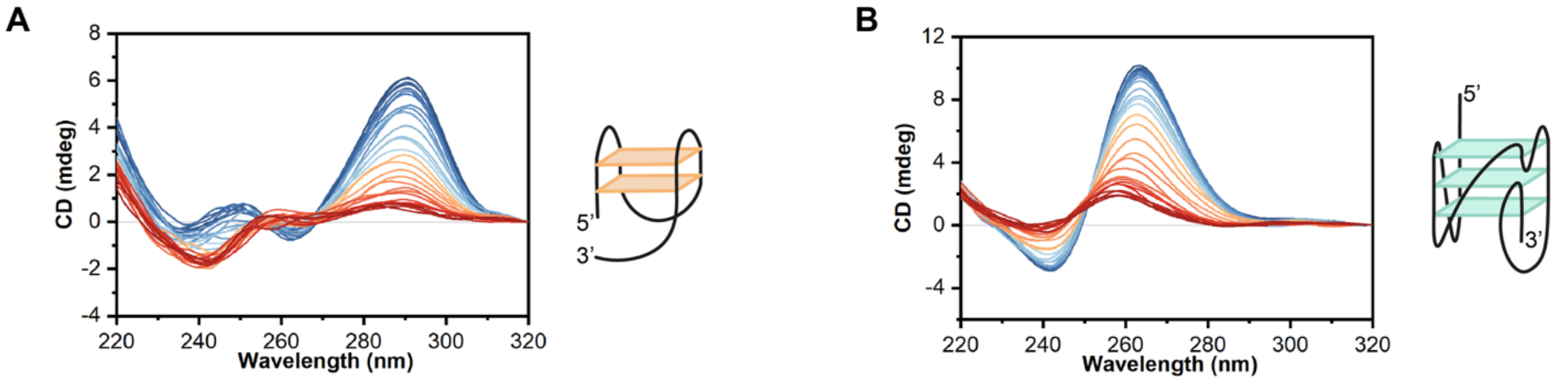

Fig. S2. Temperature-dependent CD spectra of (**A**) *c-KIT** and (**B**) *c-KIT1* from 20 °C to 80 °C (color-coded from blue to red) in 5 mM phosphate buffer with 20 mM KCl (pH 7.0).

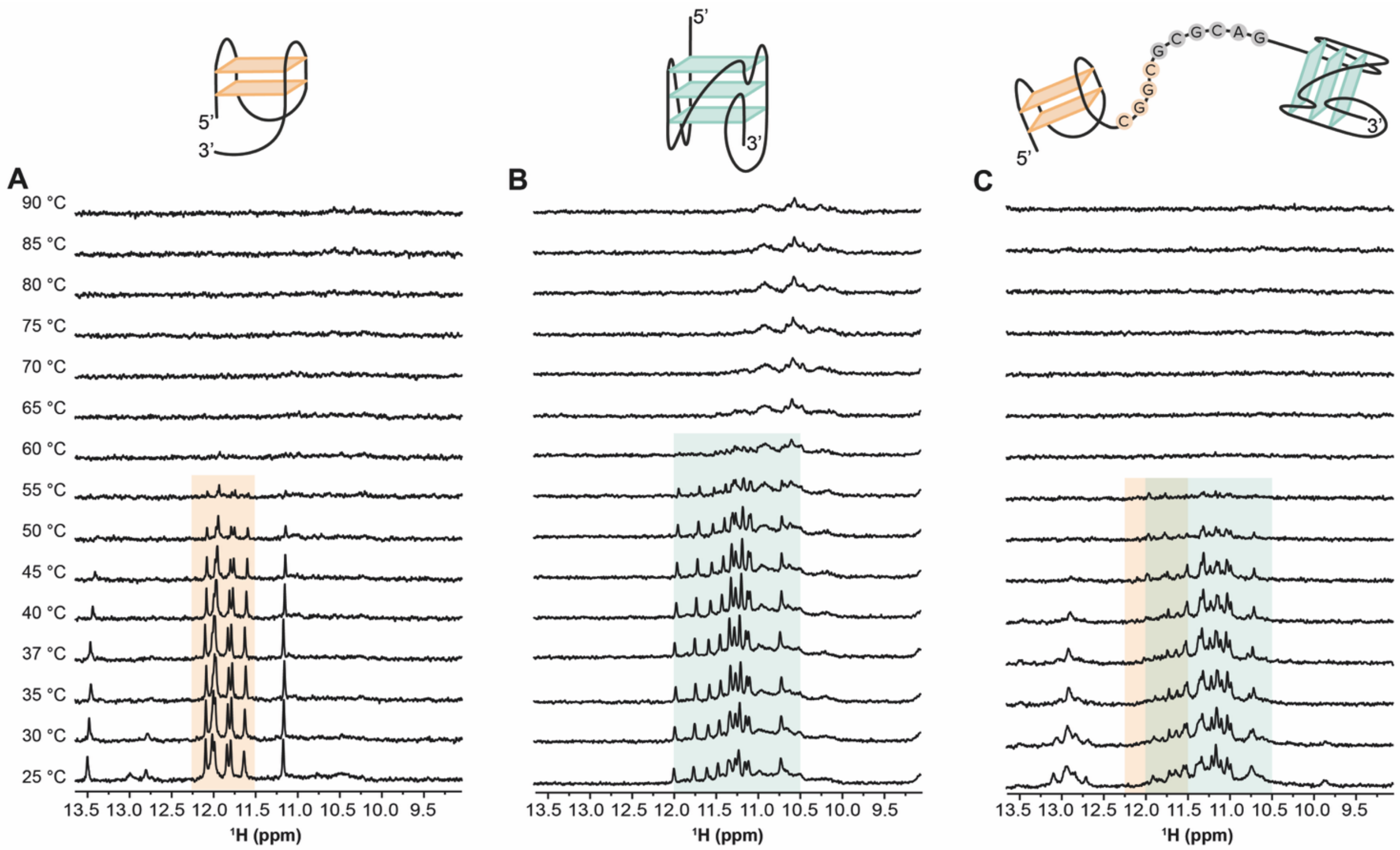

Fig. S3. Imino region of the 1D ^{1}H NMR spectra acquired at various temperatures for (**A**) *c-KIT**, (**B**) *c-KIT1*, and (**C**) *c-KIT$_{50}$* (0.05 mM) in 5 mM phosphate buffer with 20 mM KCl, pH 7.0 (H_2O/D_2O, 90:10). Schematic structures above the spectra are for illustrative purposes only.

Table S2. Thermal stability of *c-KIT**, *c-KIT1*, $c\text{-}KIT_{50}$, $c\text{-}KIT^*_{28}$, and $c\text{-}KIT1_{32}$ in the absence and presence of PDC-β-Ala or the indicated PNAs, monitored by CD melting experiments at 264 and 290 nm.

	$T_{½}$ (°C)	
	**264 nm**	**290 nm**
*c-KIT**	-	46.4 (±0.3)
c-KIT1	53.1 (±0.2)	-
*c-KIT** + PDC-β-Ala	-	62.3 (±0.4)
c-KIT1 + PDC-β-Ala	64.5 (±0.4)	-
$c\text{-}KIT_{50}$	42.1 (±0.1)	46.7 (±0.4)
$c\text{-}KIT_{50}$ + PDC-β-Ala[a]	56.5 (±0.2)	71.5 (±0.4)
$c\text{-}KIT_{50}$ + PNA_6	44.2 (±0.2)	46.1 (±0.5)
$c\text{-}KIT_{50}$ + PNA_8	45.9 (±0.2)	44.3 (±0.3)
$c\text{-}KIT_{50}$ + PNA_9	49.2 (±0.2)	n.a.[b]
$c\text{-}KIT_{50}$ + PNA_{10}	49.5 (±0.3)	n.a.[b]
$c\text{-}KIT_{50}$ + PDC-PNA_8-PDC	n.d.[c]	n.d.[c]
$c\text{-}KIT_{50}$ + PDC-PNA_8	51.3 (±0.5)	57.8 (±0.9)
$c\text{-}KIT_{50}$ + PNA_8-PDC	n.d.[c]	48.3 (±0.2)
$c\text{-}KIT_{50}$ + PDC-CTRL-PDC	n.d.[c]	55.8 (±0.7)
$c\text{-}KIT_{50}$ + PDC-CTRL	n.d.[c]	54.4 (±0.6)
$c\text{-}KIT_{50}$ + CTRL-PDC	n.d.[c]	50.7 (±0.8)
$c\text{-}KIT^*_{28}$	-	52.8 (±0.5)
$c\text{-}KIT^*_{28}$ + PNA_8	-	50.2 (±0.4)
$c\text{-}KIT^*_{28}$ + PDC-PNA_8	-	67.0 (±0.2)
$c\text{-}KIT1_{32}$	n.m.[d]	-
$c\text{-}KIT1_{32}$ + PNA_8	42.0 (±0.2)	-
$c\text{-}KIT1_{32}$ + PNA_8-PDC	n.d.[c]	-

[a] 1:2 molar ratio.
[b] n.a. = not applicable because the band was not detected.
[c] n.d. = not determined as no complete thermal denaturation was observed due to high stability.
[d] n.m. = not measurable because no thermal transition was detected.

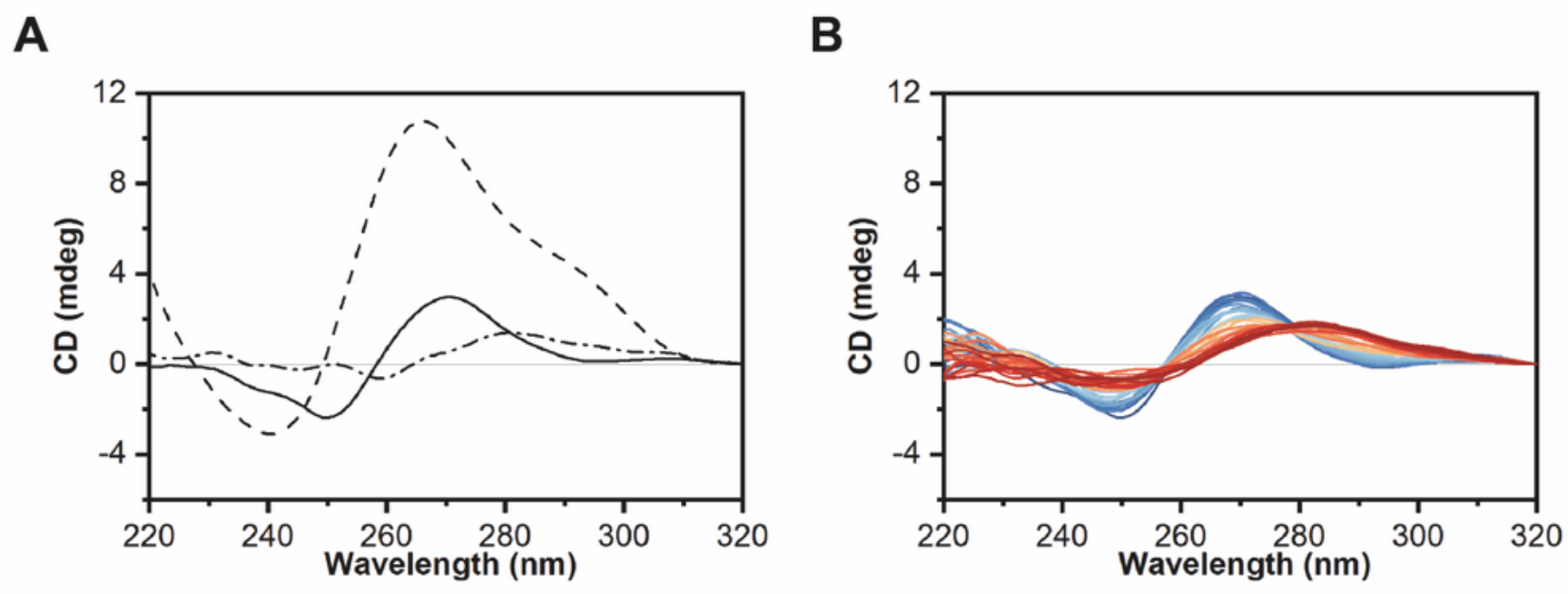

Fig. S4. (**A**) CD spectra of PNA_8 alone (dash-dotted line) and in the presence of the complementary *c-KIT*$_8$ (5'-GGCGCGCA-3', solid line) or *c-KIT*$_{50}$ (dashed line) (both at a 1:1 ratio with PNA_8) in 5 mM phosphate buffer with 20 mM KCl (pH 7.0). (**B**) Temperature-dependent CD spectra of PNA_8/*c-KIT*$_8$ mixture (1:1) from 20 °C to 90 °C (color-coded from blue to red).

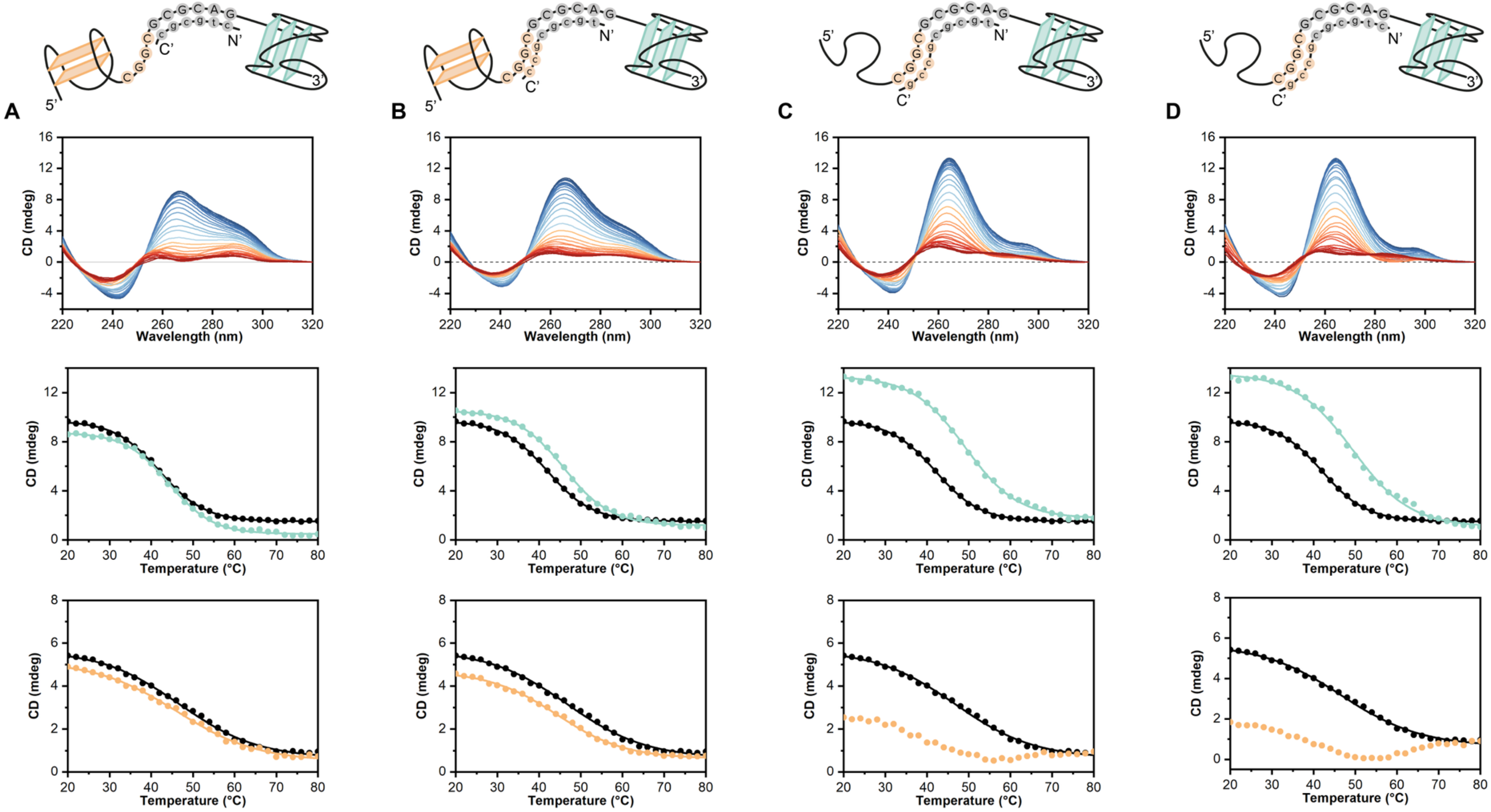

Fig. S5. CD analysis of *c-KIT*$_{50}$ in the presence of (**A**) PNA_6, (**B**) PNA_8, (**C**) PNA_9, and (**D**) PNA_{10}. Panels in the first row show CD spectra from 20 °C to 80 °C, color-coded from blue to red. Panels in the second and third rows show CD melting curves at 264 nm (green dots) and 290 nm (orange dots), respectively. For comparison, melting curves of *c-KIT*$_{50}$ without PNAs are shown as black dots. Fitted curves are shown as solid lines. Spectra were recorded in 5 mM phosphate buffer with 20 mM KCl (pH 7.0). Schematic structures above the spectra are for illustrative purposes only.

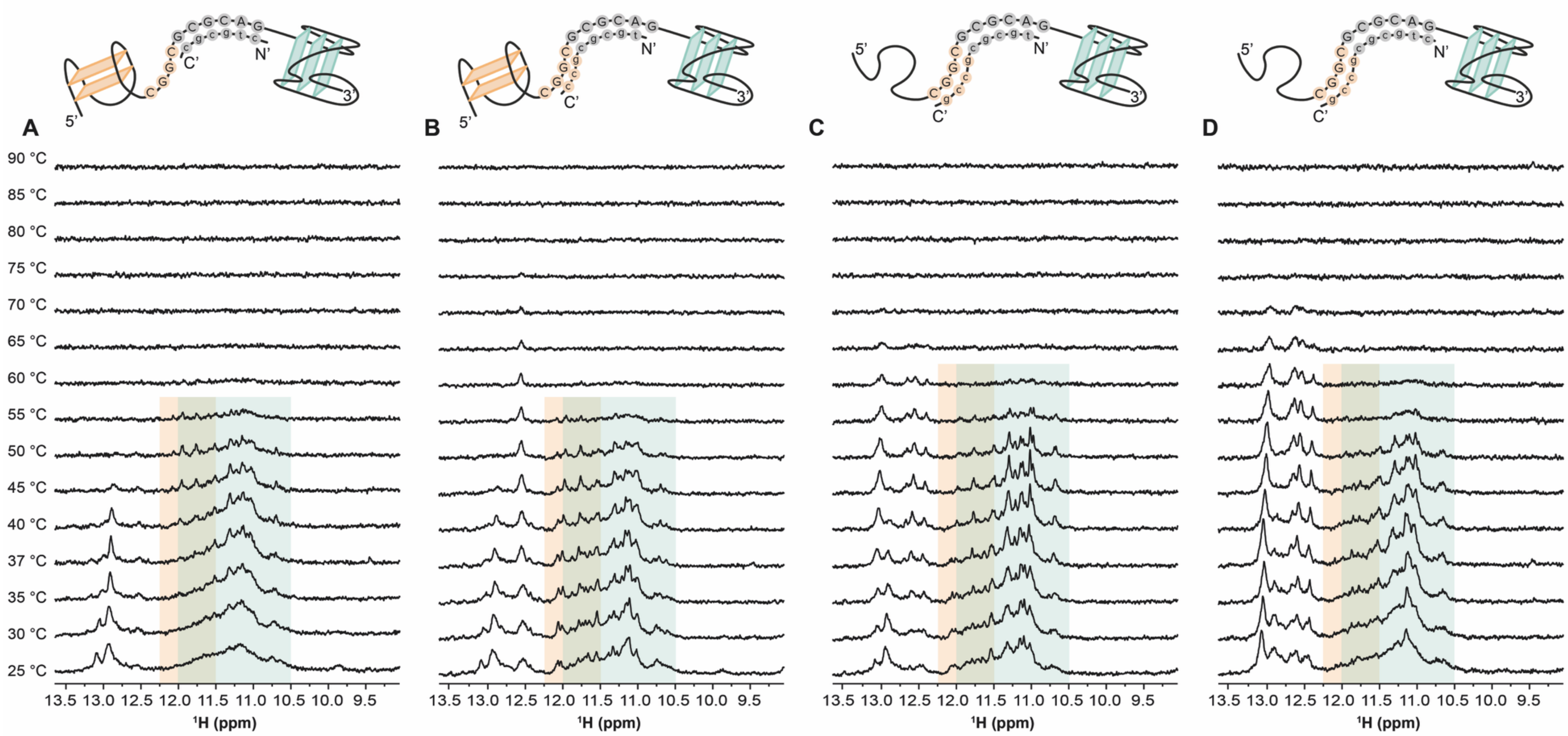

Fig. S6. Imino region of the 1D ^{1}H NMR spectra acquired at various temperatures for *c-KIT*$_{50}$ (0.05 mM) in the presence of (**A**) PNA$_6$, (**B**) PNA$_8$, (**C**) PNA$_9$, and (**D**) PNA$_{10}$ in 5 mM phosphate buffer with 20 mM KCl, pH 7.0 (H_2O/D_2O, 90:10). Schematic structures above the spectra are for illustrative purposes only.

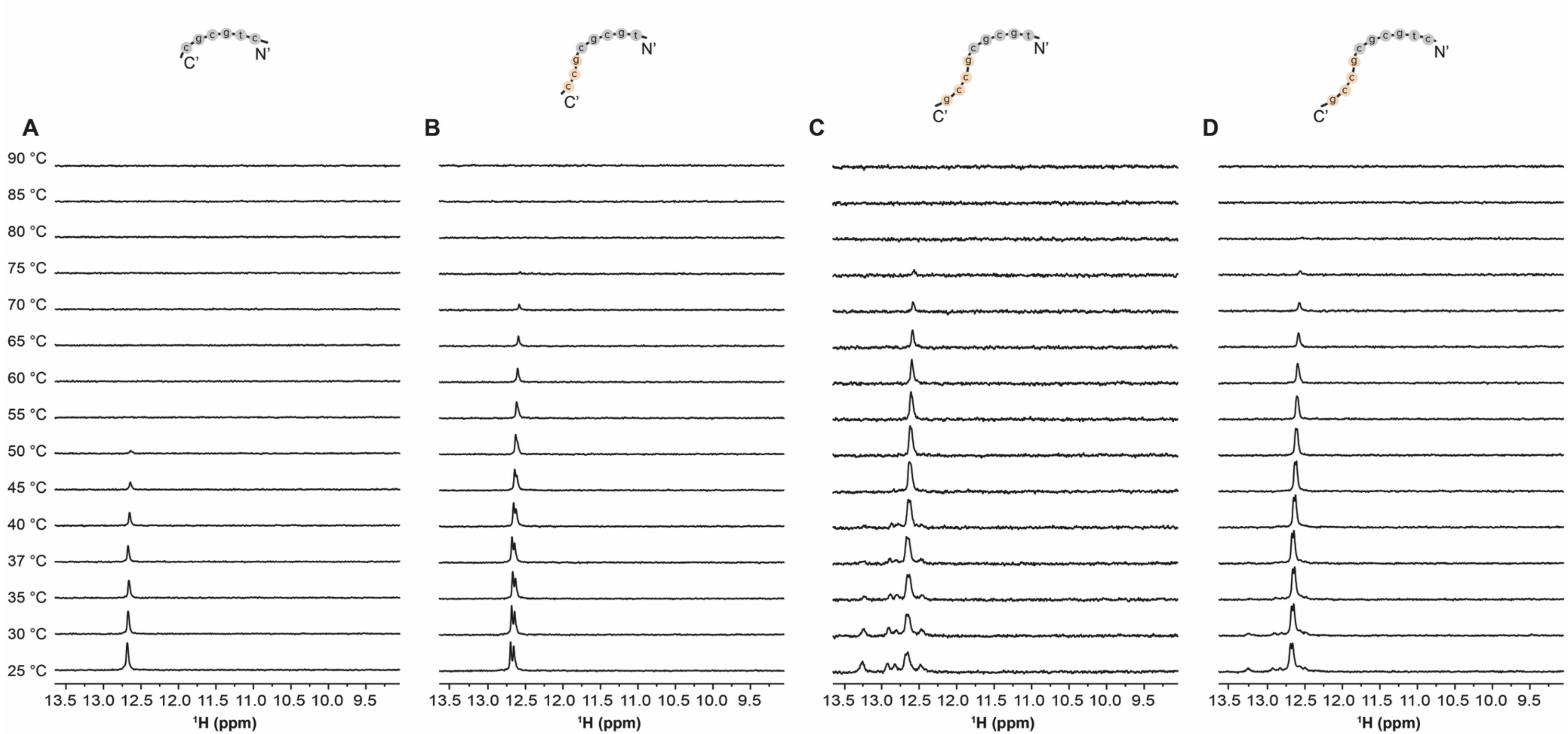

Fig. S7. Imino region of the 1D ^{1}H NMR spectra acquired at various temperatures for (**A**) PNA_6, (**B**) PNA_8, (**C**) PNA_9, and (**D**) PNA_{10} (0.05 mM) in 5 mM phosphate buffer with 20 mM KCl, pH 7.0 (H_2O/D_2O, 90:10). Schematic structures above the spectra are for illustrative purposes only.

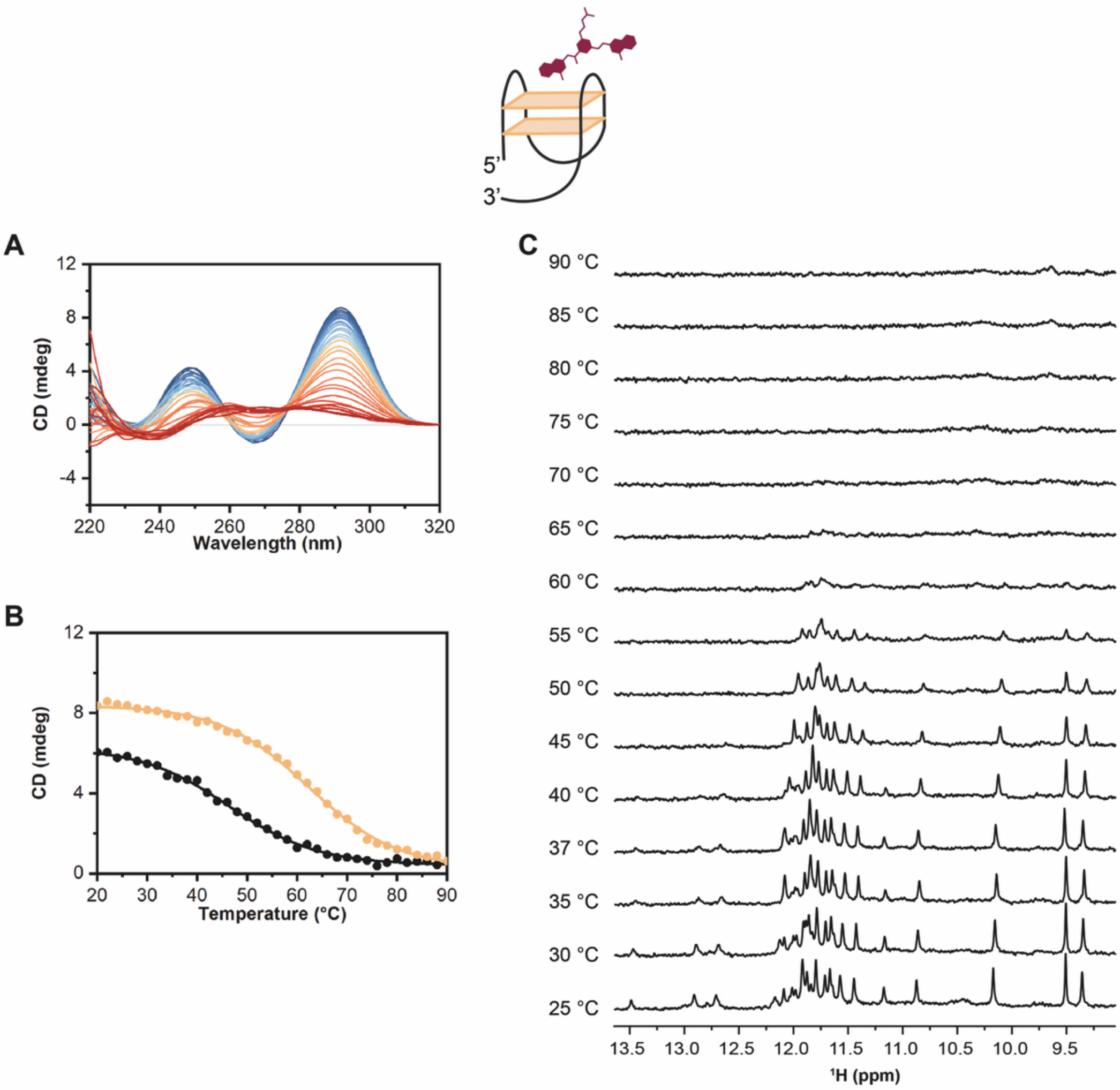

Fig. S8. CD and 1D ^{1}H NMR experiments for *c-KIT** in the presence of one equivalent of PDC-β-Ala. (**A**) CD spectra from 20 °C to 90 °C (color-coded from blue to red) and (**B**) CD melting curve at 290 nm (orange dots). For comparison, the melting curve of *c-KIT** in the absence of ligand is shown as black dots. Fitted curves are shown as solid lines. (**C**) Imino region of the ^{1}H NMR spectra of *c-KIT** (0.05 mM) acquired at various temperatures. All spectra were recorded in 5 mM phosphate buffer with 20 mM KCl (pH 7.0). NMR samples required H_2O/D_2O (90:10). Schematic structures above the spectra are for illustrative purposes only.

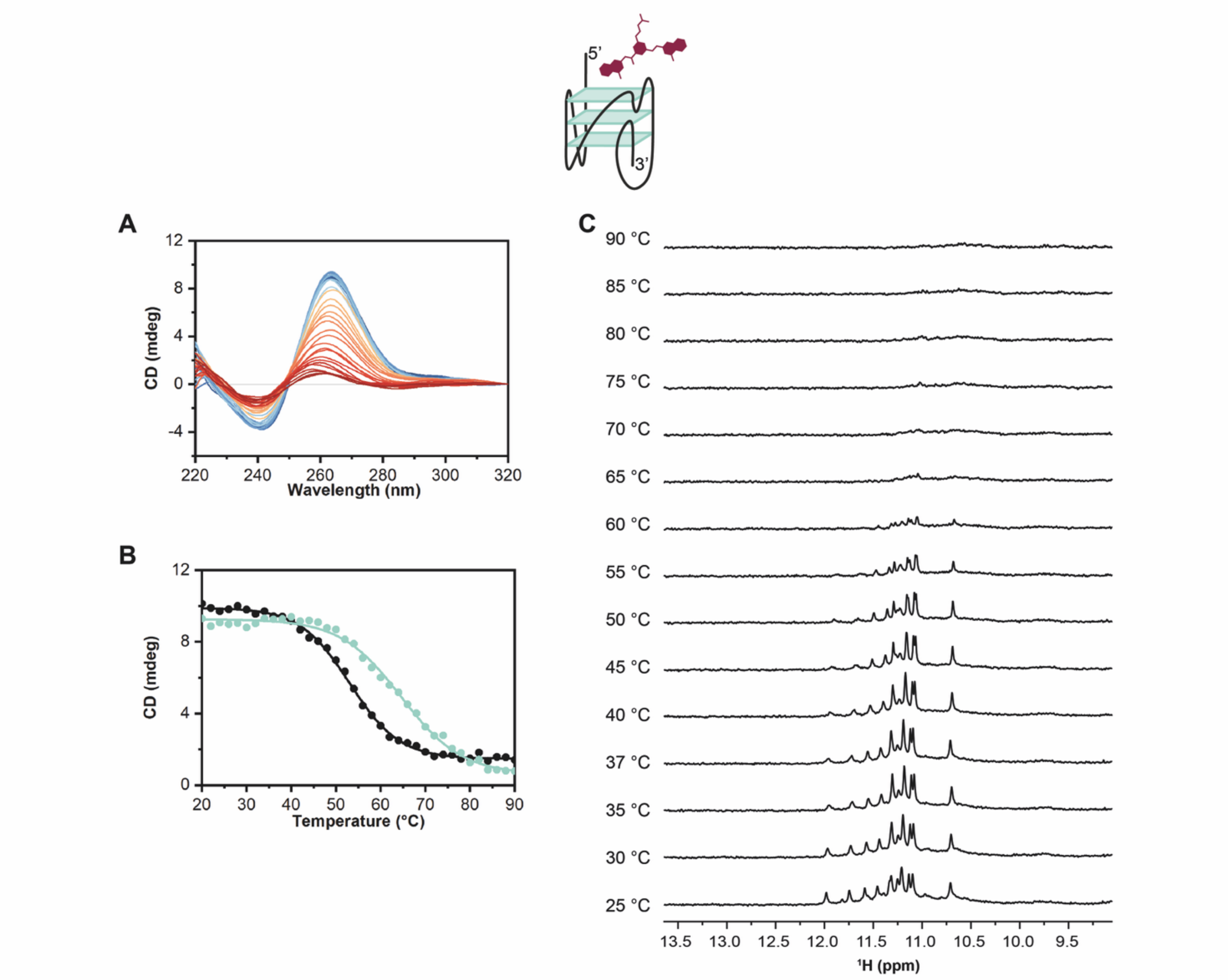

Fig. S9. CD and 1D ^{1}H NMR experiments for *c-KIT1* in the presence of one equivalent of PDC-β-Ala. (**A**) CD spectra from 20 °C to 90 °C (color-coded from blue to red) and (**B**) CD melting curve at 264 nm (green dots). For comparison, the melting curve of *c-KIT1* in the absence of ligand is shown as black dots. Fitted curves are shown as solid lines. (**C**) Imino region of the ^{1}H NMR spectra of *c-KIT1* (0.05 mM) acquired at various temperatures. All spectra were recorded in 5 mM phosphate buffer with 20 mM KCl (pH 7.0). NMR samples required H_2O/D_2O (90:10). Schematic structures above the spectra are for illustrative purposes only.

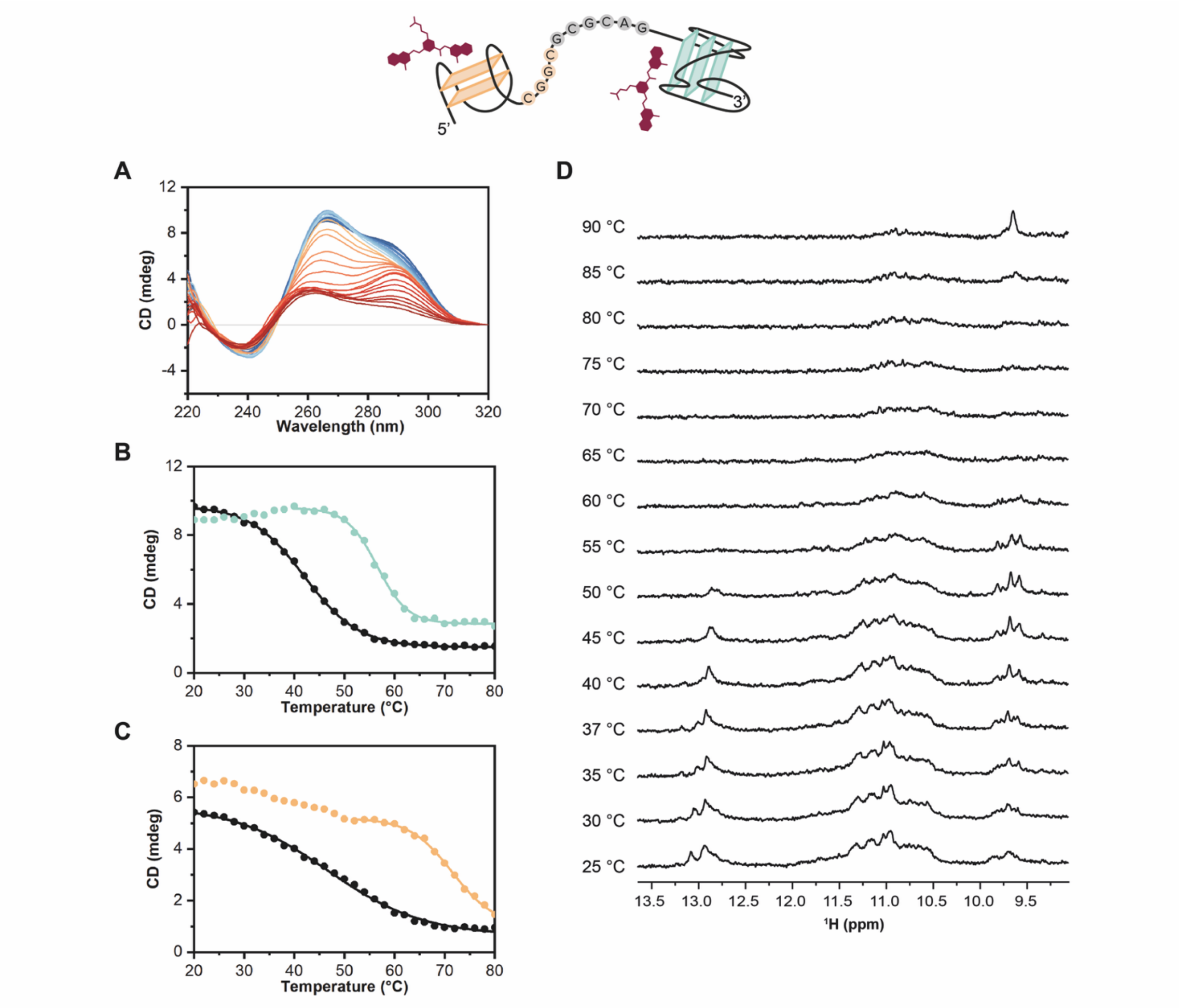

Fig. S10. CD and 1D ^{1}H NMR experiments for *c-KIT*$_{50}$ in the presence of two equivalents of PDC-β-Ala. (**A**) CD spectra from 20 °C to 80 °C (color-coded from blue to red) and (**B**,**C**) CD melting curves at 264 nm (green dots) and 290 nm (orange dots). For comparison, the corresponding melting curves of *c-KIT*$_{50}$ in the absence of ligand are shown as black dots. Fitted curves are shown as solid lines. (**D**) Imino region of the ^{1}H NMR spectra of *c-KIT*$_{50}$ (0.05 mM) acquired at various temperatures. All spectra were recorded in 5 mM phosphate buffer with 20 mM KCl (pH 7.0). NMR samples required H_2O/D_2O (90:10). Schematic structures above the spectra are for illustrative purposes only.

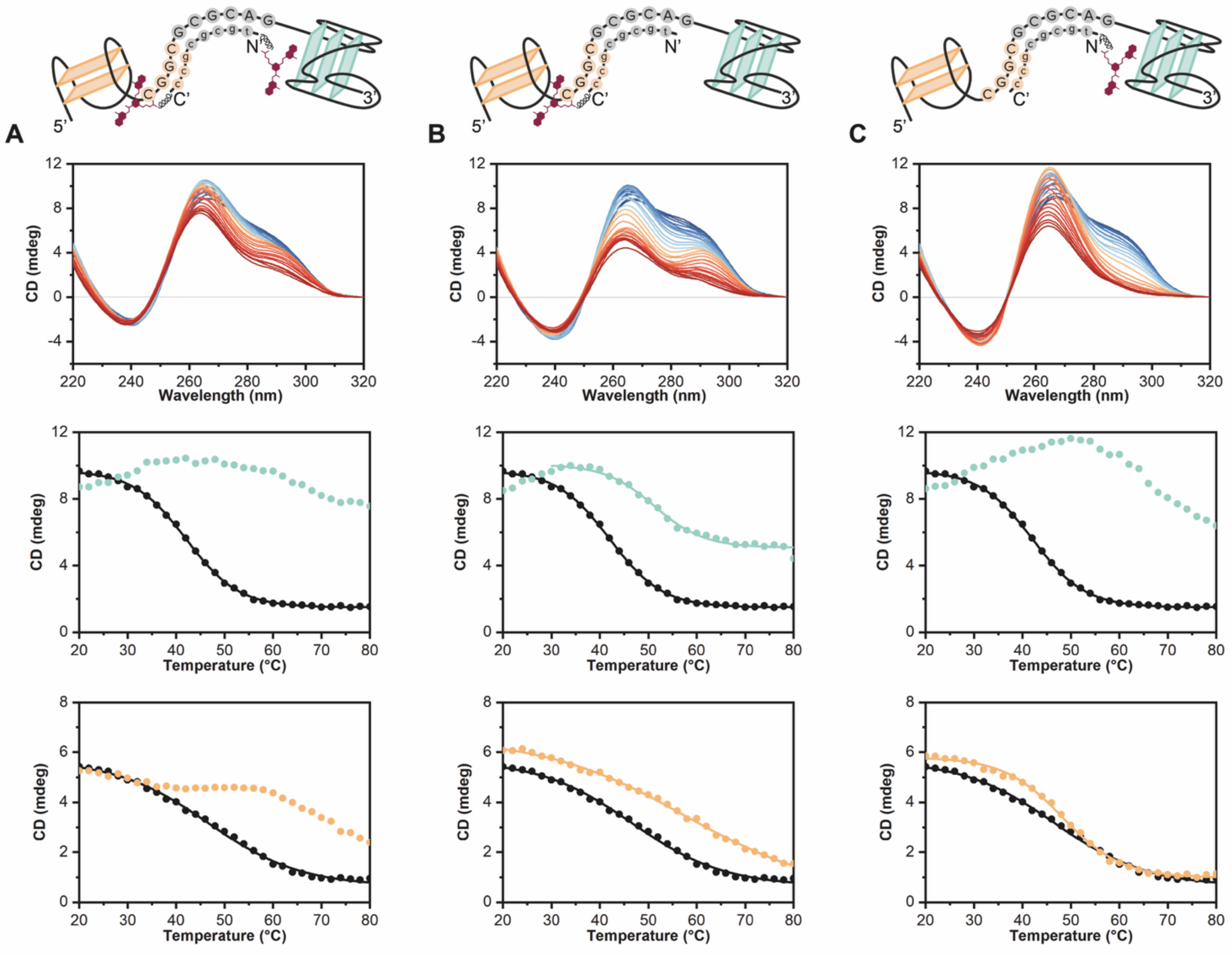

Fig. S11. CD analysis of *c-KIT*$_{50}$ in the presence of one equivalent of (**A**) PDC-PNA$_8$-PDC, (**B**) PDC-PNA$_8$, and (**C**) PNA$_8$-PDC. Panels in the first row show CD spectra from 20 °C to 80 °C, color-coded from blue to red. Panels in the second and third rows show CD melting profiles at 264 nm (green dots) and 290 nm (orange dots), respectively. For comparison, melting curves of *c-KIT*$_{50}$ without PNAs are shown as black dots. Fitted curves are shown as solid lines. Spectra were recorded in 5 mM phosphate buffer with 20 mM KCl (pH 7.0). Schematic structures above the spectra are for illustrative purposes only.

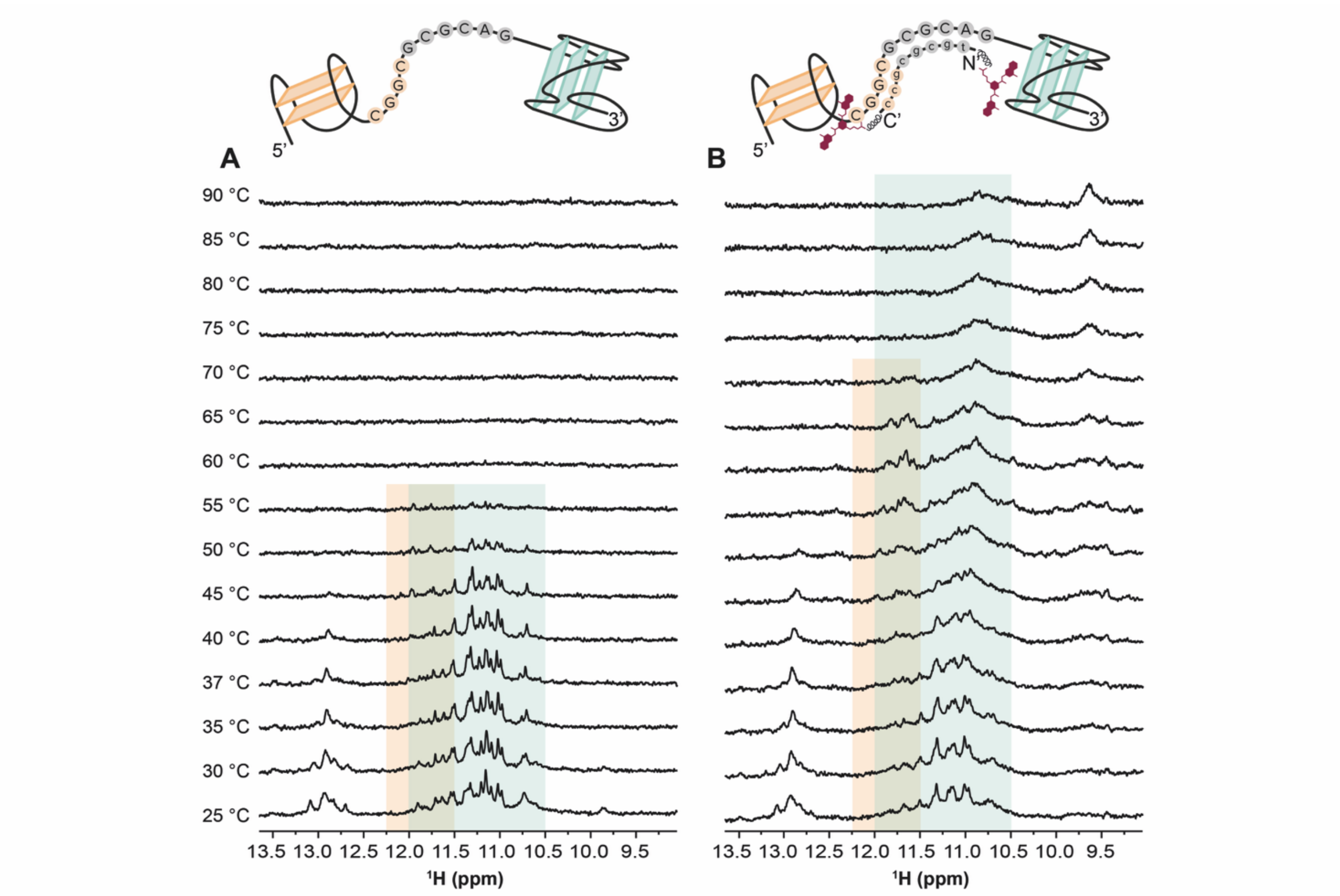

Fig. S12. Imino region of the 1D ^{1}H NMR spectra acquired at various temperatures for *c-KIT*$_{50}$ (0.05 mM) in the (**A**) absence and (**B**) presence of one equivalent of PDC-PNA$_8$-PDC in 5 mM phosphate buffer with 20 mM KCl, pH 7.0 (H_2O/D_2O, 90:10). Schematic structures above the spectra are for illustrative purposes only.

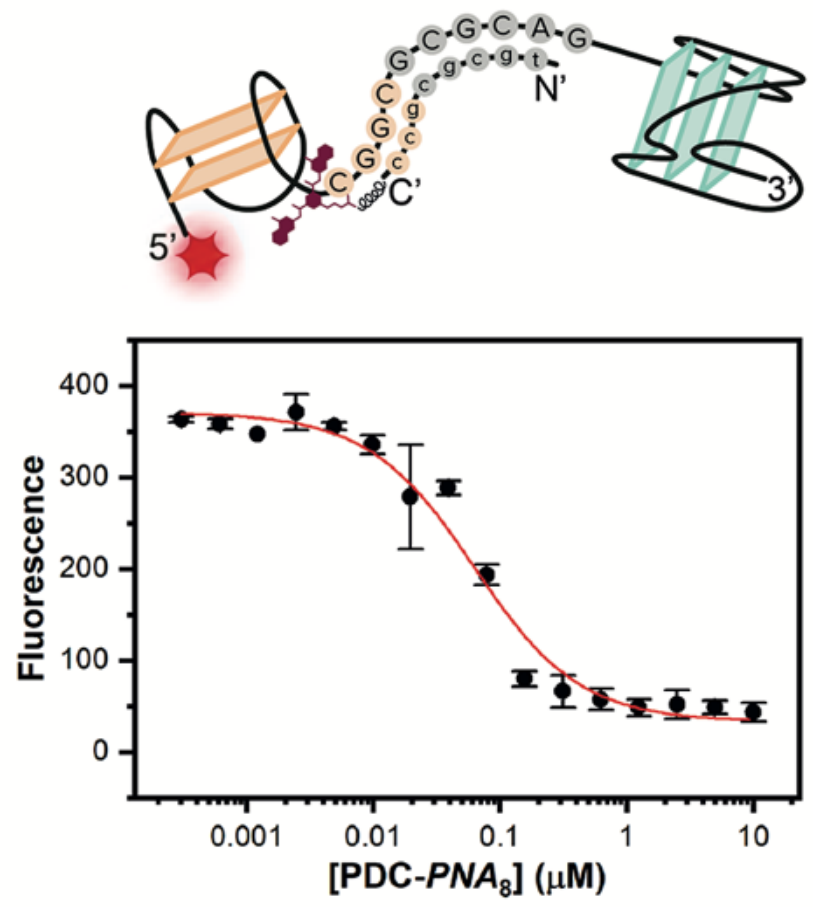

Fig. S13. Fluorescence quenching of 5′-Cy5.5-labelled *c-KIT*$_{50}$ (10 nM) at different concentrations of PDC-PNA$_8$. The solid red line represents the nonlinear fit used to determine the K_d. Experiments were performed at 25 °C in 5 mM potassium phosphate buffer (pH 7.0) containing 20 mM KCl and 0.05% Tween-20. Schematic structures above the plot are for illustrative purposes only.

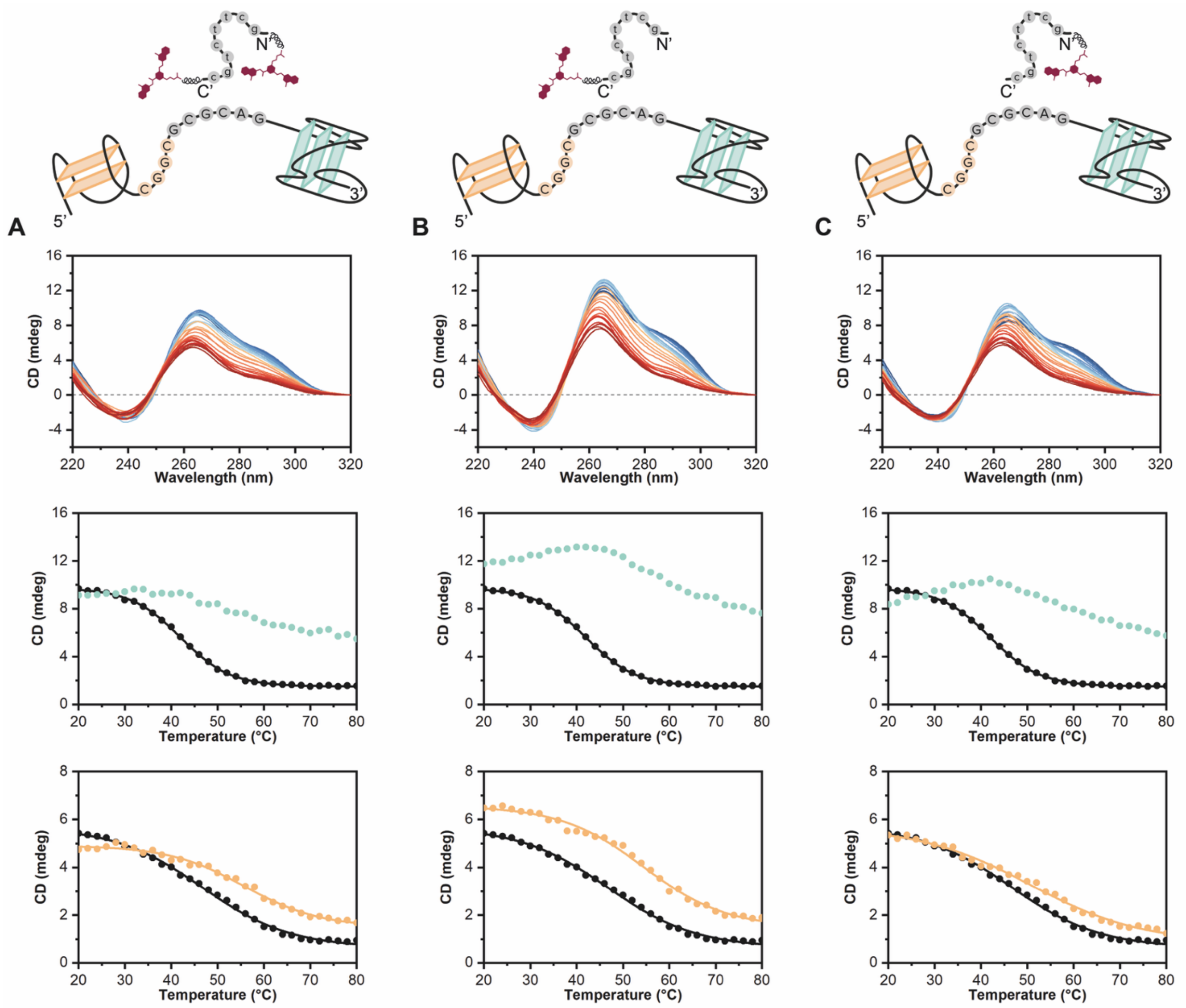

Fig. S14. CD analysis of *c-KIT*$_{50}$ in the presence of one equivalent of (**A**) PDC-CTRL-PDC, (**B**) PDC-CTRL, and (**C**) CTRL-PDC. Panels in the first row show CD spectra from 20 °C to 80 °C, color-coded from blue to red. Panels in the second and third rows show CD melting profiles at 264 nm (green dots) and 290 nm (orange dots), respectively. For comparison, melting curves of *c-KIT*$_{50}$ without PNAs are shown as black dots. Fitted curves are shown as solid lines. Spectra were recorded in 5 mM phosphate buffer with 20 mM KCl (pH 7.0). Schematic structures above the spectra are for illustrative purposes only.

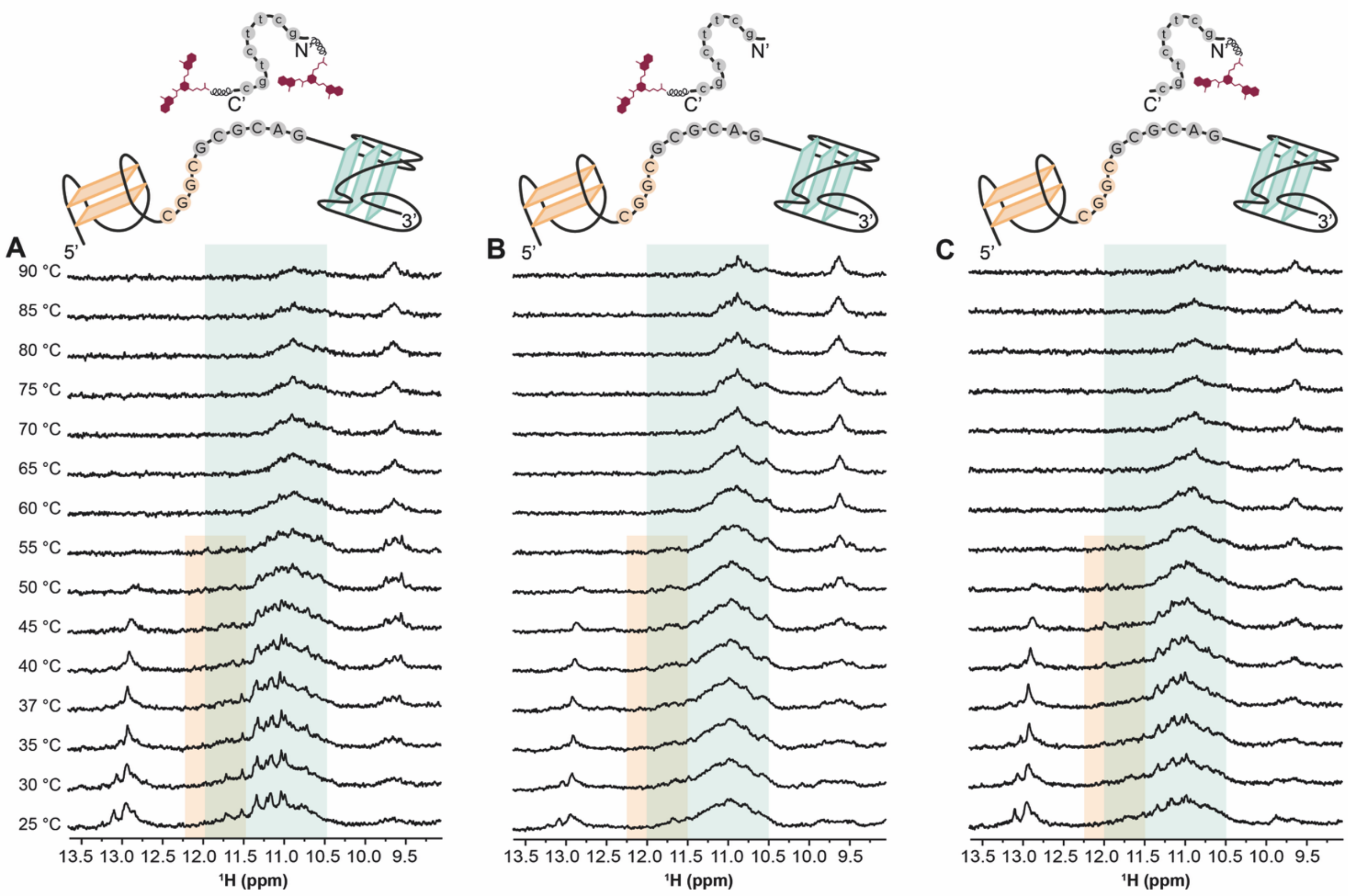

Fig. S15. Imino region of the 1D ^{1}H NMR spectra acquired at various temperatures for *c-KIT$_{50}$* (0.05 mM) in the presence of one equivalent of (**A**) PDC-CTRL-PDC, (**B**) PDC-CTRL, and (**C**) CTRL-PDC, in 5 mM phosphate buffer with 20 mM KCl, pH 7.0 (H_2O/D_2O, 90:10). Schematic structures above the spectra are for illustrative purposes only.

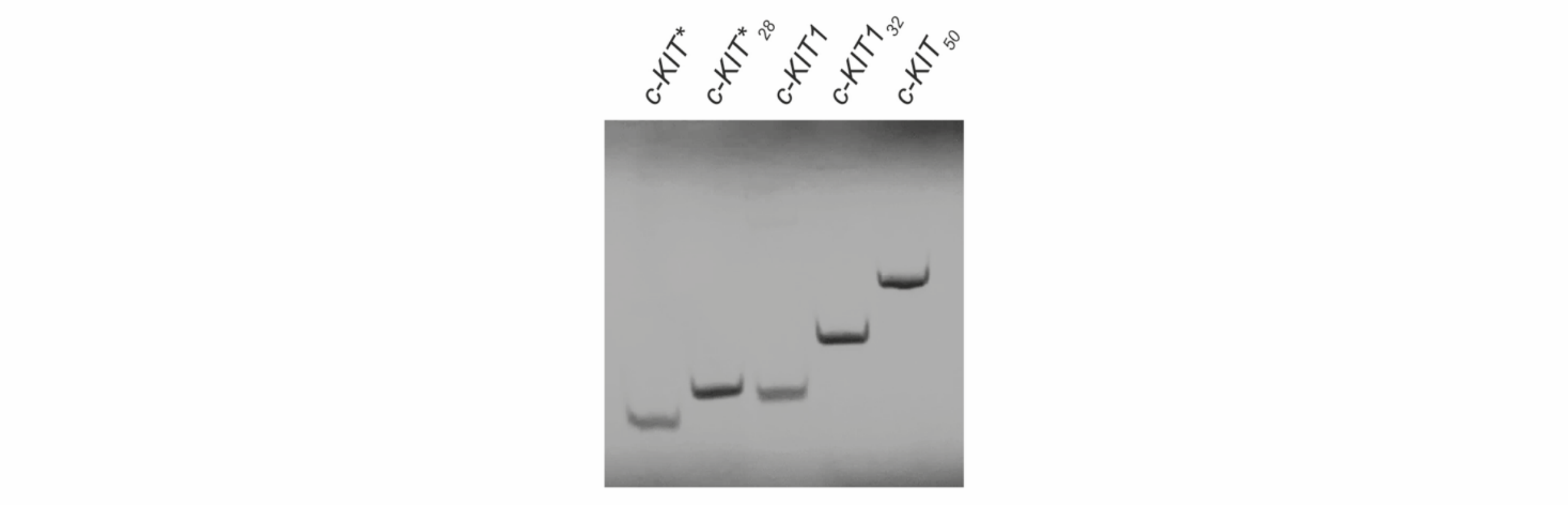

Fig. S16. Non-denaturing polyacrylamide gel electrophoresis of *c-KIT**, *c-KIT*$_{28}$*, *c-KIT1*, *c-KIT1$_{32}$*, and *c-KIT$_{50}$* sequences (0.05 mM) in 5 mM phosphate buffer with 20 mM KCl, pH 7.0.

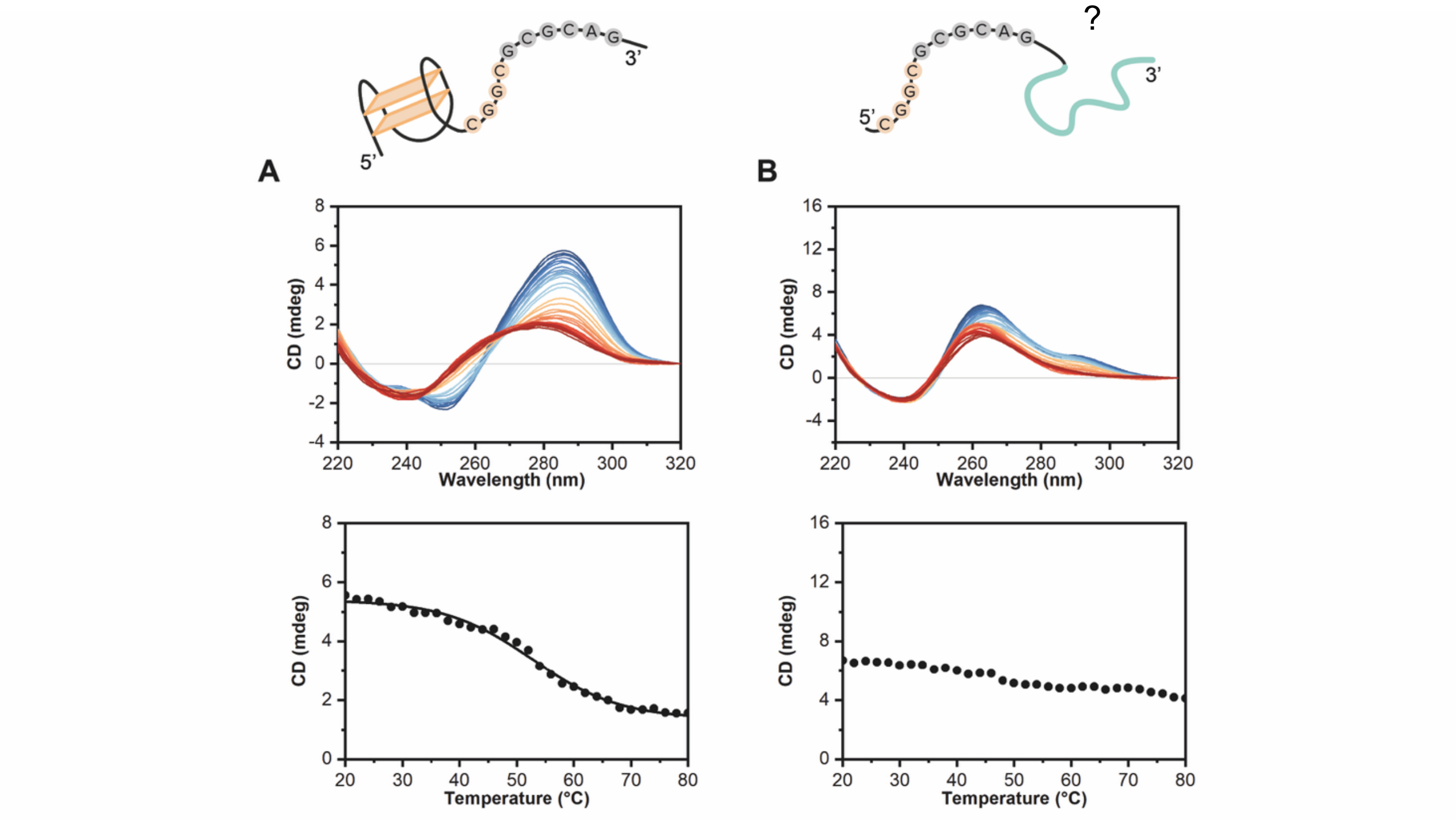

Fig. S17. CD experiments for (**A**) *c-KIT*$_{28}$* and (**B**) *c-KIT1$_{32}$*. The top panels show CD spectra from 20 to 90 °C, color-coded from blue to red. The bottom panels show CD melting curves at (**A**) 290 nm and (**B**) 264 nm. Fitted curve is shown as a solid line. Spectra were recorded in 5 mM phosphate buffer with 20 mM KCl (pH 7.0). Schematic structures above the spectra are for illustrative purposes only. The "?" symbol indicates spectral features that do not support the presence of a defined G4 structure.

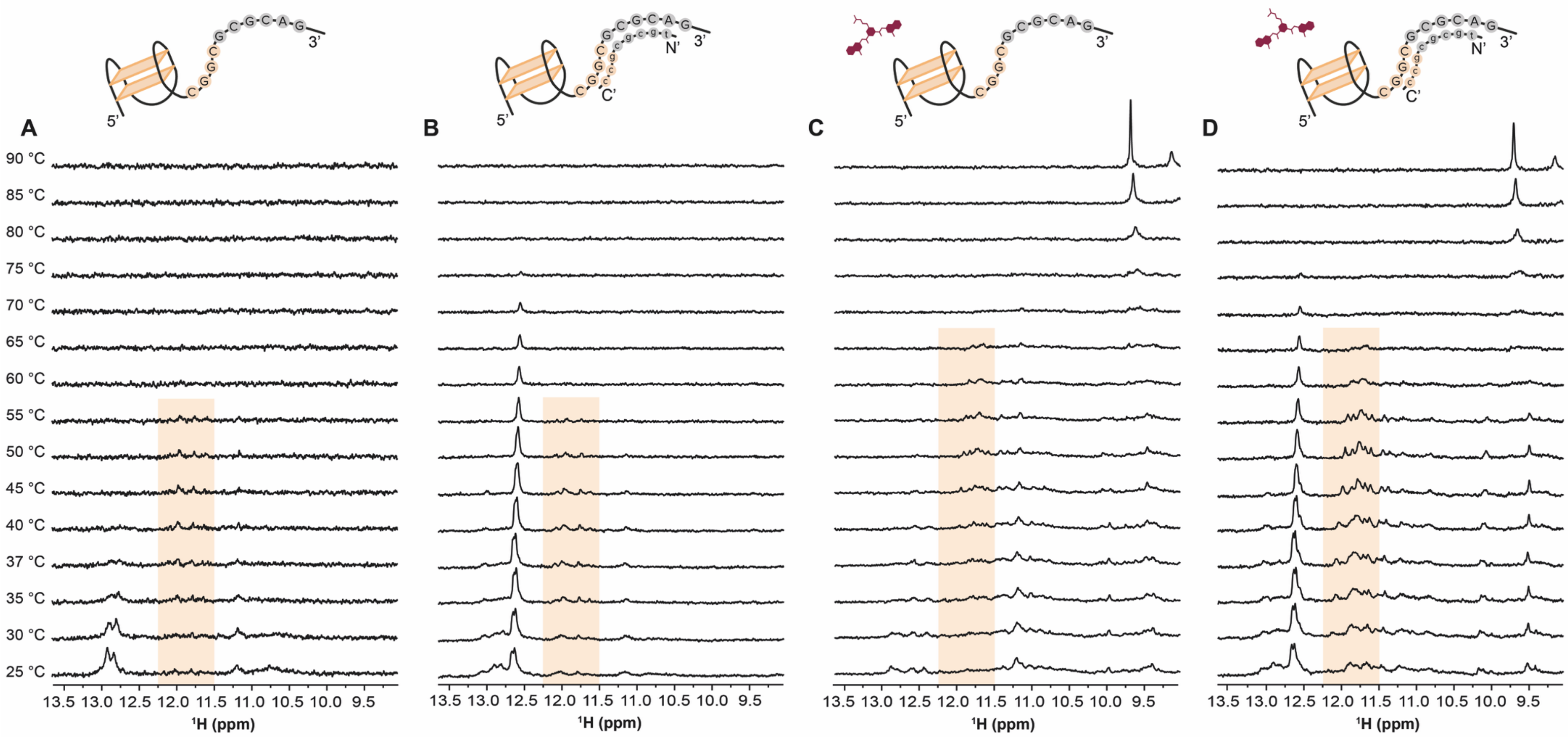

Fig. S18. Imino region of the 1D ^{1}H NMR spectra acquired at various temperatures for *c-KIT*$^{*}_{28}$ (0.05 mM) in the (**A**) absence and presence of one equivalent of (**B**) PNA_8, (**C**) PDC-β-Ala, and (**D**) PNA_8 and PDC-β-Ala (1:1:1) in 5 mM phosphate buffer with 20 mM KCl, pH 7.0 (H_2O/D_2O, 90:10). Schematic structures above the spectra are for illustrative purposes only.

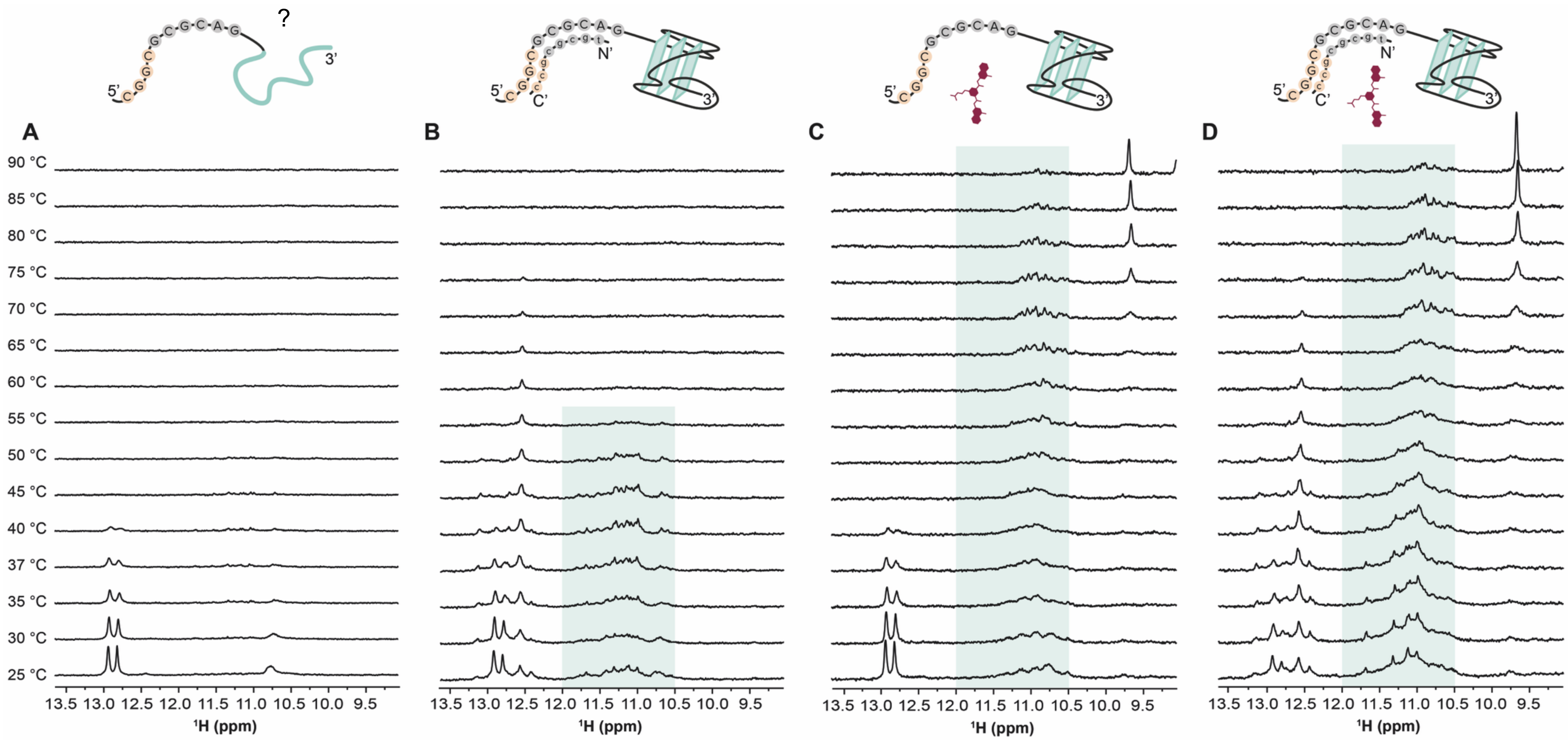

Fig. S19. Imino region of the 1D ^{1}H NMR spectra acquired at various temperatures for *c-KIT1*$_{32}$ (0.05 mM) in the (**A**) absence and presence of one equivalent of (**B**) PNA$_8$, (**C**) PDC-β-Ala, and (**D**) PNA$_8$ and PDC-β-Ala (1:1:1) in 5 mM phosphate buffer with 20 mM KCl, pH 7.0 (H_2O/D_2O, 90:10). Schematic structures above the spectra are for illustrative purposes only. The "?" symbol indicates spectral features that do not support the presence of a defined G4 structure.

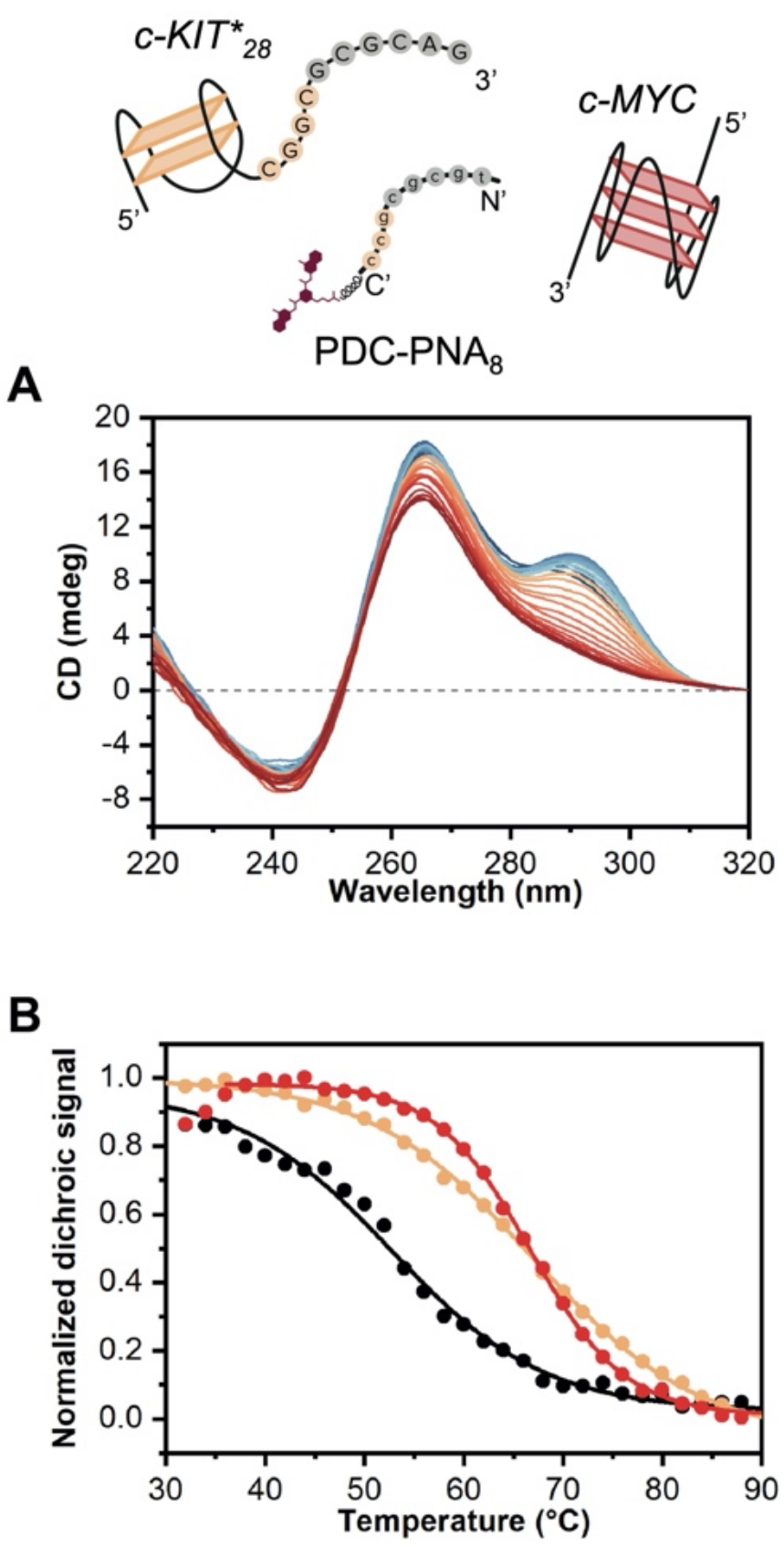

Fig. S20. CD competition experiment for *c-KIT*$^{*}_{28}$ in the presence of *c-MYC* G4 and PDC-PNA_8. (**A**) Temperature-dependent CD spectra of an equimolar mixture of *c-KIT*$^{*}_{28}$, *c-MYC*, PDC-PNA_8, color-coded from blue to red. (**B**) Normalized CD melting profile monitored at 290 nm for *c-KIT*$^{*}_{28}$ in the presence of PDC-PNA_8 and *c-MYC* G4 (1:1:1, red), compared with those of *c-KIT*$^{*}_{28}$ alone (black) and *c-KIT*$^{*}_{28}$ in the presence of PDC-PNA_8 (1:1, orange). Schematic structures above the spectra are for illustrative purposes only.

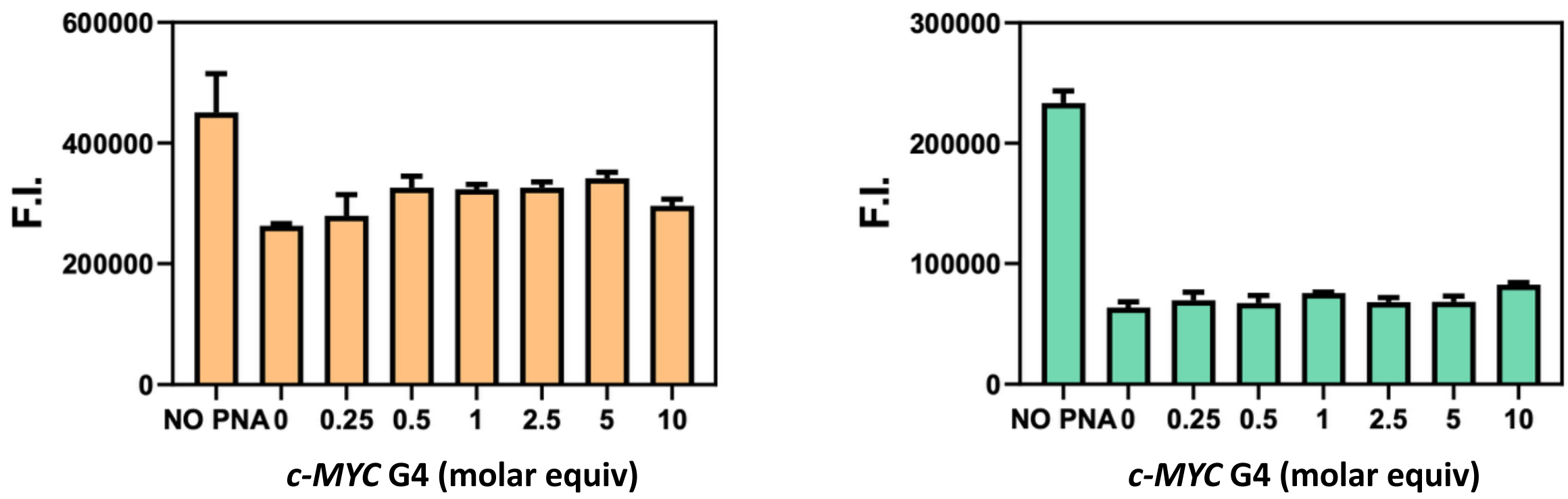

Fig. S21. Fluorescence-quenching competition experiments for (left) Cy5-labelled *c-KIT*$^{*}_{28}$/PDC-PNA_8 and (right) Cy5-labelled *c-KIT1*$_{32}$/PNA_8-PDC in the presence of 0–10 equivalents of *c-MYC* G4. “NO PNA” indicates the Cy5-labelled DNA target in the absence of both the corresponding PNA-ligand conjugate and *c-MYC* G4.

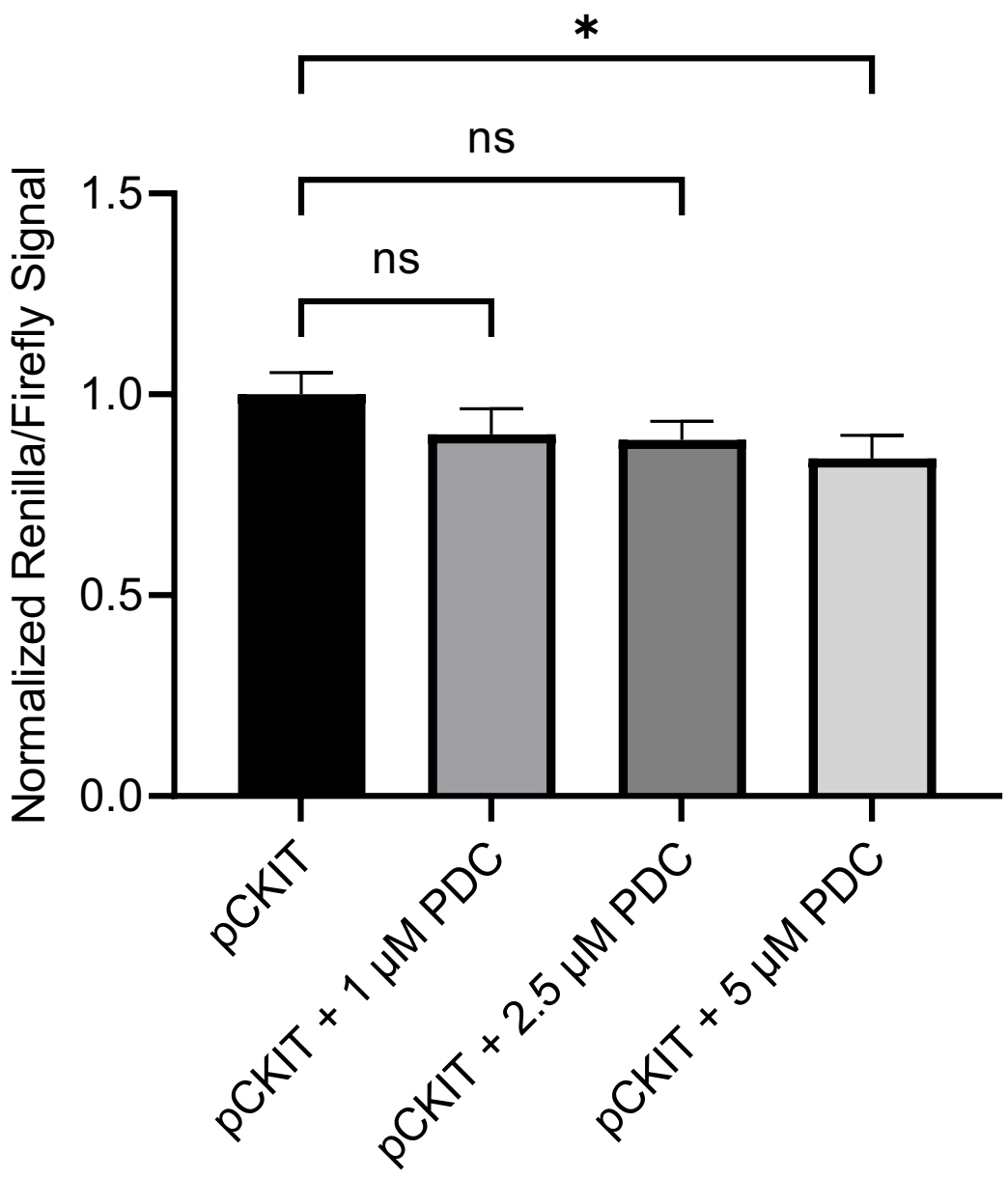

Fig. S22. Effect of PDC on *c-KIT* promoter activity. Dual-luciferase assay showing normalized Renilla/Firefly luciferase ratios in cells transfected with the *c-KIT* promoter construct and treated with PDC (0-5 μM) for 24 h. Data represent means ± SD. $N = 3$, * = p <0.05 *vs.* untreated, pC-KIT-transfected cells.

Experimental Section

Materials

CPG supports, DNA phosphoramidites, and all reagents for oligonucleotide synthesis were obtained from Link Technologies (UK). All other reagents and solvents were sourced from Merck KGaA (Germany). Fmoc-PNA(Bhoc)-OH monomers were purchased at PolyOrg Inc. (MA, USA) and stored at -20 °C. Fmoc-protected amino acids, used for resin loading, were obtained from Iris Biotech GmbH (Germany). Automated solid-phase peptide synthesis was performed on a MultiPep RSi (Intavis, Germany) using disposable polypropylene reactors with TF Frits of 5 mL from Multisyntech GmbH (Germany). Manual PNA synthesis was performed in the same type of reactors on a VacMan Vacuum Manifold (Patent Number D 342.955). A vibromatic shaker (Chem-Lab nv.) was used to stir the reactors during deprotection and coupling steps, while a mini orbital shaker (Stuart) was used during cleavage.

Oligonucleotide synthesis and sample preparation

The oligonucleotide sequences used in this study were chemically synthesized on a 1 μmol scale and purified to ensure the high level of purity required for NMR and other biophysical experiments. Synthesis was performed on a H-16 synthesizer (K&A Laborgeraete) at a 1 μmol scale in DMT-on mode, using standard β-cyanoethyl phosphoramidite solid-phase chemistry, as previously described.[47] After synthesis, oligonucleotides were detached from the support and deprotected by treatment with an aqueous solution of concentrated ammonia at 55 °C for 17 h. The combined filtrates and washings were concentrated under reduced pressure and purified using Glen-Pak cartridges according to the manufacturer's recommended protocols. After purification, the solvent was removed, and the samples were dissolved in 200 mM LiCl, followed by desalting using Amicon-15 centrifuge filter with 3 kDa molecular weight cut-off (MWCO). The purity of the isolated oligomers, as confirmed by NMR, exceeded 95%. Oligonucleotide samples were lyophilized and then dissolved in 5 mM KH_2PO_4/K_2HPO_4 buffer solution (pH 7.0) supplemented with 20 mM KCl. The concentration of each oligonucleotide was verified by measuring the UV absorption at 90 °C, considering the appropriate molar extinction coefficient values ε (λ = 260 nm) calculated using the nearest-neighbor model.[48] To ensure proper folding of the DNA sequences, samples were annealed by heating at 90 °C for 5 min, followed by slow cooling to room temperature and overnight storage at 4 °C.

Synthesis and characterization of PNA probes and PNA-PDC conjugates

All PNAs were synthesized using standard Fmoc-based solid-phase peptide synthesis (SPPS) on a MultiPep RSi instrument (Intavis), following the procedure typically employed in our laboratory (double coupling cycle, with 4 eq of monomer, 4 eq of HBTU and 8 eq of DIPEA per cycle).[49] To maximize coupling efficiency, a resin loading of 0.2 mmol/g was used. PNA sequences were synthesized with a Lys(Dde) residue (allowing late-stage functionalization at the C-terminal position) and an Arg residue (to increase overall PNA solubility) at the C-terminus. The (2-(2-aminoethoxy)ethoxy)acetyl spacer (AEEA) and bisquinolinium pyridodicarboxamide (PDC) β-Ala unit were incorporated at the N- and/or C-terminus of the PNA sequences after Fmoc deprotection of the monomer at the N-terminus and the Dde deprotection of the lysine residue at the C-terminus. The rink amide resin was initially functionalized by coupling the first Fmoc-protected amino acid (1 equivalent) in the presence of N,N′-diisopropylcarbodiimide (10 equivalents) and 3-hydroxy-1,2,3-benzotriazin-4(3H)-one (5 equivalents) for 5 h. After the loading step, the resin was thoroughly washed with DMF (3 × 5 volumes), followed by DCM (3 × 5 volumes), to remove residual reagents and byproducts, and any unreacted sites were subsequently capped with an acylating solution consisting of 50% acetic anhydride solution in DMF (2 × 15 min). After additional DMF and DCM washes, the resin was dried, and its loading capacity was determined by UV spectroscopy using the following procedure: 1-2 mg of resin was placed in a 5 mL vessel, and 3 mL of 20% piperidine solution was added for 10 min to remove the Fmoc group. The resulting solution was transferred to a cuvette, and the resin loading was calculated using the equation:

$$\mathrm{L}\left(\frac{\mathrm{mmol}}{\mathrm{g}}\right) = \frac{(\mathrm{Abs}_{290} - \mathrm{Abs}_{400})}{1.65\ \times\ \mathrm{mg}_{res}}$$

where L is the resin loading (mmol/g), Abs_{290} and Abs_{400} are the absorbance values at 290 nm and 400 nm, respectively, and mg_{res} is the resin weight in milligrams. Subsequent couplings were performed using an activation mixture of HBTU (10 equiv) and N,N-diisopropylethylamine (DIPEA) (20 equivalents) in Multipep reactors of varying volumes (1, 6, or 12 mL, depending on synthesis scale). Coupling reactions were carried out for 120-240 min. Fmoc groups were removed stepwise using 20% piperidine in DMF, while Dde groups were cleaved at the end using hydroxylamine hydrochloride and imidazole in NMP. After synthesis, PNAs were cleaved from the solid support using a TFA cocktail containing 10% m-cresol, which was added directly to the resin in the SPE tube, to ensure complete submersion of the resin (typically 50-100 µL/µmol). The cleavage reaction was allowed to proceed for 60 min. The solution was then transferred to a centrifuge tube, by applying argon flow pressure to the SPE tube, and the cleavage procedure was repeated once using the same volume of cocktail. After cleavage, the resin was rinsed with TFA, and the combined cleavage

solution was precipitated using cold (-20 °C) diethyl ether (10 volumes), incubating for 15 min at -80 °C or 2 h at -20 °C, followed by centrifugation at 7500 rpm for 7 min. The pellet was resuspended in fresh diethyl ether, and the centrifugation process was repeated at least twice to remove residual scavenger. The resulting precipitate was dried at room temperature before purification. Crude PNAs were dissolved in a minimal volume of Milli-Q water and purified by HPLC-UV on a Phenomenex Luna C18 column. The solvent system consisted of (A) 0.1% TFA in water and (B) 0.1% TFA in acetonitrile (ACN). The gradient was set to 100% solvent A for 5 min, followed by a linear gradient from 0% to 50% solvent B over 30 min, at a 4.0 mL/min flow rate. PNA purity was assessed by MALDI-TOF and LC-MS analysis. MALDI-TOF analysis was performed using an Applied Biosystems 4800 Plus MALDI TOF/TOF™ Analyzer with a matrix prepared by dissolving 100 mg/mL 2,5-dihydroxybenzoic acid (DHB) in a 1:2 mixture of Milli-Q water and ACN containing 0.1% TFA. LC-MS data were obtained using an Agilent 1100 Series system with a Phenomenex Kinetex C18 100 Å column (150 × 4.6 mm, 5 μm) maintained at 35 °C and connected to a VL quadrupole ion trap mass spectrometer. The flow rate was set to 1.5 mL/min, using the following solvent system: (A) 0.1% formic acid in water and (B) ACN. The gradient started with 100% solvent A for 2 min, followed by a transition from 0% to 100% solvent B over 6 min, ending with a 2-min flush at 100% solvent B. Analytical characterization of PNAs is provided in Fig. S23-S32.

PNA₆. Theoretical mass 1988.0 g mol^{-1}, calculated mass 1987.3 g mol^{-1}. Found (m/z): 994.2 = $[M+2H]^{2+}$, 663.3 = $[M+3H]^{3+}$, 497.9 = $[M+4H]^{4+}$, 398.5 = $[M+5H]^{5+}$.

PNA_8. Theoretical mass 2530.0 g mol^{-1}, calculated mass 2529.6 g mol^{-1}. Found (m/z): 1265.7 = $[M+2H]^{2+}$, 844.2 = $[M+3H]^{3+}$, 635.5 = $[M+4H]^{4+}$, 506.9 = $[M+5H]^{5+}$, 422.5 = $[M+6H]^{6+}$.

PNA_9. Theoretical mass 2821.0 g mol^{-1}, calculated mass 2821.2 g mol^{-1}.

Found (m/z): 706.2 = $[M+4H]^{4+}$, 565.2 = $[M+5H]^{5+}$, 471.1 = $[M+6H]^{6+}$.

PNA_{10}. Theoretical mass 3073.0 g mol^{-1}, calculated mass: 3072.1 g mol^{-1}. Found (m/z): 1537.2 = $[M+2H]^{2+}$, 1024.9 = $[M+3H]^{3+}$, 769.9 = $[M+4H]^{4+}$, 615.5 = $[M+5H]^{5+}$, 513.0 = $[M+5H]^{6+}$.

***PNA_8*-PDC**. Theoretical mass: 3149.0 g mol^{-1}, calculated mass: 3148.9 g mol^{-1}. Found (m/z): 630.8 = $[M+5H]^{5+}$, 525.9 = $[M+6H]^{6+}$, 450.9 = $[M+7H]^{7+}$, 394.6 = $[M+8H]^{8+}$.

PDC-*PNA_8*. Theoretical mass: 3149.0 g mol^{-1}, calculated mass: 3149.2 g mol^{-1}. Found (m/z): 788.3 = $[M+4H]^{4+}$, 630.9 = $[M+5H]^{5+}$, 525.9 = $[M+6H]^{6+}$, 450.9 = $[M+7H]^{7+}$.

PDC-*PNA_8*-PDC. Theoretical mass: 3768.0 g mol^{-1}, calculated mass: 3768.5 g mol^{-1}. Found (m/z): 943.0 = $[M+4H]^{4+}$, 754.7 = $[M+5H]^{5+}$, 629.1 = $[M+6H]^{6+}$, 539.4 = $[M+7H]^{7+}$.

CTRL-PDC. Theoretical mass: 3139.0 g mol^{-1}, calculated mass: 3139.2 g mol^{-1}. Found (m/z): 1047.3 = $[M+3H]^{3+}$, 785.7 = $[M+4H]^{4+}$, 628.8 = $[M+5H]^{5+}$, 524.3 = $[M+6H]^{6+}$, 449.4 = $[M+7H]^{7+}$.

PDC-CTRL. Theoretical mass: 3139.0 g mol^{-1}, calculated mass: 3139.2 g mol^{-1}. Found (m/z): 1047.3 = $[M+3H]^{3+}$, 785.7 = $[M+4H]^{4+}$, 628.8 = $[M+5H]^{5+}$, 524.3 = $[M+6H]^{6+}$, 449.5 = $[M+7H]^{7+}$.

PDC-CTRL-PDC. Theoretical mass: 3769.0 g mol^{-1}, calculated mass: 3769.3 g mol^{-1}. Found (m/z): 943.2 = $[M+4H]^{4+}$, 754.7 = $[M+5H]^{5+}$, 629.1 = $[M+6H]^{6+}$, 539.4= $[M+7H]^{7+}$.

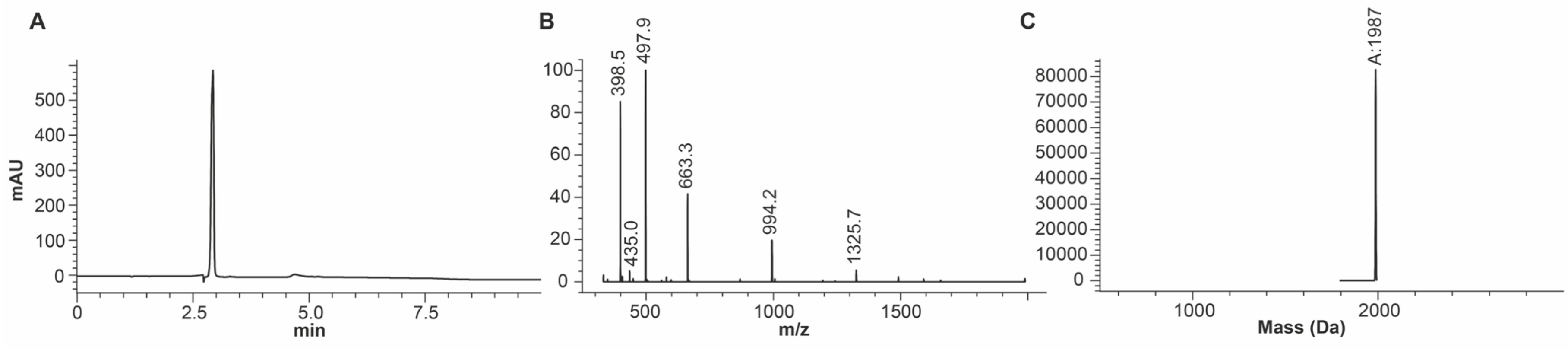

Fig. S23. PNA₆ analytical characterization. (**A**) HPLC chromatogram at 254 nm, (**B**) mass spectrum, and (**C**) deconvoluted mass of the major peak.

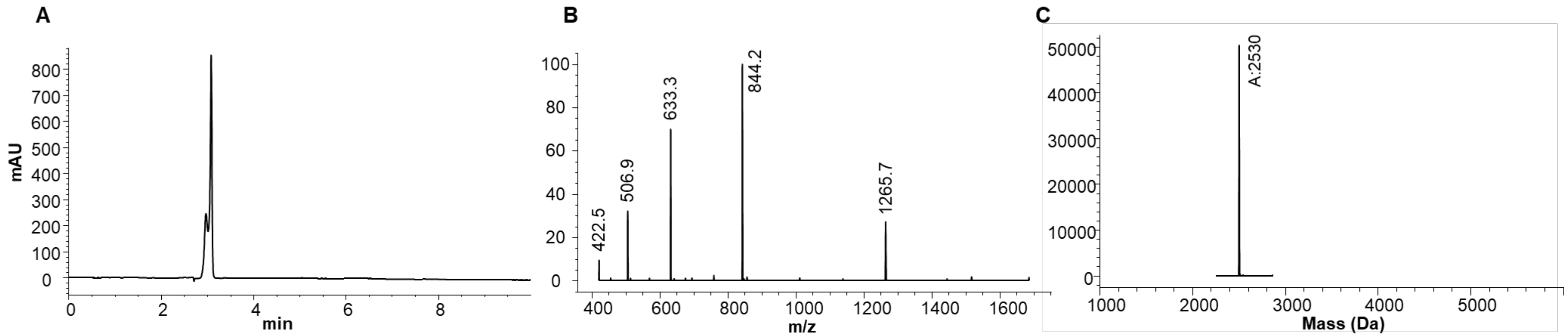

Fig. S24. PNA₈ analytical characterization. (**A**) HPLC chromatogram at 254 nm, (**B**) mass spectrum, and (**C**) deconvoluted mass of the major peak.

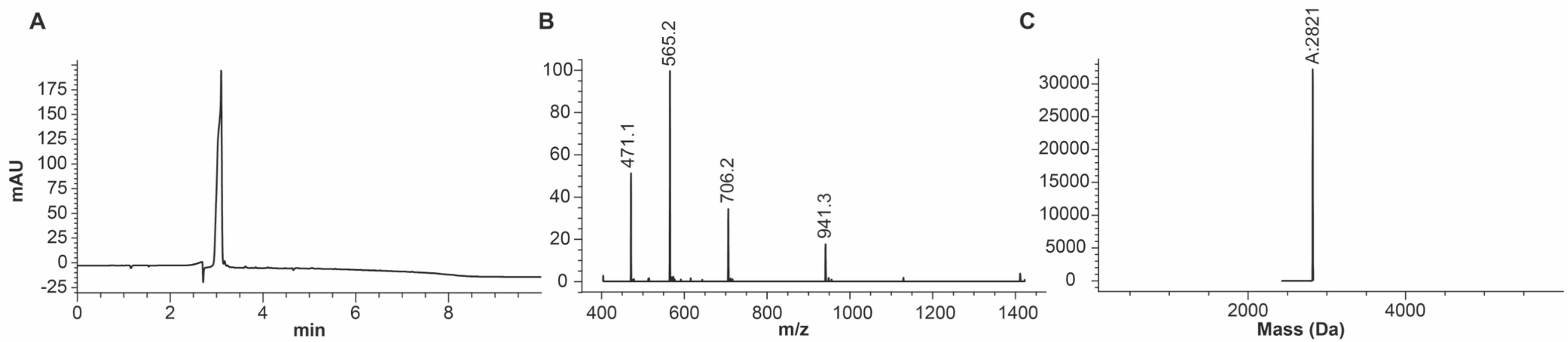

Fig. S25. PNA_9 analytical characterization. (**A**) HPLC chromatogram at 254 nm, (**B**) mass spectrum, and (**C**) deconvoluted mass of the major peak.

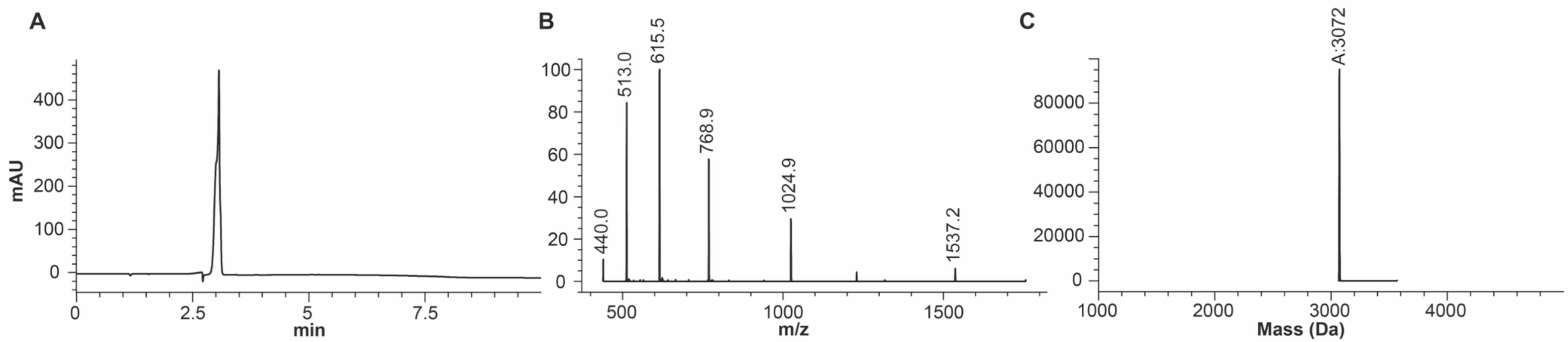

Fig. S26. PNA_{10} analytical characterization. (**A**) HPLC chromatogram at 254 nm, (**B**) mass spectrum, and (**C**) deconvoluted mass of the major peak.

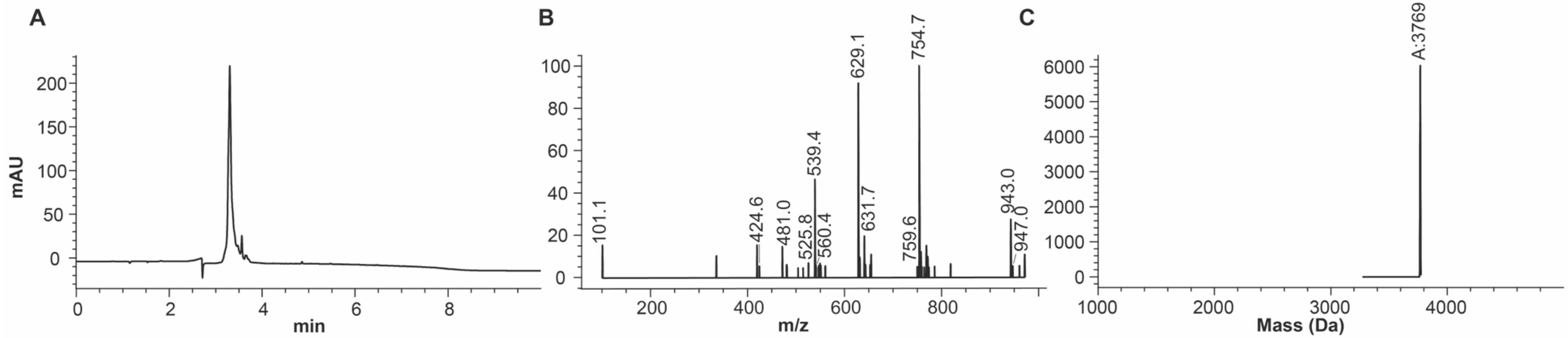

Fig. S27. PDC-PNA8-PDC analytical characterization. (**A**) HPLC chromatogram at 254 nm, (**B**) mass spectrum, and (**C**) deconvoluted mass of the major peak.

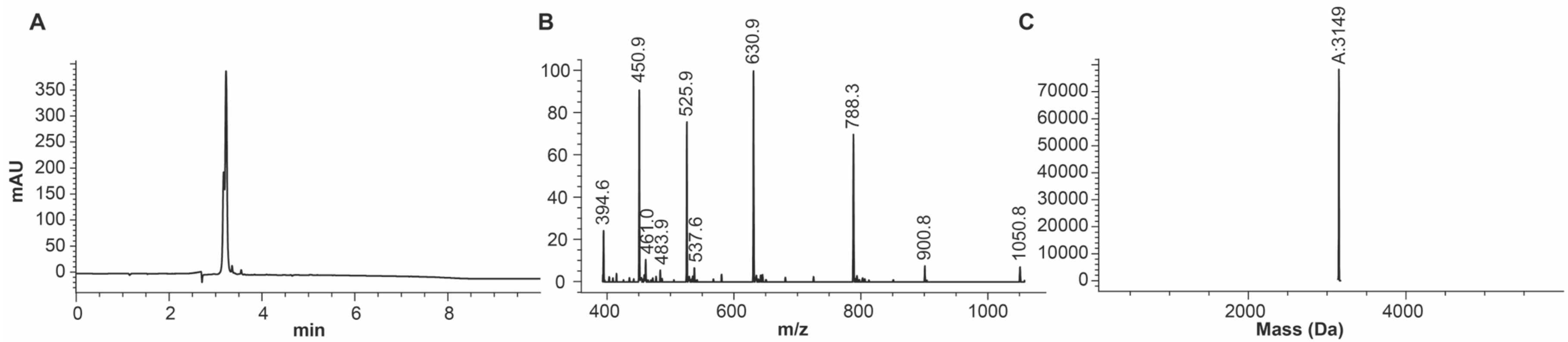

Fig. S28. PDC-PNA8 analytical characterization. (**A**) HPLC chromatogram at 254 nm, (**B**) mass spectrum, and (**C**) deconvoluted mass of the major peak.

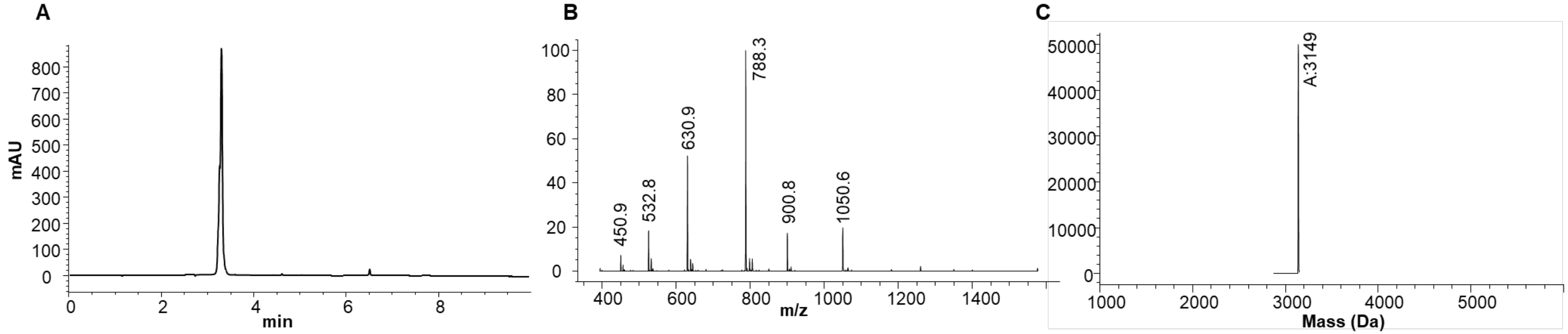

Fig. S29. PNA$_8$-PDC analytical characterization. (**A**) HPLC chromatogram at 254 nm, (**B**) mass spectrum, and (**C**) deconvoluted mass of the major peak.

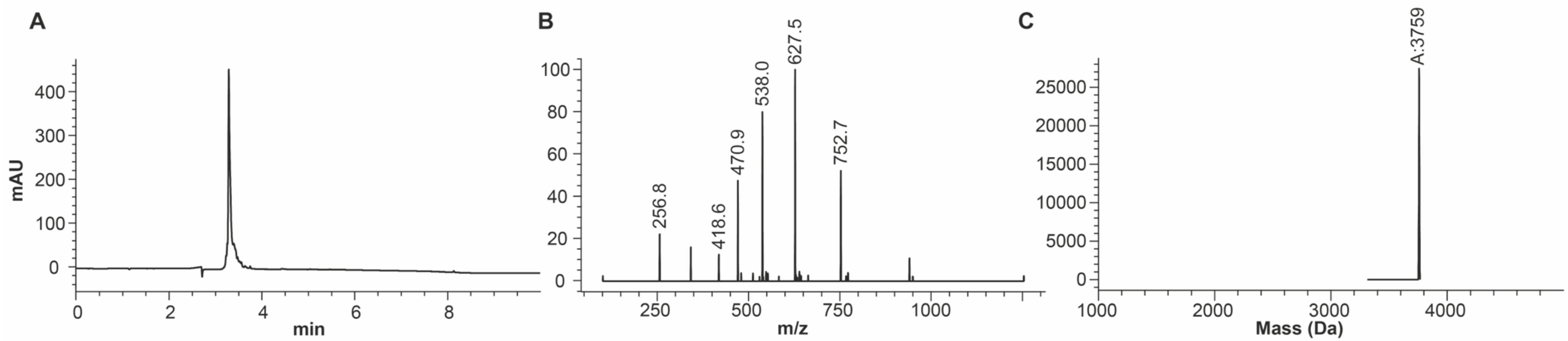

Fig. S30. PDC-CTRL-PDC analytical characterization. (**A**) HPLC chromatogram at 254 nm, (**B**) mass spectrum, and (**C**) deconvoluted mass of the major peak.

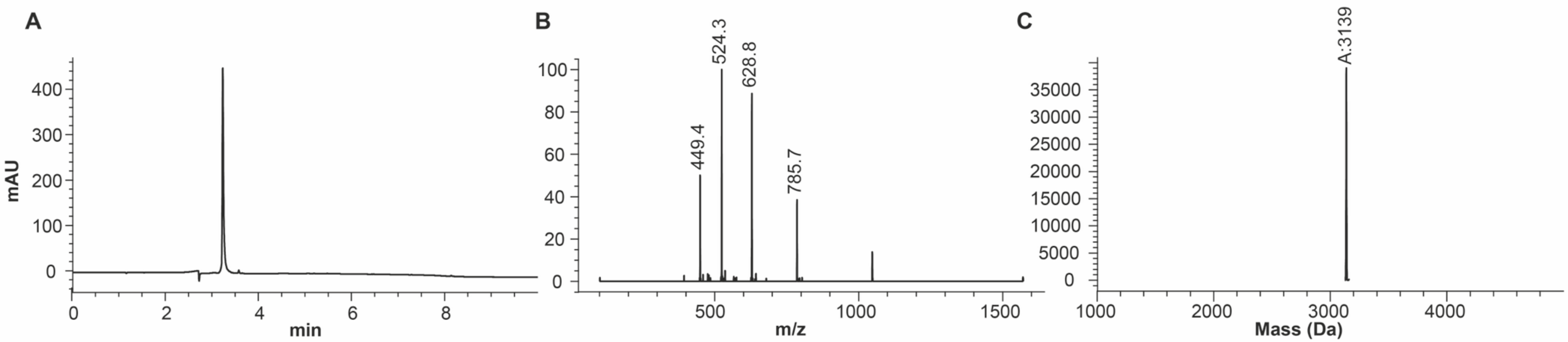

Fig. S31. PDC-CTRL analytical characterization. (**A**) HPLC chromatogram at 254 nm, (**B**) mass spectrum, and (**C**) deconvoluted mass of the major peak.

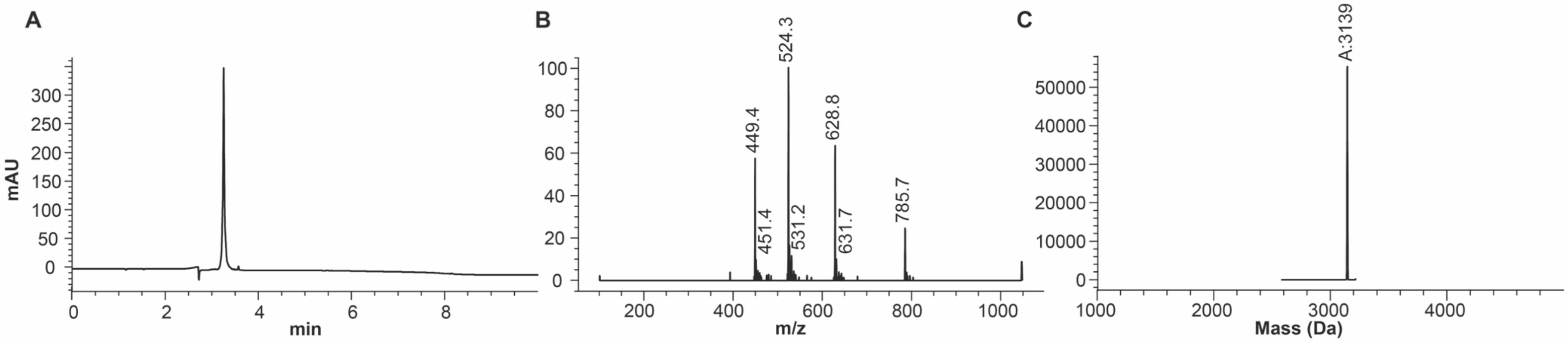

Fig. S32. CTRL-PDC analytical characterization. (**A**) HPLC chromatogram at 254 nm, (**B**) mass spectrum, and (**C**) deconvoluted mass of the major peak.

Synthesis and characterization of PDC-β-Ala

The synthetic route for PDC-β-Ala is reported in Scheme S1.

4-chloro-N,N-bis(quinolin-3-yl)pyridine-2,6-dicarboxamide [1]. Its synthesis and characterization were performed as previously described in the literature.[50]

Tert-butyl 3-((2,6-bis(quinolin-3-ylcarbamoyl)pyridin-4-yl)amino)propanoate [2]. In a screw-cap vessel, compound **1** (500 mg, 1.1 mmol, 1.0 eq) was suspended in DMSO (8 mL) and heated to 100 °C. Triethylamine (TEA, 21.0 eq, 23.30 mmol) and β-alanine tert-butyl ester (4040 mg, 22.20 mmol, 20 eq) were added to the reaction mixture. The resulting clear solution was stirred overnight at 100 °C. Progress of the reaction was monitored by NMR, and upon completion, the mixture was quenched with water. The precipitate formed was filtered using a Buchner funnel, washed with water and dried overnight under high vacuum to yield compound **2** as a white solid (500 mg, 79.8% yield). ^{1}H-NMR (400 MHz, DMSO-d_6) δ 11.44 (s, 1H, NH rotamer), 11.28 (s, 1H, NH rotamer), 9.36 (t, J = 2.4 Hz, 2H), 8.97 (dd, J = 5.7, 2.6 Hz, 2H), 8.46 (s, 1H), 8.07 – 8.02 (m, 4H), 7.78 – 7.69 (m, 2H), 7.65 (q, J = 7.9, 7.1 Hz, 2H), 7.61 – 7.52 (m, 2H), 3.49 (q, J = 6.3 Hz, 2H), 2.58 (t, J = 6.6 Hz, 2H), 1.42 (s, 9H). 13C-NMR (101 MHz, DMSO-d_6) δ 170.4, 163.1, 161.2, 156.1, 150.1, 146.1, 144.6, 131.9, 128.7, 128.3, 127.9, 127.7, 127.2, 124.1, 80.2, 38.0, 34.4, 27.7. LC-MS (ESI$^-$, DMF, rt=6.96 min) for ($C_{32}H_{28}N_6O_3$): calcd.: 562.6 g mol^{-1}. Found (m/z): 561.5 = $[M-H]^-$.

3,3'-((4-((3-(tert-butoxy)-3-oxopropyl)amino)pyridine-2,6-dicarbonyl)bis(azanediyl))bis(1-methylquinolin-1-ium) iodide [3]. In a round-bottom flask, compound **2** (500 mg, 0.89 mmol, 1.0 eq) was suspended in dry DMF. Methyl iodide (100 eq, 88 mmol) was added dropwise, and the reaction mixture was stirred at room temperature overnight, resulting in a pale-yellow suspension. Diethyl ether (Et_2O) was then added to the mixture, leading to quantitative precipitation of the product. The solid was collected by filtration using a Buchner funnel, washed five times with Et_2O, and dried under vacuum to yield compound **3** as a pale-yellow powder (433 mg, 81.6%). ^{1}H-NMR (400 MHz, DMSO-d_6) δ 11.91 (s, 1H), 11.73 (s, 1H), 10.15 – 10.08 (m, 2H), 9.66 (dd, J = 11.2, 2.4 Hz, 2H), 8.55 (d, J = 7.7 Hz, 4H), 8.23 (t, J = 8.1 Hz, 2H), 8.09 (d, J = 7.7 Hz, 2H), 7.75 (t, J = 5.6 Hz, 1H), 7.62 (s, 1H), 4.79 (s, 6H), 3.52 (dd, J = 5.6, 6.2 Hz, 2H), 2.61 (d, J = 6.2 Hz, 2H), 1.42 (s, 9H). LC-MS (ESI$^+$, DMF, rt=6.83) for ($C_{34}H_{36}N_6O_4$): calcd.: 592.7 g mol^{-1}. Found: 591.2 = $[M+H]^+$, 296.1 = $[M+2H]^{2+}$.

3,3'-((4-((2-carboxyethyl)amino)pyridine-2,6-dicarbonyl)bis(azanediyl))bis(1-methylquinolin-1-ium) trifluoroacetate [PDC-β-Ala-OH]. Compound **3** (400 mg, 0.67 mmol, 1.0 eq) was suspended in 5 mL of a 9:1 DCM/TFA solution. The suspension was stirred for 3 h, gradually transforming into a clear solution. The solvent was then removed under reduced pressure, and the residue was re-suspended in DCM. The addition of Et_2O induced precipitate formation, which was

collected by centrifugation at 6000 rpm and washed three times. PDC-β-Ala-OH was obtained as a brown powder in quantitative yield (360 mg). ^{1}H-NMR (400 MHz, DMSO-d_6, Fig. S33) δ 12.4 (s, br, 1H), 11.93 (s, 1H, NH rotamer), 11.75 (s, 1H, NH rotamer), 10.12 (dd, J = 4.3, 2.4 Hz, 2H), 9.66 (dd, J = 11.8, 2.4 Hz, 2H), 8.62 – 8.51 (m, 4H), 8.23 (q, J = 7.9 Hz, 2H), 8.13 – 8.03 (m, 2H), 7.77 (t, J = 5.6 Hz, 1H), 4.78 (s, 6H), 3.51 (q, J = 6.3 Hz, 2H), 2.62 (t, J = 6.6 Hz, 2H). ^{13}C-NMR (101 MHz, DMSO-d_6) δ 172.7, 163.2, 161.5, 149.1, 144.8, 135.7, 134.2, 132.4, 131.7, 130.4, 130.0, 129.2, 126.2, 119.2, 46.2, 34.4, 33.0. LC-MS (ESI$^+$, DMF, rt=4.3) for ($C_{30}H_{28}N_6O_4$): calc.: 536.2 g mol^{-1}. Found: 535.1 [M-H]$^-$. HR-MS (ESI$^+$, MeOH) for ($C_{30}H_{28}N_6O_4$): 535.200 g mol^{-1}.

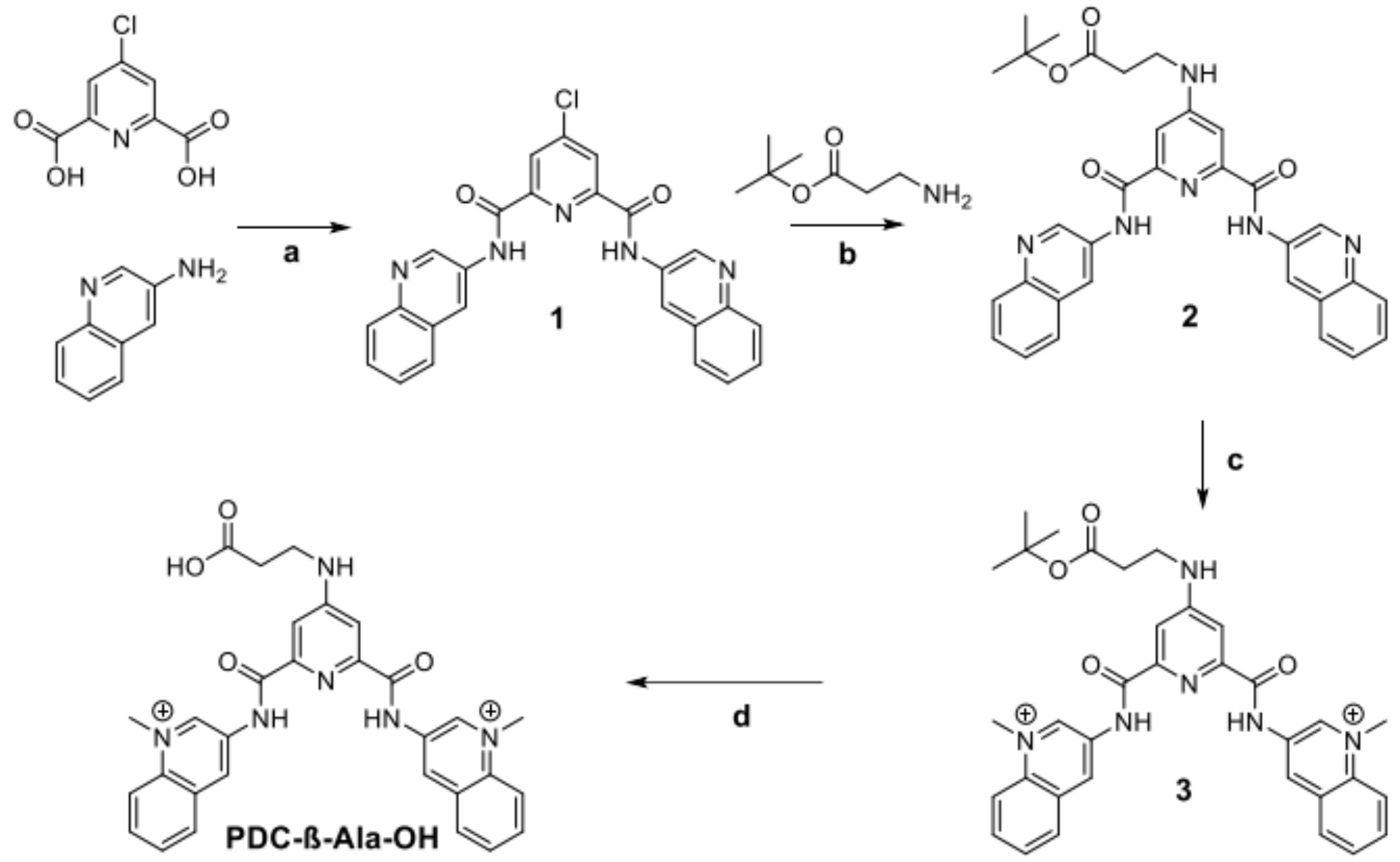

Scheme S1. Synthesis of PDC-β-Ala-OH. **a**) EDCl, HOBt, in DCM:DMF 9:1, rt, 16 h, 78%; **b**) DMSO, TEA, 90 °C, 16 h, 79.8%; **c**) CH_3I, DMF, 40 °C, 16 h, 81.6%; **d**) TFA 10% in DCM, rt, 3 h, 90%.

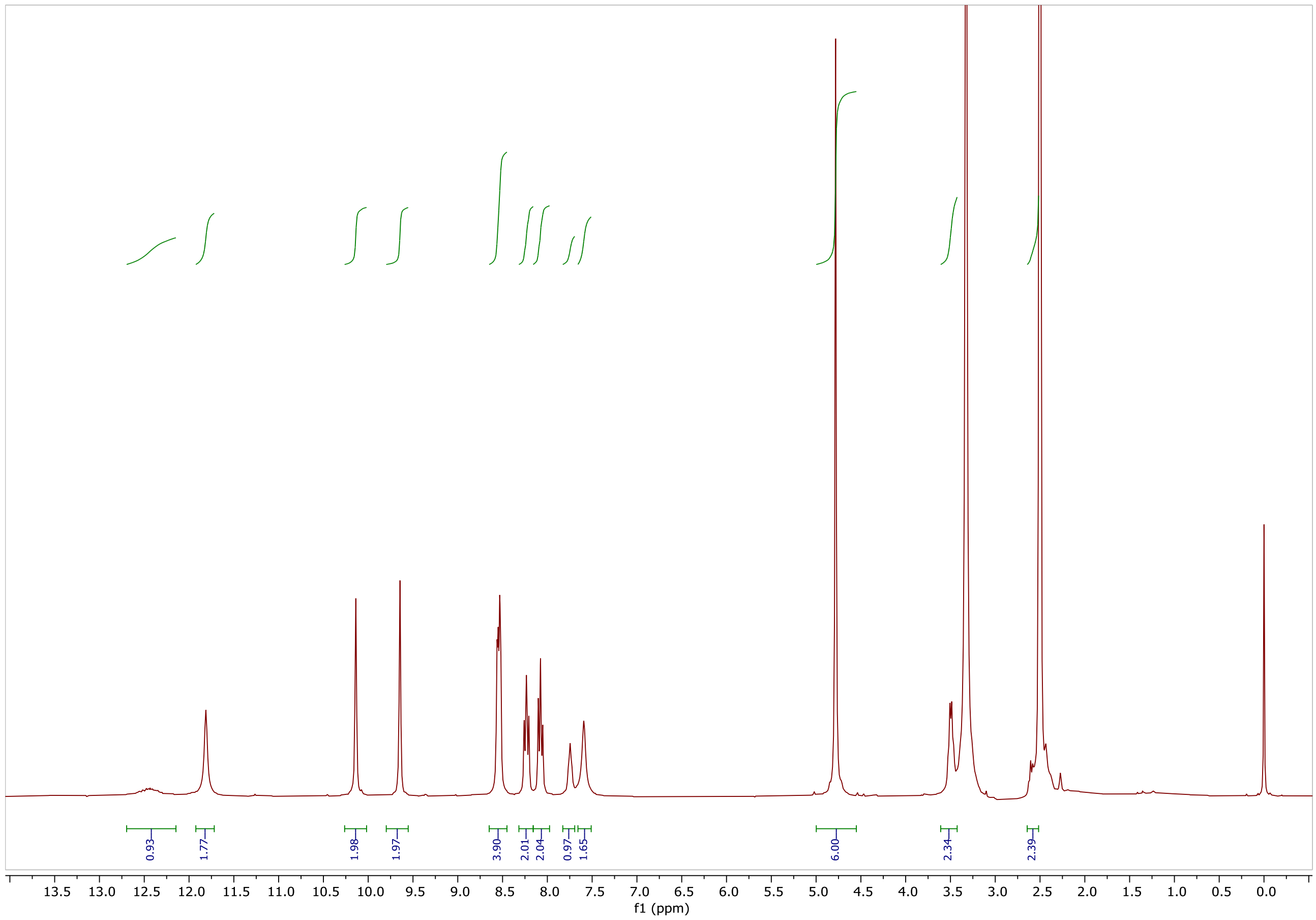

Fig. S33. ^{1}H-NMR spectrum of PDC-β-Ala, recorded on a Bruker Avance 400 Instrument, in DMSO-d_6 at 20°C.

Thermal difference spectra (TDS) analysis

TDS analysis was carried out using a Jasco V-730 UV-visible spectrophotometer equipped with a Jasco ETC-505-T temperature controller. The DNA samples were prepared at 4 μM in the buffer, annealed as described above and placed in a quartz cuvette of 1 cm path length. TDS were obtained by recording the UV absorbance spectra in the wavelength range of 220-320 nm at 25 and 95 °C and subsequently taking the difference between the two spectra.

Polyacrylamide gel electrophoresis (PAGE) experiments

Non-denaturing 20% PAGE gel was prepared using a 29:1 acrylamide/bisacrylamide solution in Tris-Borate-EDTA (TBE 1×) buffer. Oligonucleotides were annealed at 50 μM concentration in 5 mM phosphate buffer containing 20 mM KCl and loaded onto gels at a final concentration of 5 μM. To aid in sample loading, a glycerol/TBE solution was added to give a final concentration of 10% glycerol. Electrophoresis was run at 4 °C with a constant voltage of 120 V for 120-150 min. Gels were stained with GelRed® nucleic acid stain and imaged using ChemiDoc imaging system.

Circular dichroism (CD) spectroscopy experiments

CD experiments were conducted using a Jasco J-815 spectropolarimeter equipped with a PTC-423S/15 Peltier temperature controller and a quartz cuvette with a 1 cm path length. Oligonucleotide concentrations ranged from 1.5 to 2.0 μM under the experimental conditions described above. CD spectra were recorded at 20 and 90 °C in the 220-320 nm wavelength range, averaging three scans per measurement. A scan rate of 100 nm/min, with a 2 s response time and 2 nm bandwidth was used. The buffer baseline was subtracted from each spectrum. CD spectra of DNA/PNA mixtures were obtained at a 1:1 molar ratio. CD melting curves were recorded over the 20-90 °C range, by monitoring the CD signal at 264 nm for *c-KIT1* and *c-KIT1*$_{32}$, at 290 nm for *c-KIT** and *c-KIT**$_{28}$, or at both wavelengths for *c-KIT*$_{50}$, with a temperature increase of 1 °C/min. Three-dimensional CD melting curves were obtained by recording the CD spectra as a function of temperature, using the same parameters reported above, with a temperature step of 2 °C. The $\Delta T_{½}$ values were calculated as the difference in the $T_{½}$ values of the DNA structures in the presence and absence of PNAs. CD spectra and three-dimensional CD melting curves for the competition experiment were recorded under the same conditions described above, using an equimolar mixture of *c-KIT**$_{28}$, *c-MYC* G4 (5'-TGAGGGTGGGTAGGGTGGGTAA-3'), and PDC-PNA$_{8}$, each at a final concentration of 1.5 μM. All experiments were performed in duplicate, and the reported values represent the average of the measurements.

Nuclear magnetic resonance (NMR) experiments

The 1D ^{1}H NMR spectra were recorded using a Bruker Avance NEO NMR spectrometer (Bruker BioSpin, Rheinstetten, Germany), operating at 600 MHz (^{1}H Larmor frequency) and equipped with a 5-mm QCI cryo-probe set and a cooled SampleJet autosampler. DNA samples were prepared at a 0.05 mM single strand concentration in the appropriate buffer and subjected to the annealing procedure as previously described. The samples were then transferred to 5-mm NMR tubes and 10% D_2O was added before measurements. NMR spectra were acquired at temperatures ranging from 5 °C to 90 °C using excitation sculpting with gradients for water suppression. DNA/PNA mixtures were prepared in a 1:1 molar ratio. Each experiment consisted of 512 scans per spectrum, with a recovery delay of 1.5 s. Spectra were Fourier transformed, phase adjusted, baseline corrected and calibrated with respect to the 4,4-dimethyl-4-silapentane-1-sulfonic acid (DSS), used as a reference, assuming a resonance at 0.0 ppm. NMR spectra were processed and analyzed using TopSpin 4.3.0 (Bruker), and MestReNova software.

Fluorescence titration experiments

5′-Cy5.5-labelled *c-KIT*$_{50}$ was purchased from Biomers.net (Ulm, Germany). A DNA stock solution was prepared at 2 µM in 5 mM potassium phosphate buffer (pH 7.0) containing 20 mM KCl, heated at 90 °C for 5 min, and then cooled to room temperature. The annealed DNA stock solution was subsequently diluted to 20 nM in the same buffer. PDC-PNA$_8$ was prepared at 20 µM in the same buffer. Experiments were performed at a constant final DNA concentration of 10 nM, with Tween-20 added to a final 0.05%. Serial dilution of PDC-PNA$_8$ was prepared (sixteen 1:1 dilutions) and mixed with the DNA solution at a 1:1 volume ratio, resulting in final PDC-PNA$_8$ concentrations ranging from 0.3 nM to 10 µM. Samples were loaded into standard glass capillaries (NanoTemper Technologies, Munich, Germany), and the initial fluorescence intensity was measured using a Monolith NT.115 instrument (NanoTemper Technologies) at 25 °C with 100% LED power. Fluorescence intensity was plotted as a function of ligand concentration and fitted through non-linear equation to obtain the equilibrium dissociation constant (K_d) using MO.Affinity Analysis software (v2.3). Data are presented as mean ± SD of two independent experiments.

Fluorescence quenching competition experiments

Equimolar mixtures of Cy5-labelled *c-KIT**$_{28}$ (5'-Cy5-GGCGAGGAGGGGCGTGGCCGGCGCGCAG-3') and PDC-PNA$_8$, or Cy5-labelled *c-KIT1*$_{32}$ (5'-CGGCGCGCAG(Cy5)GGGAGGGCGCTGGGAGGAGGG-3') and PNA$_8$-PDC, were freshly prepared with each component at 20 µM in assay buffer containing 50 mM Tris–HCl, pH 7.2, 20 mM

KCl, 0.5% (w/v) CHAPS, and 0.05% (v/v) Triton X-100. The mixtures were placed in an Eppendorf Thermomixer, annealed by heating at 90 °C for 10 min and left cooling down at 0.5 °C/min. Samples were then distributed in a 96-well RT-PCR plate (Thermo Fisher Scientific) to a final concentration of 20 nM for both the Cy5-labelled DNA and the corresponding PNA-ligand conjugate and supplemented with *c-MYC* G4 (5'-TGAGGGTGGGTAGGGTGGGTAA-3') at 0, 0.25, 0.5, 1, 2.5, 5, or 10 molar equivalents relative to the labelled DNA. The samples were heated again at 90 °C for 10 min and slowly cooled to room temperature at 0.5 °C/min before measurement. Cy5 fluorescence was recorded at an emission wavelength of 670 nm using a CLARIOstar plate reader (BMG Labtech, Germany). Measurements were performed in technical triplicate.

HEK293T cells maintenance

HEK293T cells were maintained in DMEM Glutamax (Gibco, 10566016) supplemented with 10% fetal bovine serum at 37 °C, 5% CO_2. Cells were passaged every 3-4 days and were used under passage 20.

PNA invasion of pC-KIT plasmid

PNA was added to pC-KIT plasmid (Addgene plasmid #118983) at 10 or 50 mol equiv, followed by incubation at room temperature for 16 h to ensure maximal strand invasion. As a control, naked pC-KIT plasmid was incubated in the same manner.

Plasmid transfection and Luciferase assay

HEK293T cells were seeded in a 96-well plate (Nunc™ MicroWell™ 96-well microplates) at a density of 4 x 10^4 cells/well. 24 h after seeding, the cells were transfected with 330 ng/well PNA-invaded plasmid using FuGENE® HD Transfection Reagent (E2311) according to the manufacturer's protocol. The plates were then incubated at 37 °C for 24 h to allow the expression of the reporter genes. The expression levels of Firefly and Renilla luciferase were measured using a BMG CLARIOstar Plus luminometer, using the dual luciferase reporter assay reagents (Promega-E1980), according to the manufacturer's instructions. Luminescence data were acquired from two independent biological replicates, each performed in three technical replicates (6 replicates in total for each condition tested). The corresponding raw Firefly and Renilla luminescence values are reported in Table S3.

Table S3. Raw dual luciferase assay values.

	Firefly			Renilla		
**No transfection**	1299	3617	2403	347	451	288
**pCKIT**	4190273	2854551	714975	1521358	1051283	793749
**pCKIT O/N**	3650367	3160871	3481468	1152643	928406	1084726
**pCKIT + PNA_8 O/N**	2787268	2699806	2718327	864465	798992	843129
**pCKIT + PDC-PNA_8 O/N**	3432772	4230644	3101192	1084139	1275360	979240
**pCKIT + PNA_8-PDC O/N**	45992178	4052102	49017821	1723749	1532675	1652990
**pCKIT + PDC-PNA_8-PDC O/N**	3956309	2979736	3403450	1341315	1027954	1184336

O/N= overnight incubation

Luciferase assay for PDC treatment

HEK293T cells were seeded in a 96-well plate (Nunc™ MicroWell™ 96-well microplates) at a density of 4 x 10^4 cells/well. After 24 h, cells were transfected with 100 ng/well of the pC-KIT plasmid using FuGENE® HD Transfection Reagent (E2311) according to the manufacturer's protocol. Subsequently, PDC was added at final concentrations ranging from 0 to 5 μM, with 3 technical replicates per concentration. The plate was incubated at 37 °C for 24 h to allow the expression of the reporter genes under PDC treatment. The cells were lysed using the buffer from Promega dual luciferase reporter assay kit (Promega-E1980), followed by measurement of the expression levels of Firefly and Renilla luciferase using a BMG CLARIOSstar Plus luminometer.

References

47 E. Salvati, L. Botta, J. Amato, F. S. Di Leva, P. Zizza, A. Gioiello, B. Pagano, G. Graziani, M. Tarsounas, A. Randazzo, E. Novellino, A. Biroccio and S. Cosconati, *J. Med. Chem.*, 2017, **60**, 3626–3635.

48 C. R. Cantor, M. M. Warshaw and H. Shapiro, *Biopolymers*, 1970, **9**, 1059–1077.

49 L. De Paepe, E. Cadoni, A. Manicardi and A. Madder, *Methods*, 2023, **218**, 210–223.

50 A. Renaud de la Faverie, F. Hamon, C. Di Primo, E. Largy, E. Dausse, L. Delaurière, C. Landras-Guetta, J.-J. Toulmé, M.-P. Teulade-Fichou and J.-L. Mergny, *Biochimie*, 2011, **93**, 1357–1367.
